# Mechanisms of glycosylase induced genomic instability

**DOI:** 10.1371/journal.pone.0174041

**Published:** 2017-03-23

**Authors:** Daniel E. Eyler, Kylie A. Burnham, Thomas E. Wilson, Patrick J. O’Brien

**Affiliations:** 1 Department of Biological Chemistry, University of Michigan Medical School, Ann Arbor, MI, United States of America; 2 Department of Pathology, University of Michigan Medical School, Ann Arbor, MI, United States of America; 3 Department of Human Genetics, University of Michigan Medical School, Ann Arbor, MI, United States of America; University of South Alabama Mitchell Cancer Institute, UNITED STATES

## Abstract

Human alkyladenine DNA glycosylase (AAG) initiates base excision repair (BER) to guard against mutations by excising alkylated and deaminated purines. Counterintuitively, increased expression of AAG has been implicated in increased rates of spontaneous mutation in microsatellite repeats. This microsatellite mutator phenotype is consistent with a model in which AAG excises bulged (unpaired) bases, altering repeat length. To directly test the role of base excision in AAG-induced mutagenesis, we conducted mutation accumulation experiments in yeast overexpressing different variants of AAG and detected mutations via high-depth genome resequencing. We also developed a new software tool, hp_caller, to perform accurate genotyping at homopolymeric repeat loci. Overexpression of wild-type AAG elevated indel mutations in homopolymeric sequences distributed throughout the genome. However, catalytically inactive variants (E125Q/E125A) caused equal or greater increases in frameshift mutations. These results disprove the hypothesis that base excision is the key step in mutagenesis by overexpressed wild-type AAG. Instead, our results provide additional support for the previously published model wherein overexpressed AAG interferes with the mismatch repair (MMR) pathway. In addition to the above results, we observed a dramatic mutator phenotype for N169S AAG, which has increased rates of excision of undamaged purines. This mutant caused a 10-fold increase in point mutations at G:C base pairs and a 50-fold increase in frameshifts in A:T homopolymers. These results demonstrate that it is necessary to consider the relative activities and abundance of many DNA replication and repair proteins when considering mutator phenotypes, as they are relevant to the development of cancer and its resistance to treatment.

## Introduction

The base excision repair (BER) pathway is responsible for repairing a wide variety of oxidized and alkylated base lesions. In human cells, an estimated 10,000 lesions per day are processed through the BER pathway [1]. The multi-step BER pathway is initiated by DNA glycosylases, which search the genome and excise damaged bases. Alkyladenine DNA glycosylase (AAG; also known as MPG, methylpurine DNA glycosylase) is the sole glycosylase in its superfamily and is thus distinct from other human DNA glycosylases. In addition it is the principal glycosylase for a remarkably broad range of lesions, including hypoxanthine, xanthine, 1,*N*^6^-ethenoadenine (εA), and 3- and 7- methyladenine and methylguanine [2]. As such, AAG plays an important role in protecting the genome against the detrimental effects of these lesions.

While base lesions are often mutagenic and sometimes replication-blocking, the downstream intermediates produced during BER are themselves mutagenic and genotoxic [3–9]. When DNA damage levels are high, or glycosylase expression is elevated, AAG may produce abasic sites faster than the downstream pathway can process them [10,11]. Indeed, overexpression of AAG in yeast was reported to cause point mutations [10] and frameshift mutations in homopolymeric repeats [12,13]. Since microsatellite instability contributes to carcinogenesis [14–17] and high AAG expression has been observed in humans [18] and in cancer cell lines [19,20], the possibility that AAG may induce mutations is relevant to human disease.

The mechanisms of glycosylase-induced mutagenesis are likely to vary depending on the class of mutations. Point mutations can arise from the replication of abasic sites [6,10,21–23], and glycosylases with increased activity toward undamaged bases (i.e., increased gratuitous repair [24]) confer point mutator phenotypes, presumably by increasing the number of abasic sites [10,25,26]. The mechanism of glycosylase-induced frameshift mutagenesis in homopolymers is less well understood. During replication, polymerase slippage gives rise to altered structures with a bulged (unpaired) base flanked by duplex DNA [27]. If undetected, these nascent frameshift mutations become fixed during subsequent rounds of replication, with deletion events being more common [27–36]. Overexpression of wild-type AAG elevated deletion frameshifts by 10-fold but only increased insertion frameshifts by 3-fold [10,12,13]. The authors of this study proposed a mismatch repair (MMR) competition model in which AAG bound to bulged, undamaged bases in homopolymers and prevented efficient repair of these errors by MMR. However, since AAG catalytic activity appeared to be required for mutagenesis, and AAG overexpression remained mutagenic in strains deficient for MMR, additional mechanisms could be operative. Given the differential effects of AAG overexpression on -1 and +1 frameshift rates [13] and the ability of AAG to remove bulged bases leading to deletion by the BER pathway [37], we hypothesized that excision of bulged bases in homopolymers would generate the observed bias. This hypothesis, which we term the bulge-excision model, is differentiated from other models for glycosylase-induced mutagenesis in microsatellites by the fact that it requires a catalytically active glycosylase.

We directly examined the relationship between the base excision activity of AAG and mutagenesis, particularly in homopolymers. Our approach was to overexpress a set of human AAG variants in haploid yeast and perform mutation accumulation experiments followed by high throughput sequencing [38]. Haploid yeast are ideally suited to investigate the mechanism of glycosylase-induced homopolymer mutagenesis because yeast lack a homolog of human AAG. This simplifies the interpretation from heterologous introduction of this enzyme, and the compact genome is also amenable to calling mutations in microsatellites. This experimental system has been experimentally validated by several previous single locus studies [10,12,13]. We chose AAG variants for study that alter its biochemical properties in order to test the proposed mechanisms of mutagenesis. The E125Q mutant binds DNA similarly to the wild-type enzyme, but it is devoid of catalytic activity [39–41] and does not protect yeast against alkylation damage [25,39,42]. The Y162A mutant retains catalytic activity once a lesion is bound, but it is defective in base flipping. This defect makes it ineffectual in finding base lesions in the context of excess DNA [43], and indeed this mutant does not protect yeast from exogenous alkylation damage [39]. Finally, the N169S mutant has altered specificity, with wild-type activity toward damaged bases but elevated activity to excise undamaged purines, especially guanine [2,25,44].

We observed homopolymer mutator phenotypes in the wild-type, E125Q, and N169S strains, as well as a point mutator phenotype at G:C base pairs in the N169S strain. The observation that catalytic activity is not required for frameshift mutagenesis disproves the bulge-excision model. Instead, this finding provides additional support for the MMR competition model proposed by Samson and colleagues. While there was no correlation between mutagenesis by wild-type AAG and its catalytic properties, the catalytic properties of the N169S mutant were correlated with an increase in mutations. The largest increases in frameshift mutations were observed in the N169S strain and can be explained by this mutant enzyme’s increased excision of undamaged purines. Our data support the models of gratuitous repair by N169S AAG and competition with endogenous repair pathways. Overall, the results emphasize the importance of enzymatic specificity in restricting the deleterious consequences of inappropriate DNA repair.

## Results

### Experimental design for mutation accumulation

To distinguish between mechanisms of glycosylase-induced mutagenesis, we conducted mutation accumulation experiments in haploid *S*. *cerevisiae* under conditions similar to those previously reported for AAG-induced mutagenesis [10,13,25,44]. In cells that are fully proficient for repair, AAG overexpression has a dominant mutator phenotype. We compared cell lines containing empty vector to those that were expressing wild-type or mutant AAG (E125Q, Y162A, and N169S) at a high level. Eight lines for each construct were passaged for ~1,000 generations with bottlenecks every 20−22 generations, and unique mutations were detected by high-depth genome resequencing (~50-fold depth; see Fig A in S1 File for the analysis pipeline). Using haploid yeast allows sensitive detection of mutations even in difficult-to-sequence regions, such as homopolymers. As haploid cells are more vulnerable to negative selection against mutations, we tailored the duration of the experiment to ensure that a small number of mutations are accumulated in each line, thus minimizing the effects of negative selection.

### Point mutations

**Fig 1:**
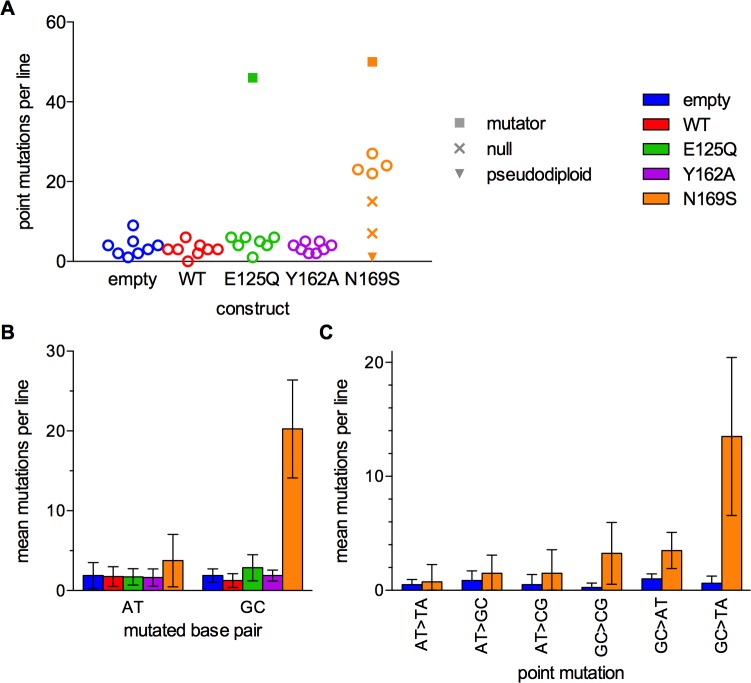
Point mutations in AAG overexpression lines. (A) The number of point mutations in each mutation accumulation line. Lines with additional mutator phenotypes are indicated with closed squares. Lines with inactivating mutations in the AAG gene are labeled “null” and marked with an ×. The pseudodiploid line is marked with a closed triangle. (B) The mean number of mutations at A:T and G:C base pairs per strain. (C) The mean number of point mutations in each category for the empty vector strain and the N169S strain. Error bars are 95% confidence intervals.

Point mutation counts were clearly increased in the N169S strain but were not detectably elevated in the other strains (Fig 1A). Two mutation accumulation lines had increased mutation counts relative to the other lines within that strain, one of the E125Q lines and one of the N169S lines. These lines appear to have acquired secondary mutator phenotypes not related to AAG overexpression, because the frequency and the spectra of mutations were distinct from the related lines (Fig D in S1 File). While neither line has a mutation in an obvious DNA repair or replication gene that easily explains their respective mutator phenotypes, there are candidate mutator mutations in each line (S2 File and S3 File). Three lines in the N169S strain had lower numbers of mutations than the rest of the N169S lines. Two of these lines had acquired different inactivating mutations in the N169S AAG construct; one introduced a premature termination codon (E268X), while the other mutated an invariant and critical arginine (R182G) [39,42,45]. The remaining line duplicated the majority of its genome except for the right arm of chromosome III. These five lines (two mutators, two null mutations in AAG, and one pseudodiploid) were excluded from further analysis. The individual lines used in this study are listed in Table B in S1 File.

Cells carrying empty vector had point mutation rates of 3×10^−10^ per base pair per generation (Table 1), consistent with other studies of haploid yeast [29,32,46–48]. We did not observe point mutator phenotypes in strains expressing wild-type, E125Q, and Y162A, although small (<3-fold) effects would have escaped detection in our experiment. The strain expressing N169S AAG had a 10-fold increased point mutation rate at G:C base pairs, with a 21-fold increase in G>T transversions. No patterns were identified in the flanking sequences, demonstrating that N169S AAG causes mutations in variety of sequence contexts (Fig F in S1 File). We compared the ratio of nonsynonymous to synonymous (*K*_α_/*K*_S_) mutations in protein coding regions to evaluate whether there was any evidence of negative selection (Table D in S1 File). These ratios did not significantly deviate from unity, which is not surprising given the low level of mutations.

**Table 1:** Point mutation rates by strain and category (×10^10^ per bp per generation).

	empty	WT	E125Q	Y162A	N169S
mutation	mean (95% CI)	mean (95% CI)	mean (95% CI)	mean (95% CI)	mean (95% CI)
AT>TA	0.7 (0.2–1.7)	0.3 (0.0–1.2)	0.7 (0.2–1.9)	0.8 (0.3–1.9)	1.0 (0.2–2.9)
AT>GC	1.1 (0.5–2.4)	1.1 (0.5–2.4)	1.1 (0.4–2.4)	0.5 (0.1–1.4)	2.0 (0.7–4.3)
AT>CG	0.7 (0.2–1.7)	0.8 (0.3–1.9)	0.4 (0.0–1.3)	0.8 (0.3–1.9)	2.0 (0.7–4.3)
AT	2.4 (1.4–4.0)	2.3 (1.2–3.8)	2.2 (1.2–3.9)	2.1 (1.1–3.6)	4.9 (2.7–8.1)
GC>CG	0.5 (0.1–1.9)	0.3 (0.0–1.5)	0.6 (0.1–2.2)	1.3 (0.4–3.1)	7.0 (4.0–12)
GC>AT	2.1 (0.9–4.1)	1.1 (0.3–2.7)	2.4 (1.0–4.7)	1.6 (0.6–3.4)	7.0 (4.0–12)
GC>TA	1.3 (0.4–3.1)	1.3 (0.4–3.1)	3.0 (1.4–5.5)	1.1 (0.3–2.7)	28 (21–37)
GC	3.9 (2.2–6.5)	2.6 (1.3–4.8)	6.0 (3.7–9.3)	3.7 (2.0–6.2)	43 (34–53)
total	3.0 (2.0–4.3)	2.4 (1.5–3.6)	3.7 (2.5–5.2)	2.7 (1.8–4.0)	19 (16–24)
lines	8	8	7	8	4

### Frameshift mutations in homopolymers

**Fig 2:**
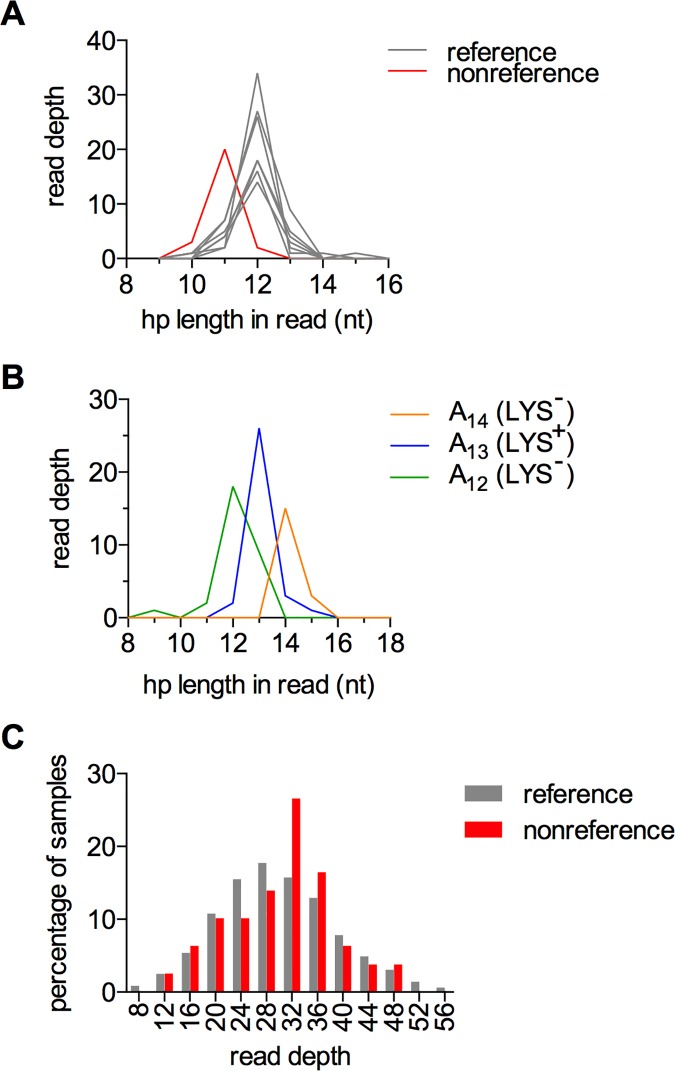
High-confidence genotyping at homopolymer loci with hp_caller. A) The hp_caller program discriminates between a mutant sample (red) and the other seven non-mutant samples in the same strain. Histograms plotting read depth at each observed homopolymer length are shown for the wild-type AAG strain for the T_12_ homopolymer at chromosome VIII:50981–50993. The mutation is in line 22556 (see Table B in S1 File for line identification). B) Genotype calls at the *LYS2* polyA reporter locus are consistent with the lysine requirements of the three strains shown (A_14_ = 22537, A_13_ = 22538, A_12_ = 22539). C) Read depth distributions at all loci with mutations were comparable for strains called as reference (gray) and non-reference (red).

Homopolymers are difficult to sequence accurately for the same reason that they are difficult for cells to replicate accurately: polymerases are prone to slippage [27]. To avoid artifacts in mutation calling, most software tools are designed to be extremely conservative in mutation calling in or near homopolymers. The samtools package called fewer indels in homopolymers than were expected on the basis of reporter assays [13,28] (Fig H in S1 File). Other callers tested were either similarly insensitive, or generated large numbers of false positives, based on visual examination of alignments. To overcome this obstacle, we developed a new homopolymer caller. This program, hp_caller, exploited specific characteristics of our dataset, namely the high sequencing depth in each line and the fact that most lines have the same genotype at any given locus, to enable sensitive and conservative homopolymer calling. The caller compares the distribution of observed homopolymer lengths in a single sample to the average distribution of the remaining samples, and calculates the probability that the single sample has the same modal observed length as the average distribution. Examples of the raw data analyzed by hp_caller are shown in Fig 2A; the sample in red is called as a mutant, while the remaining lines are called as reference. The caller can identify multiple homopolymer alleles at one locus, as long there is one predominant allele present in most strains (Fig 2B). Genome-wide, samples called as mutant have the same average read depth as samples called as reference, indicating that mutation calling by hp_caller was not an artifact of low coverage (Fig 2C). Histograms for every mutation called by hp_caller are found in S4 File, while the algorithm is described further in Supplementary Methods (S1 File).

**Fig 3:**
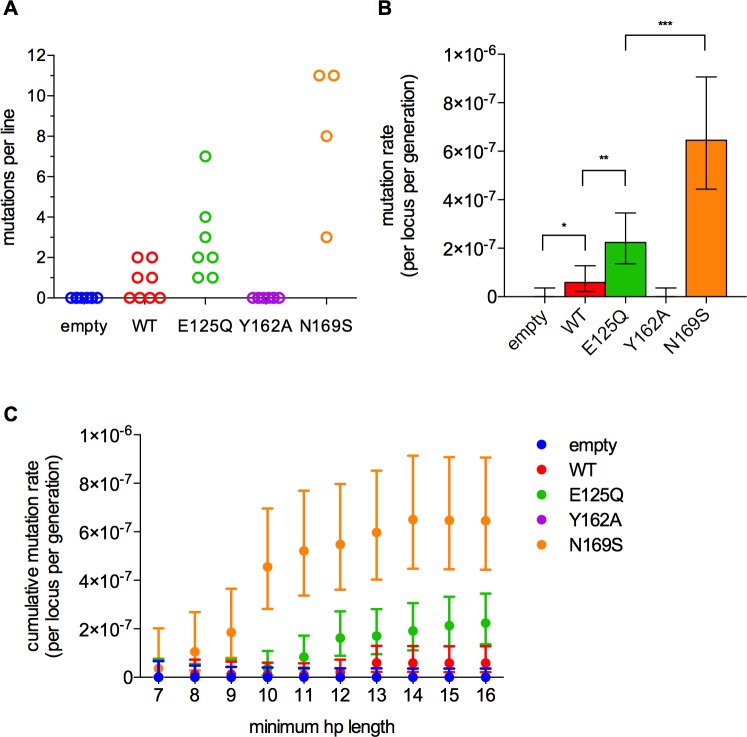
Frameshift mutations in A:T homopolymers are increased in the wild-type, E125Q, and N169S strains. (A) The number of frameshift mutations in A:T homopolymers in each line. (B) The average rate of frameshift mutations at all A:T homopolymers with lengths between 7 and 16 nt. Error bars are 95% confidence intervals calculated by the Clopper-Pearson method. Asterisks indicate p-values: *, p < 0.05; **, p < 0.005; ***, p < 0.0005; Fisher’s exact test for indicated pairwise comparisons. (C) Mutation rates in A:T homopolymers as a function of homopolymer length.

**Fig 4:**
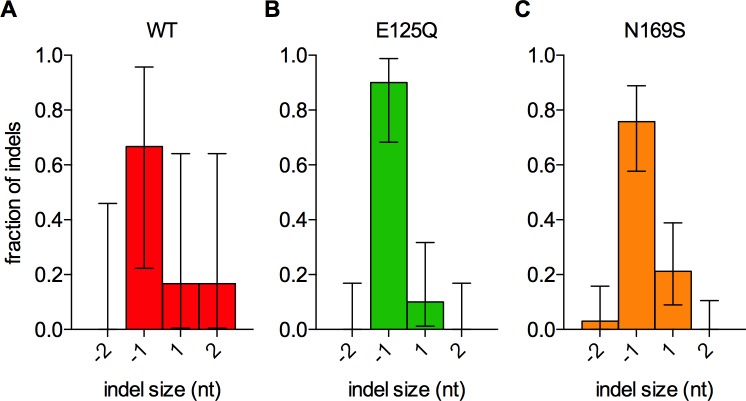
Glycosylase-induced indels in A:T homopolymers are predominantly -1 frameshifts. The fraction of indels of a given size is plotted for the (A) wild-type, (B) E125Q, and (C) N169S AAG strains. Error bars are 95% confidence intervals. The empty vector and Y162A strains are not shown because they have no indel mutations.

Seventy-nine unique mutations were identified in homopolymers by hp_caller, approximately double the number of mutations called by samtools (Fig H in S1 File). Homopolymer mutation counts per line were generally low (≤11) and were almost exclusively in A:T homopolymers (Fig 3A). The only two mutations that were observed in G:C homopolymers were in the N169S strain (histograms are included in File S4). The mean mutation rates for A:T homopolymers with lengths between 7 and 16 nt were increased in the WT, E125Q, and N169S strains (Fig 3B and Table 2). This trend held over all lengths analyzed (Fig 3C). The majority of events were -1 frameshifts in each strain, regardless of the catalytic activity of the glycosylase or its specificity for damage (Fig 4).

**Table 2:** Mutation rates in A:T homopolymers with lengths between 7 and 16 nt (per locus per generation ×10^8^).

	indels	deletions	insertions
construct	mean (95% CI)	mean (95% CI)	mean (95% CI)
empty	0.0 (0.0–3.6)	0.0 (0.0–3.6)	0.0 (0.0–3.6)
WT	5.9 (2.2–13)	3.9 (1.1–10)	2.0 (0.2–7.1)
E125Q	23 (15–36)	21 (13–33)	2.2 (0.3–8.1)
Y162A	1.0 (0.0–5.5)	0.0 (0.0–3.6)	1.0 (0.0–5.5)
N169S	65 (44–91)	51 (33–75)	14 (5.5–28)

**Fig 5:**
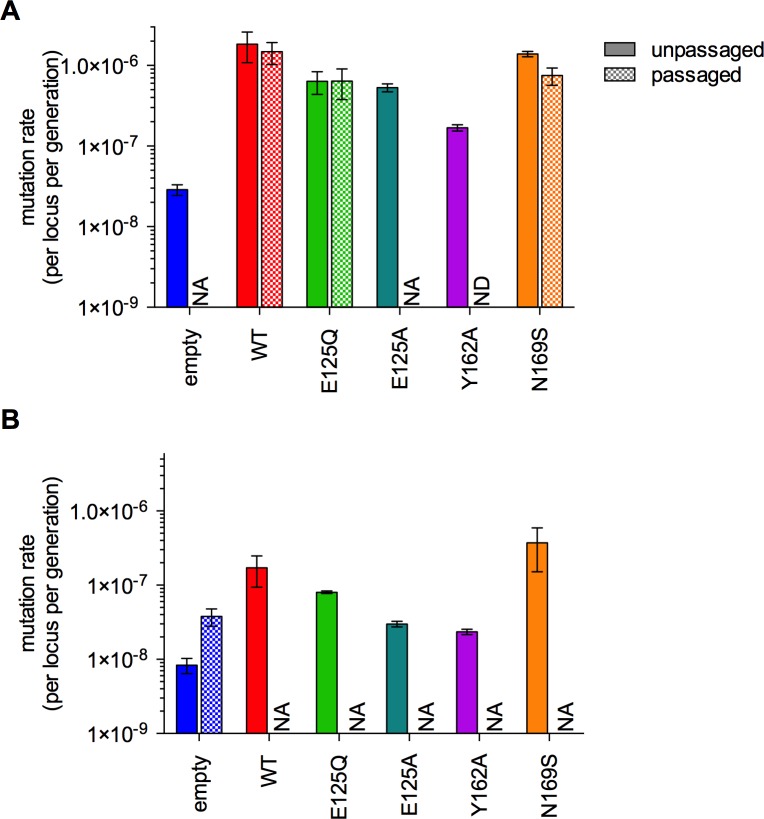
Fluctuation analysis reveals similar mutator phenotypes for E125Q and E125A AAG and preservation of mutator phenotypes during passaging. Frameshift mutation rates were measured using the *LYS2* poly-A reporter allele for -1 frameshift events in E134-derived lines (A) and +1 frameshift events in E133-derived lines (B). Mutation rates were measured by fluctuation analysis in unpassaged strains and for a subset of the lines in each strain after passaging, specifically, the passaged lines with the fewest number of mutations. Error bars indicate the standard error of the mean. Fluctuation analysis could not be performed in ending lines marked with “NA” (not applicable), because these lines did not contain the appropriate *LYS2* reporter allele (see Table B in S1 File). Fluctuation analysis was not performed (“ND”) in the passaged Y162A lines since this construct did not induce a mutator phenotype in the mutation accumulation experiments.

Western blot analysis confirmed that the different AAG variants were expressed at similar levels (Fig B in S1 File), but we were concerned that some variants could be selected against during serial passaging. Therefore, we used fluctuation analysis to assay glycosylase-induced frameshift mutagenesis using the *LYS2* polyA reporter allele in these strains. Mutation rates were similar for unpassaged and passaged lines in each strain (Fig 5), indicating that average levels of AAG-induced mutagenesis did not change during passaging.

It was surprising that E125Q and wild-type AAG exhibited similar mutator phenotypes (Figs 3 and 5), because a previous study reported that the E125A variant of AAG almost completely lacked a mutator phenotype [13]. To resolve this discrepancy, we independently created the E125A mutation in AAG and used fluctuation analysis to evaluate whether or not E125A AAG is a mutator in the *LYS2* reporter strain (Fig 5). The similar mutator phenotype accompanying overexpression of either E125Q or E125A provides strong evidence that glycosylase activity is not required for frameshift mutagenesis and suggests that these two mutant proteins behave similarly. Whereas both are catalytically inactive [41], the E125A mutant was reported to be unable to bind to bulged, undamaged bases [13].

**Fig 6:**
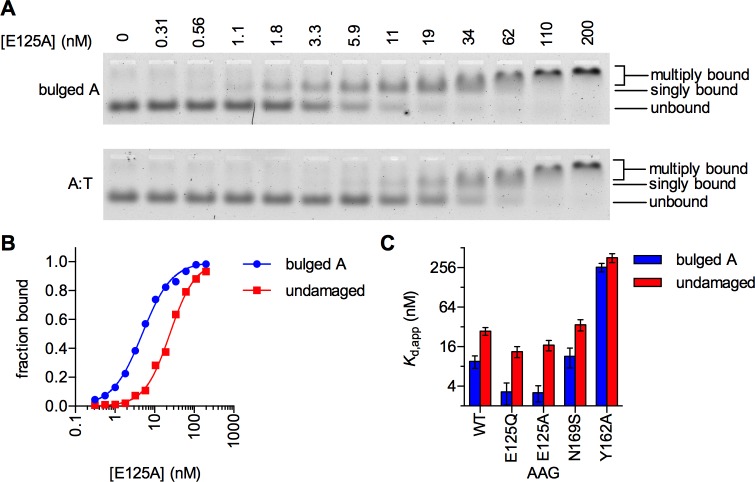
The wild-type and mutant AAG enzymes exhibit different affinities for bulged and duplex DNA. (A) Sample EMSA gel image for E125A. Singly and multiply bound complexes were combined in calculating the fraction of DNA bound, because these species are not cleanly separated. (B) Example binding curves for the E125A mutant. (C) Apparent *K*_D_ values were determined by EMSA for the indicated substrates, containing a centrally located, bulged undamaged adenosine, or fully duplex DNA (undamaged). n = 3; error bars are SEM. E125Q and E125A both exhibit tighter binding than wild-type to a bulged site, whereas the Y162A exhibits much weaker binding and does not distinguish between bulged and intact duplexes. Binding data for all constructs is shown in Fig M in S1 File.

To directly compare the DNA binding affinity of the E125Q and E125A mutant proteins, and to compare this affinity to the other AAG variants, we used electrophoretic mobility shift assays (EMSAs). Under the conditions tested, the wild-type and mutant AAG proteins bound to the A-bulge-containing DNA, which mimics a single nucleotide polymerase slipping event, with an observed affinity that is approximately 3-fold tighter for the bulge than for perfectly matched duplex DNA of the same sequence (Fig 6). As there are many overlapping binding sites on the undamaged oligonucleotide duplex, this equilibrium binding result indicates that the microscopic K_d_ for binding to a bulged site is much tighter than for binding to a typical undamaged site. Nevertheless, this assay allows us to conclude that the E125Q and E125A mutant proteins have almost identical affinity for bulged DNA. The N169S and wild-type AAG also bound specifically to bulged DNA, albeit with weaker preference for the bulged DNA (Fig 6). In contrast, Y162A AAG was unable to recognize the bulged DNA. The observation that Y162A binds with the same affinity to bulged and duplex DNA is explained by nonspecific duplex binding with both oligonucleotides.

### No strong patterns in the locations of glycosylase-induced mutations

We investigated the possibility that glycosylase-induced mutagenesis might be biased towards loci with specific characteristics. We analyzed flanking sequences (Fig F in S1 File) [49], annotated genomic features (Fig J in S1 File) [50], replication timing (Fig K in S1 File) [51], and transcription (Fig L in S1 File) [52,53]. These analyses were restricted to strains with appreciable numbers of each class of mutation (96 point mutations in the N169S strain and 53 frameshift mutations in the E125Q and N169S strains). We did not observe any correlations between mutated loci and the characteristics we tested, indicating that glycosylase-induced mutagenesis is not strongly dependent on any of these features. The absence of strong correlations is consistent with the idea that AAG has broad access to the genome, but given the small number of mutations that were observed, it is possible that there are some biases that were not detected.

### Variation in plasmid copy numbers

**Fig 7:**
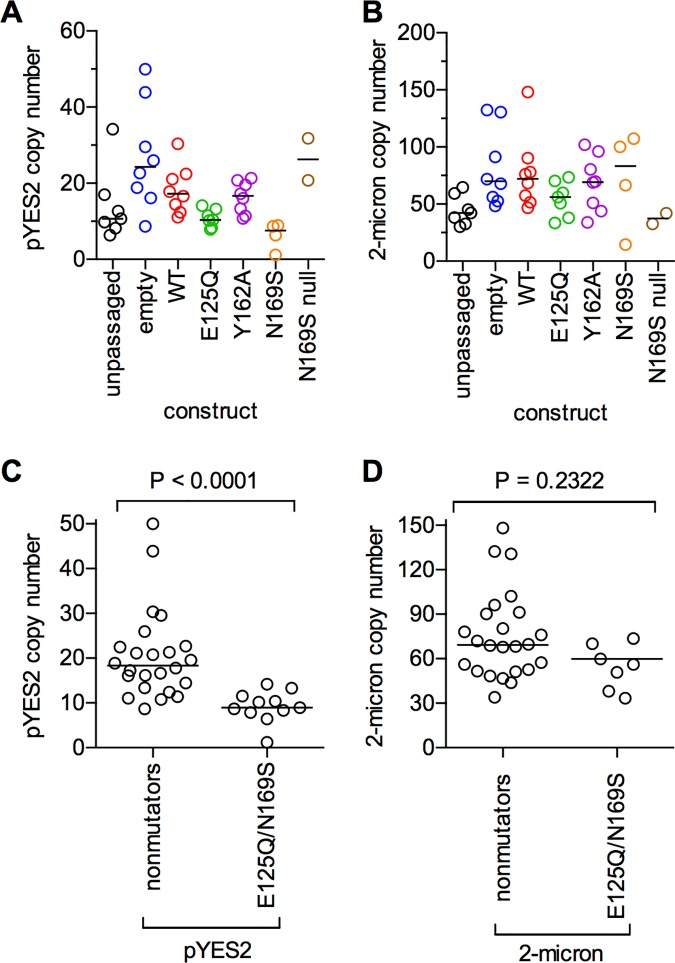
Strains expressing mutator glycosylase alleles have decreased pYES2 expression plasmid copy numbers. (A) pYES2 copy numbers for the parental (unpassaged) strains, as well as each of the ending strains. The two lines with inactivating mutations in the AAG gene are shown as the strain “N169S null”. (B) Endogenous 2-micron circle copy numbers for the parental strains and ending strains. (C) U-test comparing the distribution of plasmid copy numbers between the mutator strains (E125Q + N169S) and the nonmutator strains (empty + WT + Y162A) for the pYES2 expression vector. (D) U-test comparing the copy numbers of the endogenous 2-micron circle for mutator and nonmutator strains. Horizontal lines in all panels indicate the median of each distribution.

Lastly, we analyzed the copy numbers of the endogenous 2-micron circle and the pYES2 expression vector. The copy number of the pYES2 expression vector appeared to be lower in the E125Q and N169S strains, which had the strongest mutator phenotypes (Fig 7A). In comparison, the endogenous 2-micron circle copy number did not seem to be affected (Fig 7B). To test this hypothesis, we grouped all the strains with low or non-existent mutator phenotypes (empty vector, wild-type, and Y162A) and the two strains with mutator phenotypes (E125Q and N169S). The copy number of the pYES2 expression vector was significantly decreased in the mutator strains relative to the non-mutator strains, while the endogenous 2-micron circle was unaffected (Fig 7C). The inverse correlation between expression plasmid copy number and mutator severity suggests that passaged cells experienced negative selection if they harbored a mutator glycosylase.

## Discussion

This study was designed to examine the contributions of catalytic activity and specificity to glycosylase-induced mutagenesis in an unbiased manner throughout the genome. Several AAG variants induced frameshift mutations in homopolymers, while N169S AAG induced point and frameshift mutations. We consider first the mechanisms for frameshift mutagenesis that apply to most AAG variants and second the mechanisms that are specific to the N169S mutant.

### Mechanisms of AAG-induced frameshift mutagenesis

**Fig 8:**
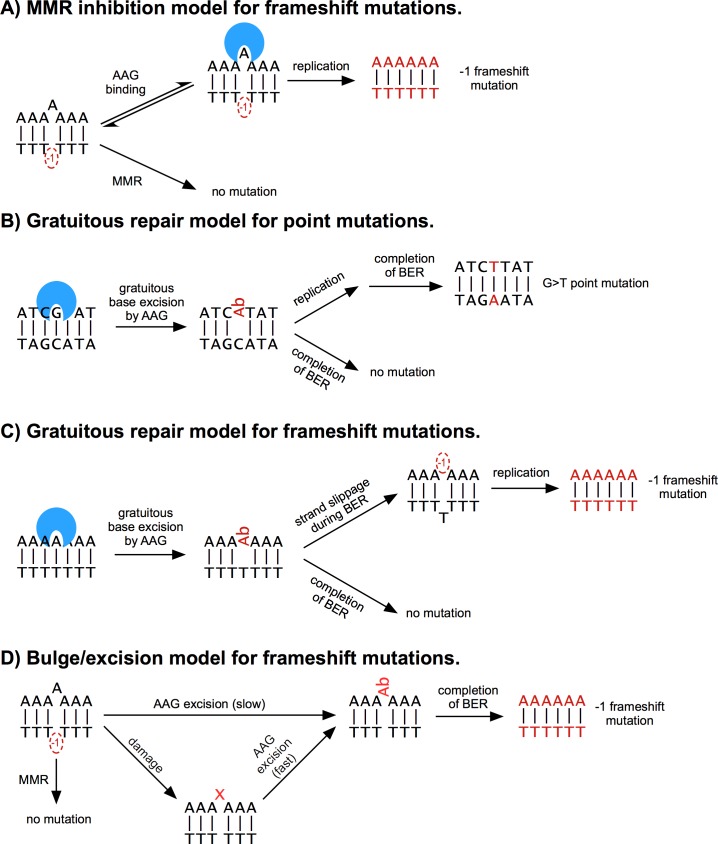
Models for glycosylase-induced mutagenesis. For simplicity, only the most relevant pathways and intermediates are shown. Replication (not shown) occasionally gives rise to bulged species in models A and B (see Fig O in S1 File for more detailed models). (A) The MMR interference model proposes that glycosylase binding to bulged, undamaged bases prevents MMR from repairing the bulged base. The pathway shown is for -1 frameshift events, but +1 frameshifts can be stabilized by the same mechanism. (B) The bulge-excision model proposes that bulged bases are deleted by the BER pathway and could be elevated by base damage. This pathway produces only -1 frameshifts, because nascent +1 frameshifts (not shown) would be faithfully repaired. (C) The gratuitous excision model postulates that removal of undamaged bases is mutagenic. In the case of the N169S mutant, most of the gratuitous excision occurs at undamaged guanines. In yeast, abasic sites are paired with A and C during replication. Pairing with C is non-mutagenic in this case, and is not shown. (D) Gratuitous excision in homopolymers effectively increases the number of times that a homopolymer is replicated, thus increasing frameshift rates. Both -1 and +1 frameshift events would occur by this mechanism, but only a -1 frameshift is shown.

The prevailing model for glycosylase-induced frameshift mutagenesis is the MMR competition model (Fig 8A; [13]) in which glycosylase binding to bulged nucleotides blocks access to MMR enzymes. This mechanism predicts that inactive glycosylases will be mutagenic. In contrast to MMR competition, the alternative bulge-excision model requires catalytically active glycosylases. We tested this model, which involves excision and deletion of the bulged base to preferentially generate -1 frameshift events, using wild-type and inactive (E125Q) AAG (Fig 8B; [37,54]).

To test the bulge-excision model, we used previously characterized variants of AAG that lacked catalytic activity but retained ability to bind to bulges (E125Q/E125A), or that retained glycosylase activity but lost the ability to bind specifically to bulges (Y162A), and compared these to the wild-type AAG. Consistent with the previous report [13], overexpression of wild-type AAG caused an increase in frameshift mutations in A:T homopolymeric regions. In contrast to the earlier study [13], we found that inactive AAG also acted as a frameshift mutator in A:T homopolymers.

Since the previous study utilized E125A AAG and we used E125Q AAG, we directly compared the biochemical and cellular properties of these two mutants. The two mutants had similar affinities for single A-bulges (Fig 6A), similarly impaired (10^5^-fold) glycosylase activity [41], were similarly expressed (Fig B in S1 File), and conferred similar increases in -1 frameshifts in vivo (Fig 5A). Since the E125Q and E125A mutants behaved similarly in four different experiments, we conclude that inactive AAG confers a frameshift mutator phenotype.

The Y162A variant was investigated as an additional test of the role of bulge binding in mutagenesis. This mutation destabilizes the flipped-out complex by a factor of greater than 100-fold, without changing the rate of εA excision [43]. Here we show that the Y162A mutant is incapable of binding to a single nucleotide bulge site (Fig 6A). Weakened bulge binding predicts that Y162A would not interfere with other DNA repair processes, and indeed, overexpression of Y162A AAG was less mutagenic than other AAG constructs in both mutation accumulation (Fig 3) and fluctuation analysis (Fig 5) experiments.

Since the strong bias towards deletions proved to be an intrinsic property of DNA replication in yeast [28–32,34–36,55], and mutagenesis was not dependent on catalytic activity, the bulge-excision model can be rejected. Frameshift mutagenesis by inactive AAG is instead consistent with a broad class of models in which AAG binding to nascent bulged nucleotides during replication prevents their repair. Nascent bulged nucleotides can be repaired by a number of pathways, including polymerase proofreading [55] and MMR. However, previous work demonstrated that competition with MMR is the most frequent event, because the number of AAG-induced frameshift mutations is greatly decreased in MMR deficient backgrounds [13]. This interpretation is also supported by the fact that AAG and the MSH2-MSH6 complex have similar *K*_D_ values for bulged bases (8 and 28 nM [56], respectively).

### Mutagenesis associated with gratuitous repair

Wild-type AAG has low, but detectable activity to excise normal, undamaged purines from DNA [57], and it has been reported that overexpression confers a modest increase in point mutations [10]. The gratuitous excision of undamaged guanine is suppressed by a steric clash with N169 in the active site, and mutation to N169S opens up the active site to increase glycosylase activity by 3-fold and 30-fold for excision of A and G, respectively [2,25,44]. The N169S variant increased point mutations in G:C pairs by 10-fold and in A:T pairs by 2-fold (Fig 1 and Table 1). The largest class of point mutations observed in this case was G→T transversions, which could be explained by replication of an abasic site to insert an A following gratuitous repair (Fig 8C; [23,58–60]). This specific mechanism of mutagenesis by abasic sites has been previously invoked to explain the mutator phenotypes induced by other glycosylases with high activity on undamaged bases [10,26,61].

The N169S variant also induced high levels of frameshift mutations in A:T homopolymers. Unlike other variants tested, the N169S frameshift mutation spectrum included a significant increase in +1 frameshift events. This result was surprising because base excision by a glycosylase cannot insert a base. To explain this result, we propose that N169S gratuitously initiates BER in homopolymers, as it does in heterogeneous sequences. Since completion of BER requires DNA polymerization, there is an opportunity for slippage and realignment with concomitant introduction of a bulged (unpaired) base (Fig 8D). In addition to A:T frameshift mutations, the N169S strains also had two -1 deletions in G:C homopolymers, while all other strains had no mutations in G:C homopolymers. This small number of events is indicative of a large increase in N169S-induced frameshifts in G:C homopolymers, because there are many fewer sites in the genome (Fig G in S1 File).

### Role of selection in mutation accumulation experiments

In comparison to mutator phenotypes in microsatellite-unstable human cancers [62], the mutator phenotype induced by overexpression of AAG was fairly mild. Nevertheless, there are two mechanisms by which selective pressure could influence the calculated mutation rates. The first would be if deleterious mutations were selected against, and therefore the number of mutations observed would be an underestimate. We mitigated the effects of negative selection by growing yeast on plates and keeping the mutation load low in each passaged strain. Comparison of synonymous to nonsynonymous mutations suggested that the point mutations that were observed in the genome were not selected against (Table D in S1 File). However, the second type of selective pressure would be if the cells were to reduce or eliminate the expression of the glycosylase variant. This would also lead to an underestimate of the mutator phenotype. There was clear evidence for selection against the AAG expression vectors. Both E125Q and N169S AAG showed significant reduction in the copy number of the multicopy expression plasmid (Fig 7). The selection against the N169S construct was particularly striking, because the enzyme was inactivated by homoplasmic point mutations in 25% (2/8) of the independently passaged cell lines. These observations are a cautionary tale for researchers performing mutation accumulation experiments or overexpressing putatively innocuous proteins (e.g., DNA repair proteins) from yeast high-copy plasmids.

### Implications of glycosylase-induced mutagenesis

This work used unbiased mutation accumulation experiments to validate previous observations of mutator phenotypes induced by overexpression of DNA glycosylases. Galactose-driven expression of AAG in yeast yields approximately 13,000 molecules per cell [42], which is similar to the higher end of the range estimated for normal human cells (500–10,000 molecules per cell) [63,64]. Although the total mutations per cell would therefore be comparable, the absolute mutation rate is expected to be smaller in human cells because the genome is much larger than the yeast genome. The finding that wild-type AAG expression is barely mutagenic suggests that AAG has evolved strategies to avoid causing mutations even when highly expressed. This is in stark contrast to the much stronger mutator phenotypes that have been observed for overexpression of yeast [10] and bacterial alkylation repair enzymes [57].

The E125Q and N169S variants were more mutagenic than the wild-type AAG, underscoring the potential of single amino acid changes to confer mutator phenotypes. These mutator effects were dominant, because the yeast were not DNA repair deficient. Whereas many loss-of-function alleles are tolerated due to redundancies between repair pathways, the DNA repair proteins are susceptible to gain-of-function mutator phenotypes. We attribute this to their privileged access to genomic DNA. Given that humans have a multitude of DNA repair enzymes, there are many potential mutations that could confer a mutator phenotype. Indeed, a growing number of mutator glycosylase alleles have been discovered. For AAG, the Y127I allele also confers a strong frameshift mutator phenotype [13]. The D239Y allele of endonuclease III homolog (NTHL1) stably binds, but fails to excise damaged bases [65], and the G199S allele in thymine DNA glycosylase stably binds abasic sites, blocking repair [66]. In addition, the N204D and Y147A mutants of uracil DNA glycosylase allow for gratuitous excision of undamaged pyrimidines [26], and the A145G and H151A/Q mutations allow thymine DNA glycosylase to initiate gratuitous repair at normal A:T sites in DNA [61]. Mutator alleles of pol β have also been observed in human cancers [9,67].

While the mutator phenotypes we observed in our experiment were relatively minor [62], with increases in mutation rates ranging from 5- to 50-fold, they may still be sufficient to increase an individual’s lifetime cancer risk. In humans, alleles that confer strong mutator phenotypes are readily detected in genome-wide association studies of cancer patients. However, because of the many different alleles that generate less dramatic mutator phenotypes, these studies may not identify all mutator alleles that contribute to carcinogenesis. Continued biochemical and genetic study of allelic variants in DNA repair proteins is thus important to comprehensively identify sources of genetic instability. This information may ultimately be useful for assessing individual lifetime cancer risk and for guiding cancer treatment choices.

## Materials and methods

### Yeast strains and media

The yeast reporter strains (E133 and E134) were provided by D. Gordenin [28]. The pYES2 constructs for expression of N169S and Y162A AAG were obtained from M. Wyatt [25,44]. The construct for expression of wild-type AAG was previously described [42]. Site-directed mutagenesis was used to introduce the following point mutations: G373C for E125Q; T484G and A485C for Y162A; and A506G for N169S. Each construct was verified by sequencing of the entire AAG coding sequence. Plasmids were transformed into yeast by the standard lithium acetate procedure. Single clones were picked and maintained as patches until the start of the mutation accumulation experiment. At the start of the mutation accumulation experiment, each starting strain was streaked for isolation on CSM-URA with 2% galactose. A single colony was picked and restreaked on CSM-URA with 2% galactose (and used to prepare a glycerol stock). From this plate, eight independent colonies were picked and streaked separately on CSM-URA with 2% galactose. Each line was passaged independently, in parallel, with bottlenecks every 20–22 generations, for a total of 1000 generations. The number of generations at each plating was verified by independently resuspending 4–5 colonies in water and determining the number of cells per colony by measuring OD_600_. At the end of passaging, each line was patched onto CSM-URA with 2% dextrose. From this plate, samples were frozen in YPD with 15% glycerol at -80°C for preservation. Overnight cultures were grown for each line and used to inoculate 25 mL of CSM-URA with 2% dextrose at OD_600_ 0.1; these cultures were grown to an OD_600_ of 0.5 before harvesting. DNA was purified with a Qiagen DNeasy Blood and Tissue Kit (#69504). Library preparation (mean insert size of 500 bp) and Illumina sequencing (100 bp paired end) were performed by the University of Michigan DNA Sequencing Core Facility.

### Alignment and mutation calling

An overview of data processing is shown in Fig A in S1 File. Similar to other pipelines [29,47,48], reads were aligned to the yeast genome (SacCer3) using bowtie2 [68] (parameters in Table A in S1 File). Alignments were marked as not unique if the next-best alignment had a score within seven points of the best alignment (based on the distribution of XS scores calculated by bowtie and the physical meaning of a score greater than -7). Duplicates were marked with MarkDuplicates in picard version 1.110 (http://broadinstitute.github.io/picard). Reads were realigned around indels using the IndelRealigner tool in GATK version 3.1-1-g07a4bf8 [69]. Samtools v0.1.19+ was used to construct a pileup and call mutations, assuming haploid genomes [70] (parameters in Table A in S1 File). Calls were filtered for unique mutations using custom Perl scripts. Unique mutations were defined as those occurring in no more than three lines out of the 47 lines sequenced (see the allele frequency spectrum in Fig C, part A, S1 File). Additionally, loci with unique mutations were required to have data in at least 44 out of the 47 total lines to avoid false positives. We also filtered out clusters of point mutations occurring within 20 bp of one another in a single line. Finally, point mutations were then filtered on QUAL score with a threshold of 100 based on the distribution of scores and visual examination of mutations in each QUAL score bin. Indels in homopolymers were called with the custom software package hp_caller, which is described in more detail below and in the Supplemental Methods in S1 File. At the end of processing, mutations were evenly distributed across chromosomes and show no evidence of tightly spaced clusters (Fig E in S1 File).

### Mutation calling at homopolymers

We did not achieve the desired level of sensitivity and accuracy for mutations at homopolymers using samtools or other tools. Hence, we developed a set of tools that utilize the characteristics of our experiment to call homopolymers with as much sensitivity and accuracy as possible. The programs are written in Perl and are available at https://github.com/dangenet/hp_caller.

Briefly, at each homopolymer locus, hp_caller compares the distribution of homopolymer lengths in reads from a given sample to the average distribution of homopolymer lengths in all the remaining samples. The assumption is that the majority of samples share the same genotype at any given locus. Hp_caller performs a binomial test of the hypothesis that the sample distribution has a different mode than the locus distribution. If that p-value is below the threshold set for mutation calling, then the sample is called as a mutant. In addition to this calculation, there are a number of other requirements for calling, such as minimum and maximum read depths in the sample and for all samples at the locus, and a minimum locus quality (LQ) score. A more detailed description may be found in the Supplemental Methods section of S1 File.

Unique mutations were identified using custom scripts as described for point mutations; the allele frequency spectrum for homopolymer mutations is shown in Fig C, part B in S1 File. Custom scripts were also used to compare samtools homopolymer calls to hp_caller mutation calls. Correlations with genomic features, transcription, and replication timing were analyzed using bedtools [71] and custom scripts.

### Calculation of mutation rates in homopolymers

The mean mutation frequency is the number of mutations divided by the number of callable loci, and the mean mutation rate is the mutation frequency divided by the number of generations (~1000). The Clopper-Pearson method was used to calculate 95% confidence intervals assuming a binomial distribution. In many cases, there were no events observed at a particular length in a particular construct. For this reason, we typically performed analyses on length windows, such as A:T homopolymers with lengths between 7 and 16 nt.

### Fluctuation analysis

Fluctuation analysis was performed as described, using 10 cultures per strain per experiment [13]. Mutation rates were estimated for each experiment using the maximum likelihood method as implemented in FALCOR [72]. The reported mutation rates and errors are the mean and standard error of mutation rate estimates from at least three independent experiments for each strain. A comparison of mutation rates determined by mutation accumulation and fluctuation analysis experiments is shown in Fig I in S1 File.

### Electrophoretic mobility shift assays

Oligonucleotide sequences were as follows: forward εA oligo, 5’-DyLight647-TAGCATCCT **εA** CCTTCTCTC; forward A oligo, 5’-DyLight647-TAGCATCCT**A**CCTTCTCTC; reverse_T oligo, GAGAGAAGG**T**AGGATGCTA; reverse_b1 5’-GAGAGAAGGAGGATGCTA. The abbreviation **ε**A indicates the nucleotide containing the base 1,*N*^6^-ethenoadenine. Oligonucleotides were ordered from either Glen Research or IDT. All oligonucleotides were gel purified before use; concentrations were determined by absorbance at 260 nm. The following substrates were prepared from the above-listed oligonucleotides: forward_εA:reverse_T; forward_A:reverse_T; forward_A:reverse_b1. Oligonucleotides were mixed at a ratio of 1:1.5 forward:reverse and annealed by slow cooling from 95°C to 4°C in 10 mM MES, 50 mM NaCl, pH 6.5.

All purified AAG proteins were the Δ80 construct lacking the amino-terminal 79 amino acids, and were purified as previously described [41]. The active concentration of enzyme was determined by titration of AAG with tight binding εA-DNA using the EMSA assay. Active AAG (0–1000 nM) was incubated with 200 nM εA:T substrate in binding buffer (25 mM HEPES pH 7.5, 200 mM NaCl, 5% glycerol, 0.2 mg/mL BSA, 1 mM DTT, 1 mM EDTA) at 4°C for one minute, and then immediately loaded onto a 2% agarose gel in 1X TBE running at 3 V/cm in the cold room. After electrophoresis for 60 minutes, gels were scanned on a Typhoon Trio+ imager using the 633 nm laser, the 670BP30 emission filter, and the +3 mm focal plane. Bands were quantitated using ImageQuant TL (GE Healthcare) and the fraction of substrate bound was calculated according to the equation:

$$f_{bound}=\frac{\sum \left(signal\;in\;shifted\;bands\right)}{\sum \left(signal\;in\;all\;bands\right)}$$

Binding affinities for undamaged duplex DNA and undamaged bulged adenines were determined by titrating active enzyme. We fit the fraction of substrate bound to a one-site binding model with a Hill coefficient:

$$y=\frac{x^{h}}{{K_{d}}^{h}+x^{h}}$$

The *K*_d_ determined by this method is an apparent *K*_d_ that reflects nonspecific binding of AAG to the multiple sites on undamaged duplex DNA as well as specific binding to the bulged adenine for the A:b1 substrate. In this application, the Hill coefficients do not have a physical meaning [73]; rather they are correction factors that compensate for the simplistic mathematical model. We observed Hill coefficients between 1.3 and 1.4 for the bulged DNA and between 1.2 and 1.8 for the undamaged DNA with standard errors of ~0.2 for all fits.

## Supporting information

S1 File Supporting text, figures, and tables. This file contains supplementary methods describing hp_caller, supporting Figs A through O, and tables A through D which are referred to in the main text.
(PDF)

S2 File Excel spreadsheet of unique mutations in strain 22561, the E125Q mutator strain. (XLSX)

S3 File Excel spreadsheet of unique mutations in strain 22579, the N169S mutator strain. (XLSX)

S4 File Histograms for homopolymer loci called as having mutations by hp_caller. Each histogram shows the distribution of homopolymer lengths in all reads at the locus (“LOCUS”) and the distribution of homopolymer lengths in reads from samples called as mutants (indicated by sample numbers). The read depth of the locus distribution is normalized to the read depth in the sample with the fewest reads at the locus, which is not necessarily the mutant sample. The read depths for the mutant sample distributions are not normalized. The title for each panel indicates the chromosome and start position of the homopolymer, as well as the hp_caller LQ score for the locus. The two mutant calls at G:C homopolymers have “GC” appended to their titles; all other loci are A:T homopolymers. The legend indicates the sample name and the hp_caller LQ score for mutant samples.
(PDF)
