# Supplementary material for: Mechanisms of glycosylase induced genomic instability
Source: PLoS One. 2017 Mar 23;12(3):e0174041. doi: 10.1371/journal.pone.0174041 (PMC5363859; doi:10.1371/journal.pone.0174041)
Supplement: S1 File — This file contains supplementary methods describing hp_caller, supporting Figs A through O, and tables A through D which are referred to in the main text. (PDF) [file pone.0174041.s001.pdf]

Supporting Information File S1
"Mechanisms of Glycosylase Induced Genomic Instability"
D.E. Eyler, K.A. Burnham, T.E. Wilson, and P.J. O'Brien

Contents

Supplemental Methods (p. S2–4)
Supplemental Discussion (p. S5–6)
Supplemental References (p. S7)
Fig A. Overview of mutation calling pipeline (p. S8)
Fig B. Glycosylase constructs are expressed (p. S9)
Fig C. Allele frequency spectra and unique mutations (p. S10)
Fig D. Mutator strains have distinct point mutation spectra (p. S11)
Fig E. Mutations are evenly distributed across chromosomes and are not clustered (p. S12)
Fig F. No effect of flanking sequence on mutations in the N169S cohort (p. S13)
Fig G. The number of loci able to be called by hp_caller is a function of sequence and length (p. S14)
Fig H. A custom homopolymer caller, hp_caller, outperforms samtools (p. S15)
Fig I. Fluctuation analysis and mutation accumulation experiments give similar estimates of mutation rates in A:T homopolymers. (p. S16)
Fig J. Mutations are not correlated with genomic features (p. S17)
Fig K. The replication times of mutated loci are randomly distributed (p. S18)
Fig L. Transcription is not associated with glycosylase-induced mutations (p. S19)
Fig M. Binding data for all AAG constructs used in the EMSA experiments. (p. S20)
Fig N. Distribution of parameters calculated by hp_caller for callable loci in the yeast genome. (p. S21)
Fig O. Models for glycosylase-induced mutagenesis. (p. S22)
Table A. Parameters used for bowtie2 and samtools mutation calling (p. S23)
Table B. Strains used in this study (p. S24)
Table C. Parameters used for hp_caller mutation calling. (p. S25)
Table D. K_α/K_S ratios for passaged strains. (p. S26)

Supplemental Methods

Mutation calling at homopolymers by hp_caller

We did not achieve the desired level of sensitivity and accuracy for mutations at homopolymers using samtools or other tools. Hence, we developed a set of tools that utilize the characteristics of our experiment to call homopolymers with as much sensitivity and accuracy as possible.

Mutation calling is a two-step process; the first tool is hp_aggregator, which collects the raw data from the bam files, and the second is hp_caller, which uses the raw data to call mutations. Hp_aggregator takes as input a fasta file with the reference genome sequence, a bed file containing the location of the all the homopolymers in the reference genome to be analyzed, and the set of bam alignment files to be analyzed. The program iterates through each homopolymer locus in the genome and identifies the sequences flanking the homopolymer in the reference genome. The overall pattern thus becomes:

(5' flanking sequence, 4 nt)(homopolymer, ≥4 nt)(3' flanking sequence, 4 nt)

By default, the flanking sequences are four nucleotides long. Artifacts may occur in two particular cases: at the ends of interrupted homopolymer sequences where a single incorrect base call can fuse together two adjacent homopolymers, and at loci where the overall pattern matches to the reference genome more than once in the vicinity of the targeted homopolymer. In these cases, hp_aggregator extends the length of the flanking sequences until the overall pattern matches unambiguously to the reference sequence. Hp_aggregator generates a text file containing the lengths of every 5' and 3' pattern sequence used (pattern_length_report.txt). In our application, more than 90% of flanking sequences were 6 nt or shorter. Input reads mapping to the targeted homopolymer are retrieved from the bam alignment file, and the flanking sequences are used to identify the ends of the homopolymer in the input reads and determine the length of the homopolymer. Hp_aggregator sums the number of reads with each observed homopolymer length and stores the data in the output file.

Mutation calling is performed by hp_caller, which takes the data file from hp_aggregator as its input. At each locus, hp_caller performs a number of checks to determine if the locus as a whole can be called. First hp_caller checks to see that the total read depth at the locus is between the lower and upper limits for read depth. Loci with very low coverage are likely to generate artifacts even if they are called, while loci with unusually high coverage are probably repetitive sequences; reads at these loci may not have been mapped to the correct repetitive element and thus their homopolymer length distributions are not informative. If the locus fails the locus depth check, it is reported as uncallable (all samples have genotype N/N). Next, a locus quality score is calculated for the locus distribution according to the formula:

locus quality = 100 * ((reads at mode-reads at next most abundant length))/(total reads)

Thus the locus quality score is the difference between the percentage of data supporting the modal length and the percentage of data supporting the next most likely

length. We examined the distribution of locus quality scores for all loci in the genome and set a minimum locus quality score threshold of 10 for calling (Fig N, this file).

Lastly, hp_caller examines each sample to determine the number of uncallable samples at the locus. Uncallable samples meet one or more of the following criteria: sample depth below threshold, sample depth above threshold, homopolymer length distribution not unimodal, or sample quality score below threshold. If there are too many uncallable samples at a locus, the locus is reported as uncallable.

If the locus is callable, then hp_caller analyzes each sample. Hp_caller subtracts the sample distribution from the locus distribution, and performs a binomial test of the hypothesis that sample and locus distributions have different modes. The null hypothesis is that the sample and locus distributions have the same mode. If there is less than a $1x10^{-5}$ chance that the modes of the two distributions are the same, the null hypothesis is rejected. The distribution of mutant p-value scores is shown in Fig N (this file).

Calculation of mutation rates in homopolymers

Mean mutation rates were determined by dividing the number of observed mutations by the number of homopolymer replication events. However, it is not appropriate to use the total number of homopolymers of a given length in the genome, because not all homopolymer loci could be called, especially at longer lengths (see Fig A7). Instead, this parameter was determined by counting the number of called homopolymer loci in the vcf file produced by hp_caller. Callable homopolymer loci were those at which the locus could be called as a whole, and no more than 3 samples remained uncalled. Thus, the total number of trials for homopolymers of length 8 in one AAG construct was (8 strains) x (2560 callable AT homopolymers of length 8) = 20,480. The mean mutation frequency is the number of mutations divided by the number of callable loci, and the mean mutation rate is the mutation frequency divided by the number of generations (~1000). We calculated 95% confidence intervals using the Clopper-Pearson method. In many cases, there were no events observed at a particular length in a particular construct. For this reason, we typically performed analysis on length windows, such as A:T homopolymers with lengths between 7 and 16 nt.

Calculation of K_a/K_s

Open reading frames (ORFs) were extracted from annotated features in the SacCer3 genome [1] and filtered to remove ORFs annotated as dubious. Unique mutations in each strain overlapping ORFs were extracted via bedtools [2]. The number of synonymous sites (S_{sites}) is the sum of all possible synonymous substitutions that could have been observed at all of the loci with point mutations. The number of nonsynonymous sites (N_{sites}) is the corresponding sum of all possible nonsynonymous substitutions. The following sums are used to calculate the number of observed synonymous substitutions (S_{obs}) and observed nonsynonymous substitutions (N_{obs}).

$$S_{obs} = \sum observed\ synonymous\ substitutions$$

$$N_{obs} = \sum observed\ nonsynonymous\ substitutions$$

The K_α/K_S ratio is then given by the following formula:

$$\frac{K_{\boldsymbol{\alpha}}}{K_S} = \frac{N_{obs}/N_{sites}}{S_{obs}/S_{sites}}$$

Verification of AAG expression in yeast

AAG expression was induced by shifting log-phase cultures from CSM-uracil media with 2% glucose to media with 2% galactose and incubating for 16 hours. Lysates were prepared by bead beating and the protein concentration determined by Bradford assay. Aliquots of lysate were prepared at a concentration of 200 mg/L in SDS-PAGE loading buffer. Relative lysate concentrations were determined by Coomassie staining of three independent 15% SDS-PAGE gels. For western blotting, samples were separated on 15% SDS-PAGE gel and transferred to Immobilon-FL membranes using wet transfer at 70 V for 70 minutes. Membranes were blocked with Odyssey PBS Blocking Buffer (Licor #927-40000) for one hour at room temperature. Blots were incubated with anti-human MPG (Sigma HPA006531, lot #A96602) at a 1:100 dilution in Odyssey blocking buffer overnight at 4°C. After three washes with 1x PBS with 0.1% Tween-20, blots were incubated with a Dylight 680-conjugated sheep anti-rabbit secondary antibody (Rockland Immunochemicals #611-644-122 lot #22076) for one hour at room temperature in 1x PBS with 0.1% Tween-20 and 0.01% SDS. Blots were washed three times with 1x PBST and scanned on a Licor Odyssey CLX scanner. Varying amounts of lysate from the wild-type strain were loaded and quantified to demonstrate that the fluorescent signal was linearly proportional to the quantity of protein loaded. The amount of AAG in each sample was quantified relative to the wild-type sample on three independent blots. These values were corrected for variations in loading using Li-Cor Revert total protein stain according to manufacturer instructions.

Supplemental Discussion

Limitations of hp_caller

Hp_caller makes a number of assumptions that are crucial to its proper function. These are listed here in order of their importance.

- most samples have the same genotype

hp_caller works by comparing individual sample distributions to the average distribution in all the other samples (the locus distribution). This works fine as long as the locus distribution predominantly represents the distribution that comes from one genotype. If the locus average distribution represents multiple genotypes, hp_caller will compute a low probability of a mutation for all genotypes that are included in the locus genotype.

- at least 5 samples, but preferably more

hp_caller compares the distribution in one sample to the average distribution in all the other samples. Assuming that there are 5 samples and that one of them is a variant, when each of the four wild-type samples are being considered, the locus distribution will have a 25% contribution from the variant sample. This will affect the mutant probabilities that are calculated by hp_caller. In our application, hp_caller was able to discriminate between variants and wild-type samples when as many as 8 out of the 47 (or 1/6th) of the samples were variant.

- equivalent read depth in all samples

For the same reasons that hp_caller performs better with more samples, it is also important that samples have approximately equal coverage. If one sample contributes the majority of the reads to the locus distribution, the locus distribution will be representative of that one sample rather than representative of the average genotype at the locus.

- sufficient read depth

hp_caller requires sufficient read depth both at the locus as a whole and in individual samples. Confidence in the locus genotype and in sample genotypes increases with increasing read depth. At a minimum, individual samples require 5 reads, though samples with such low depth may fail to meet other thresholds for calling. We have not determined the absolute minimum for locus depth; in theory, 50 reads should be sufficient to make a high confidence call. In our experiments we used a minimum locus depth of 200 reads, however, since this would yield an average per sample depth below 5 reads, the locus minimum depth was effectively not used in our data set.

Extensibility of hp_caller to other microsatellite sequences and diploids

The approach used in hp_caller is extensible to microsatellite sequences generally. We successfully applied it to the detection of indels in dinucleotide microsatellites in our data set, although too few events were observed for analysis (data not shown). The algorithm in hp_caller is the same, but modifications were required to the aggregator script to allow it to determine dinucleotide repeat lengths. The dinucleotide aggregator script will be made available on request.

In theory, diploid variants could be called using a variation on the algorithm in hp_caller, subject to a similar set of constraints (most samples have the same genotype, individual samples have low numbers of mutations, etc.). Rather than using a binomial probability and classifying sample homopolymer read lengths as "same" or

“different” than the locus, one would calculate a multinomial probability that the observed sample distribution was the same as the locus distribution. The absolute value of this probability will vary with read depth in the sample, so the algorithm would need a method of normalizing probability values for read depth. In a previous version of hp_caller that used a multinomial probability, this was done using an odds ratio. In addition to calculating the probability of obtaining the observed sample distribution, the algorithm also calculated the absolute probability of obtaining the most likely sample distribution (assuming that the sample distribution and the locus distribution were the same). The logarithm of the ratio of these two probabilities was reported as a log odds ratio. The log odds ratio (LOR) was then used to set a threshold for calling a sample as containing a variant allele.

Having identified a sample distribution as being unlikely to have arisen from the locus distribution, the task of calling the sample genotype remains. Calling homopolymer genotypes is simple in haploids as it is just the mode of the distribution. In diploids it is a little trickier to do without making assumptions about what the starting (or locus) genotype is. Without having tested the following method, it is difficult to say how well it would work or what mechanisms for error avoidance would need to be built in. Nonetheless I would propose the following method.

The locus probability distribution was computed earlier. Now a probability distribution is computed for the variant sample. This is just the number of reads at a given homopolymer length normalized to the total number of homopolymer spanning reads in the sample. I would then compute a difference distribution by subtracting the locus probability distribution from the sample probability distribution. The global maximum of the difference distribution will indicate an allele that is present in the sample at greater copy number than it is in the locus distribution; i.e., an allele that the sample has “gained” relative to the locus genotype. Correspondingly, if the sample has gained an allele, it should have also lost one; and the global minimum of the difference distribution will indicate the homopolymer length of the allele that has been lost.

This method allows identification of the variant allele in the variant sample, and classification of the mutation as an insertion or deletion, without making assumptions about whether the locus distribution arose from a homozygous or heterozygous genotype.

Supplemental References

1. SGD Project. saccharomyces_cerevisiae.gff [Internet]. [cited 15 Jul 2014]. Available: http://downloads.yeastgenome.org/curation/chromosomal_feature/saccharomyces_cerevisiae.gff
2. Quinlan AR, Hall IM. BEDTools: A flexible suite of utilities for comparing genomic features. Bioinformatics. 2010;26: 841–842. PubMed PMID:20110278. doi:10.1093/bioinformatics/btq033
3. Pelechano V, Wei W, Steinmetz LM. Extensive transcriptional heterogeneity revealed by isoform profiling. Nature. 2013;497: 127–131. doi:10.1038/nature12121

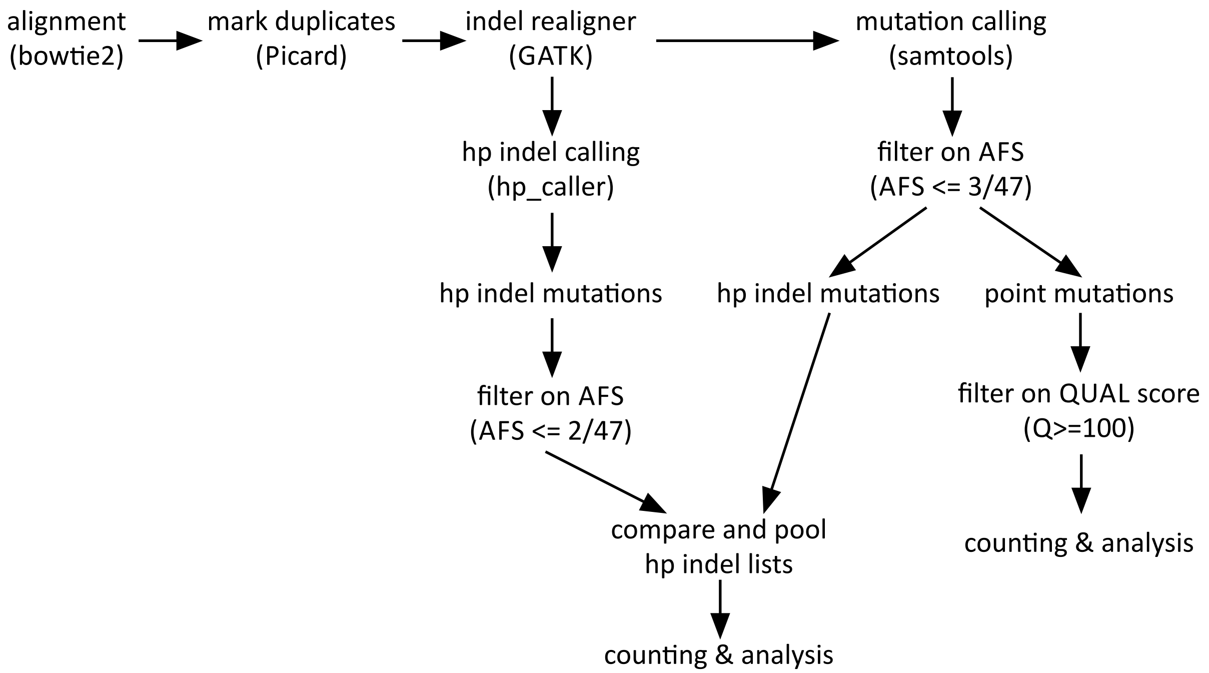

Fig A. Overview of mutation calling pipeline.

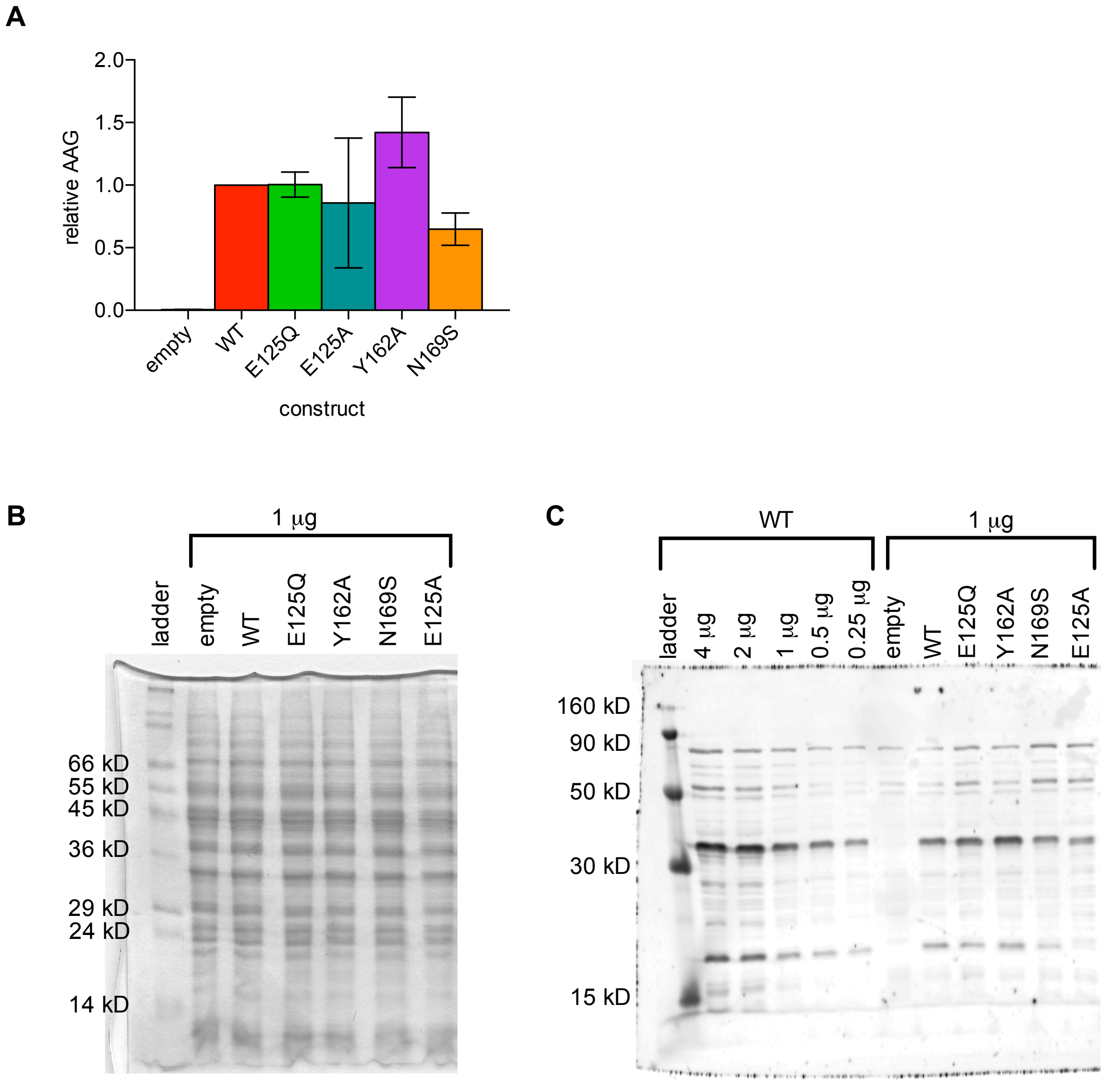

Fig B. All pYES2-AAG constructs are expressed in yeast. (A) AAG expression levels were determined by fluorescent western blotting, corrected for lysate concentration, and normalized to expression of wild type AAG. Three blots were used to quantitate relative expression levels and three gels were used to quantify relative lysate concentration. (B) Representative Coomassie stained 15% SDS-PAGE gel of 1 μg of lysate from strains expressing each construct. (C) Representative fluorescent western blot of AAG constructs. The dilution series of wild type lysate on the left half of the gel was quantitated to verify that the fluorescent signals observed were within the linear range of the instrument.

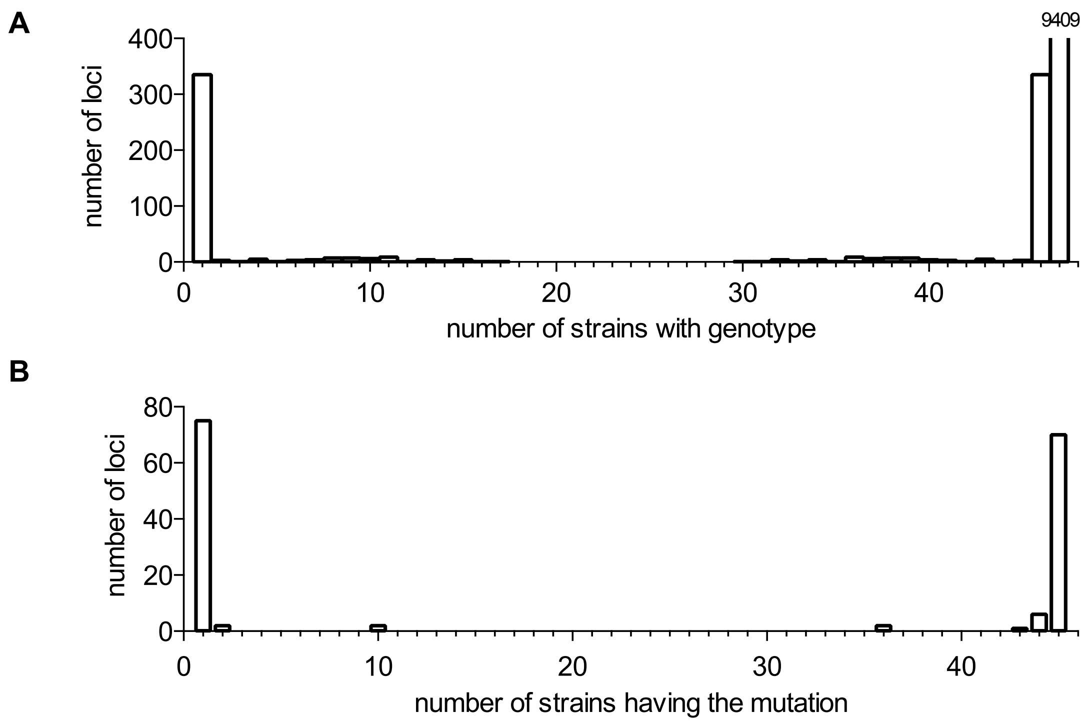

Fig C. Allele frequency spectra and unique mutations. Filtering for unique mutations that occurred during passaging was an important part of our pipeline. (A) Allele frequency spectrum for all samtools point mutation calls. The large peak at AFS=47 is due to differences between the genome sequence of our strain and the reference genome sequence. (B) Allele frequency spectrum for mutations called in homopolymers by hp_caller. The total number of strains (x-axis) is lower in (B) because the pseudodiploid strain was excluded from calling. This histogram also does not include loci where all 46 strains differed from the reference sequence and thus there is no large peak at AFS=46.

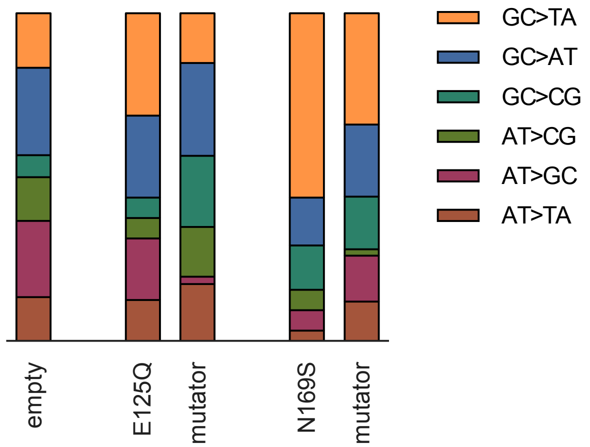

Fig D. Mutator strains have distinct point mutation spectra. The strains in the E125Q and N169S cohorts that have acquired point mutator phenotypes demonstrate distinct point mutation spectra relative to the other strains in their cohorts. The fraction of point mutations in each category are shown for the empty vector cohort, the E125Q cohort, the E125Q mutator strain, the N169S cohort, and the N169S mutator strain.

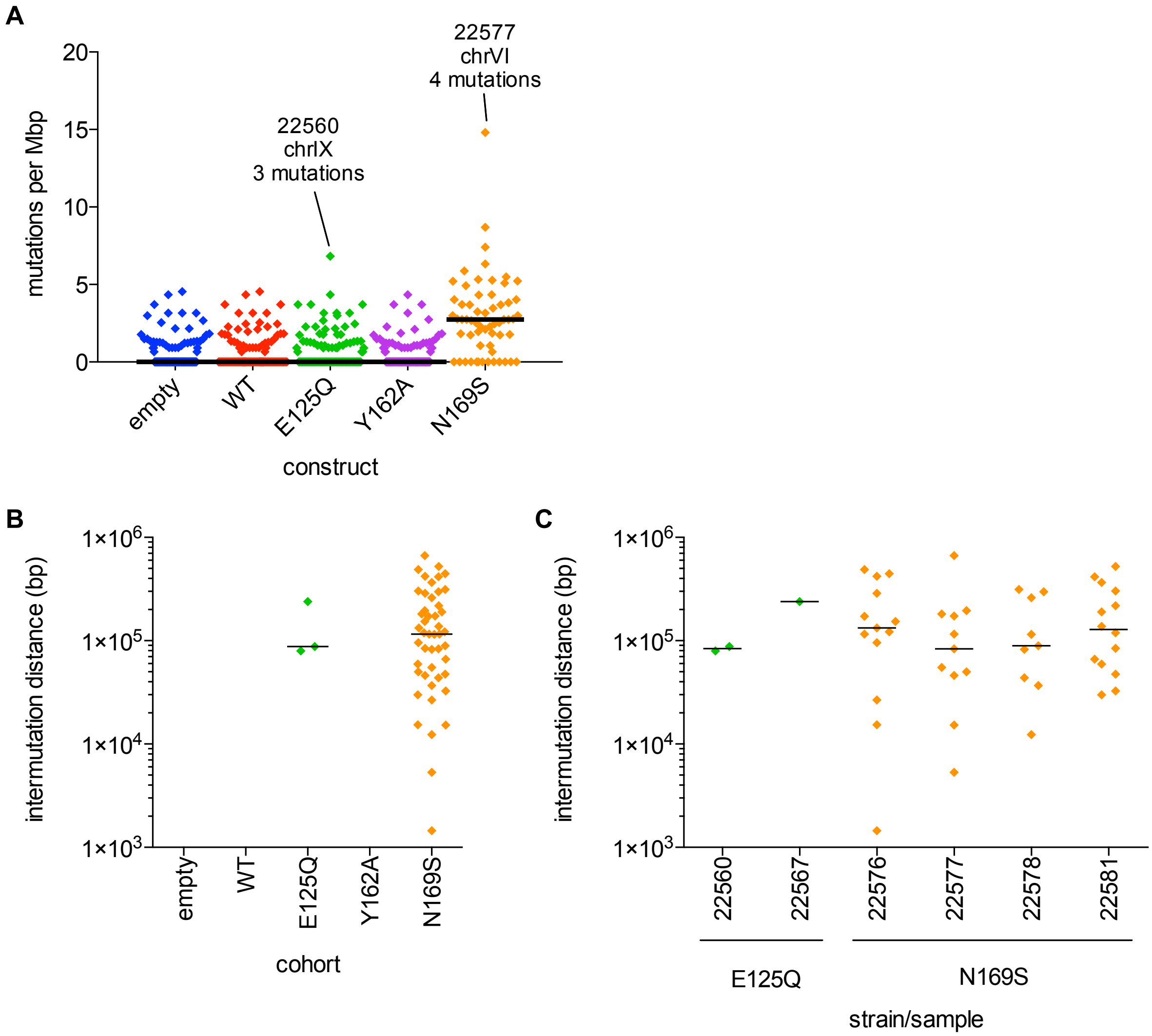

Fig E. Mutations are evenly distributed across chromosomes and are not clustered. (A) The density of all mutations (point and homopolymer indel mutations) is plotted for each chromosome in each strain. Points indicating zero mutations per Mbp indicate chromosomes with no mutations. The outstanding points are labeled with their strain number (see Table B in this file), chromosome, and number of mutations. Note that chromosomes VI and IX are in the bottom quartile of chromosome size in yeast and that even in the outstanding cases, the number of mutations is still quite low. (B) The distance between adjacent mutations in a single strain (y-axis, bp) organized by cohort. The empty, WT, and Y162A cohorts have no data points because no two mutations were on the same chromosome in the same strain. (C) The distance between adjacent mutations for all strains having at least one pair of mutations on the same chromosome. Note that all pairs of mutations are at least 1000 bp apart and in most cases much farther. Horizontal lines in (A), (B), and (C) indicate the medians of each distribution.

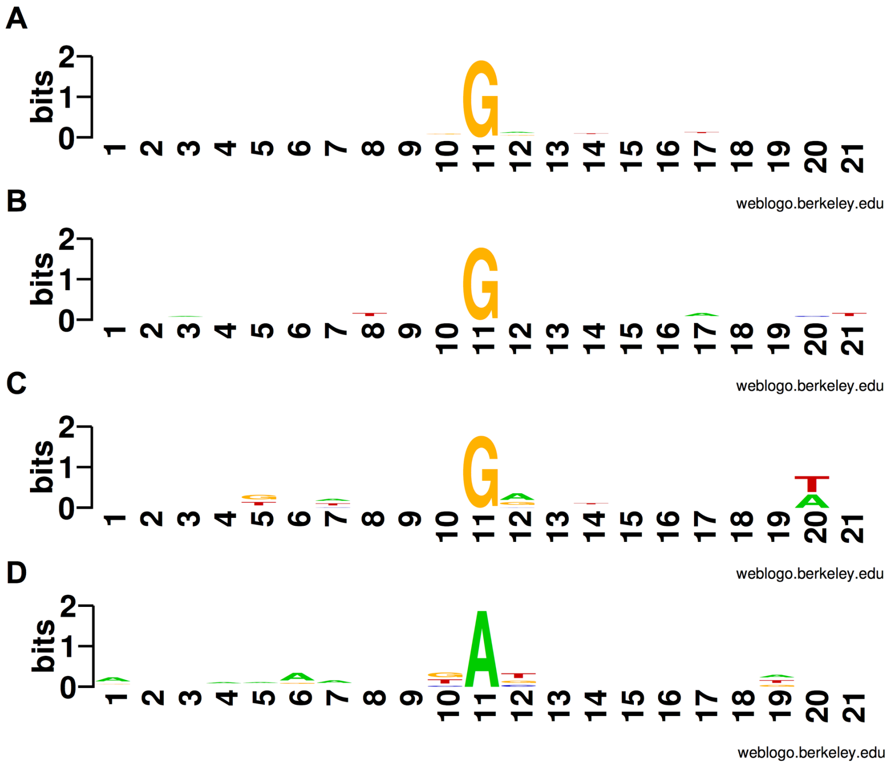

Fig F. No effect of flanking sequence on mutations in the N169S cohort. (A) Sequence logo for G>T point mutations. (B) Sequence logo for G>A point mutations. (C) Sequence logo for G>C point mutations. (D) Sequence logo for indels in A:T homopolymers. Homopolymers of varying lengths were condensed to a single “A” in the center of the sequence in order to examine the surrounding sequences.

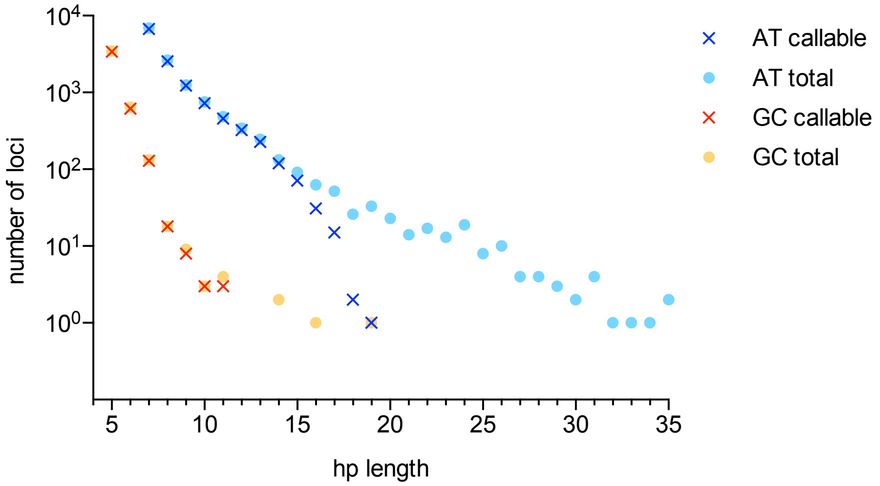

Fig G. The number of loci able to be called by hp_caller is a function of sequence and length. hp_caller uses a number of factors to determine if a locus is callable (see Supplemental Methods). The dominant determinant is the number of high-quality reads spanning the homopolymer, which is itself a function of sequencing depth (~50x in our libraries). GC homopolymers were able to be called at lengths up to 10 nt, while AT homopolymers were able to be called efficiently at lengths up to 16 nt.

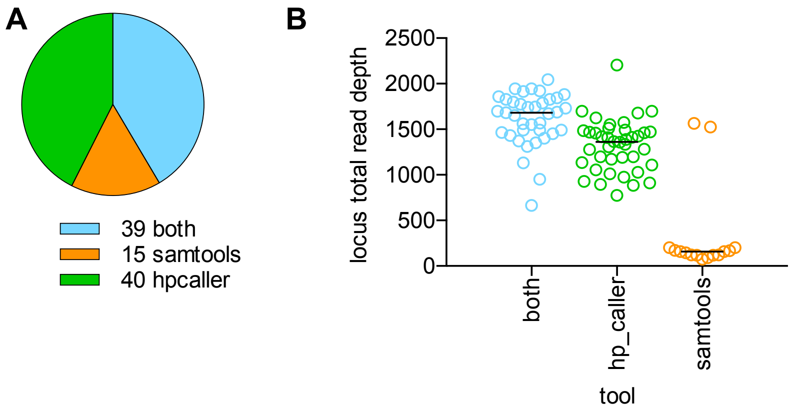

Fig H. A custom homopolymer caller hp_caller outperforms samtools. (A) Pie chart comparing the number A:T homopolymer indel calls made by samtools, by hp_caller, or by both tools. Note that indel calls in all strains were used for this comparison, including the mutator strains excluded from other analyses. (B) The total read depth at homopolymer loci (i.e., the total number of reads in all samples) for homopolymer loci called as having mutations by hp_caller, samtools, or both. Lines indicate the medians of each distribution. Note that the calls made by samtools only are mostly at loci with very low coverage, <5x. The two valid samtools calls were missed by hp_caller because of strain-background point mutations in the sequences flanking the homopolymers. These two mutations were included in analyses of homopolymer mutation rates.

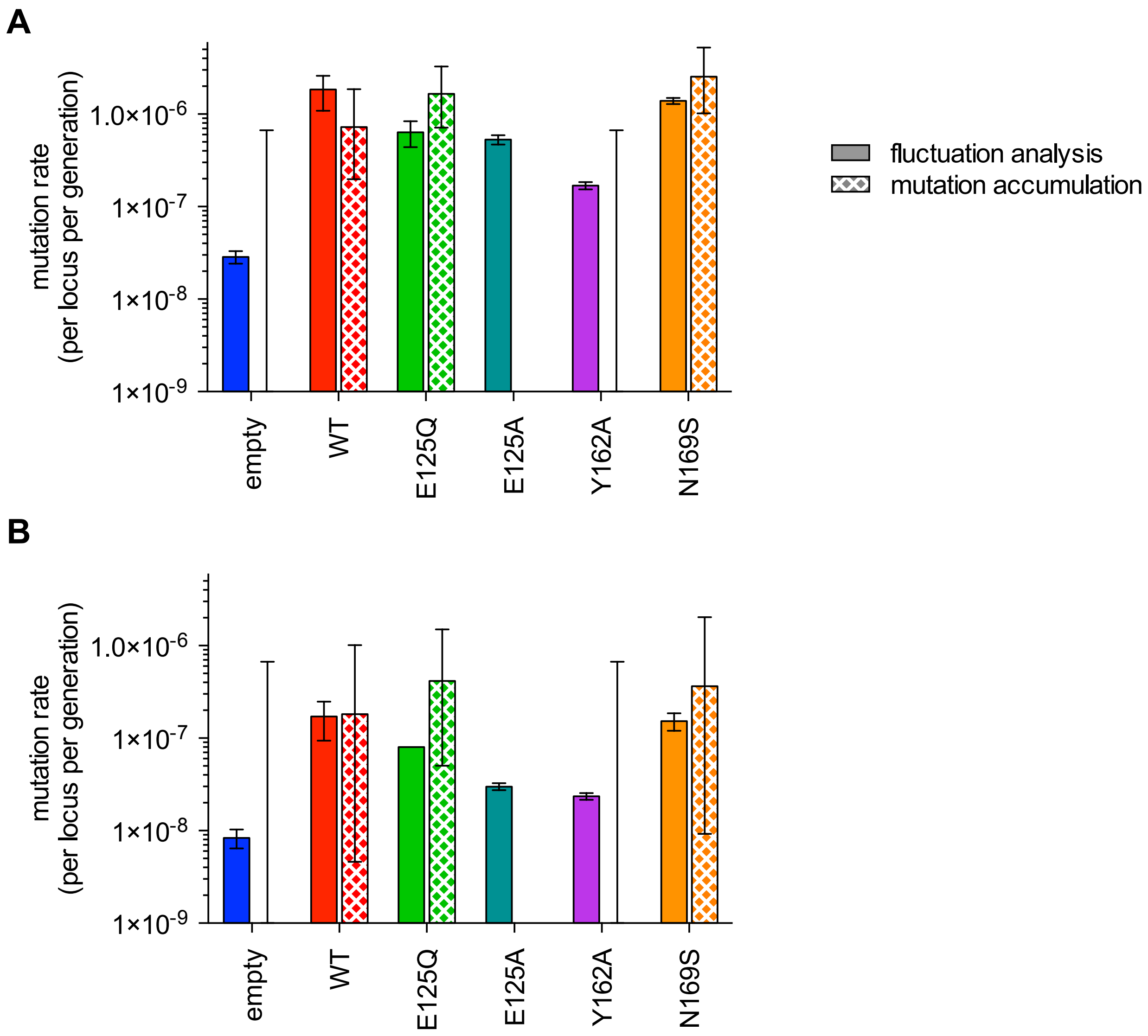

Fig I. Fluctuation analysis and mutation accumulation experiments give similar estimates of mutation rates in A:T homopolymers. Mutation rates were determined by fluctuation analysis (solid bars) using (A) the LYS2-A14 -1 frameshift reporter allele or (B) the LYS2-A12 +1 frameshift reporter allele. Mutation rates from the mutation accumulation experiment were calculated using mutations in all A:T homopolymers with lengths between 12 and 14 nt, since there were not enough events to permit calculation of mutation rates at a single homopolymer length. Error bars are SEM for fluctuation analysis experiments and asymmetric 95% confidence intervals for mutation accumulation experiments.

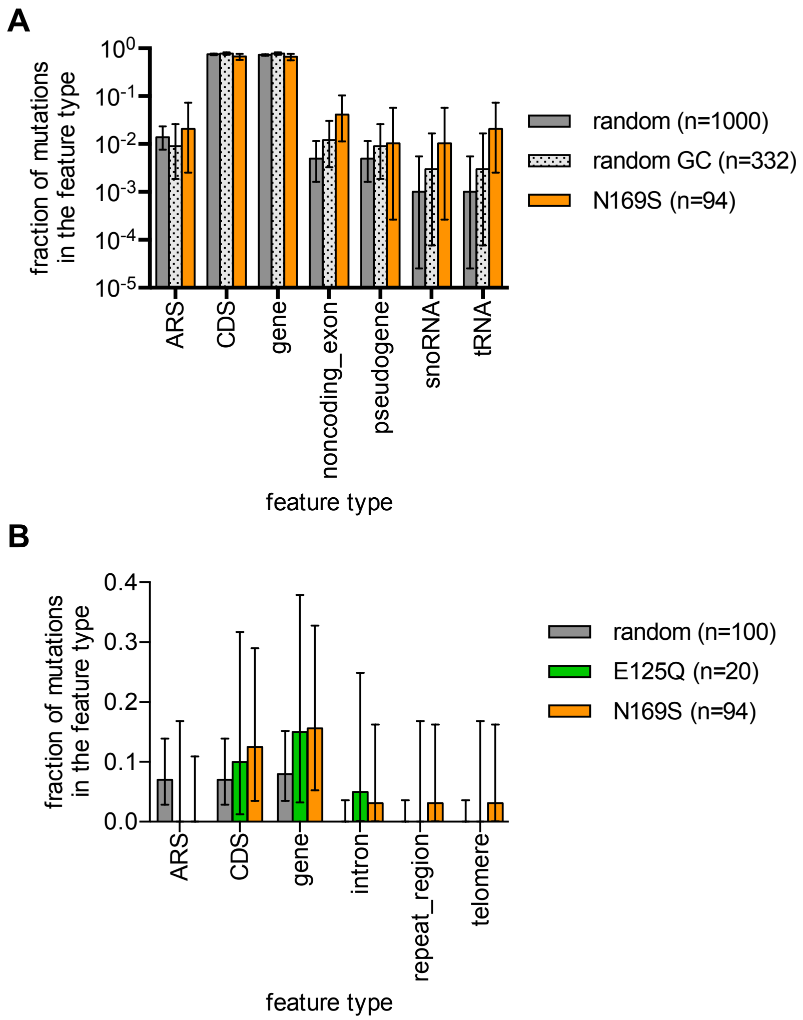

Fig J. Mutations are not correlated with genomic features. The fraction of mutations (y-axis) located within a given genomic feature is shown for selected feature types (x-axis). Feature types without mutations in any data set are not shown for clarity. Error bars are 95% confidence intervals. (A) Point mutations in the N169S cohort are not correlated with annotated features. The N169S cohort mutations are shown in orange. Solid gray bars are a set of 1,000 computer-generated random mutations, while the stippled gray bars are the set of 332 GC base pairs within the full random set (since the N169S mutant acts primarily at GC base pairs, and the distribution of GC base pairs in the yeast genome is non-random). (B) Mutations in A:T homopolymers are not associated with genomic features. Only callable AT homopolymers with lengths between 10 and 16 nt were included in this analysis, since longer homopolymers have a higher mutation rate and distinct distribution relative to shorter homopolymers. A set of 100 computer-generated random mutations is shown by gray bars, while green bars indicate the E125Q cohort and the orange bars show results for the N169S cohort.

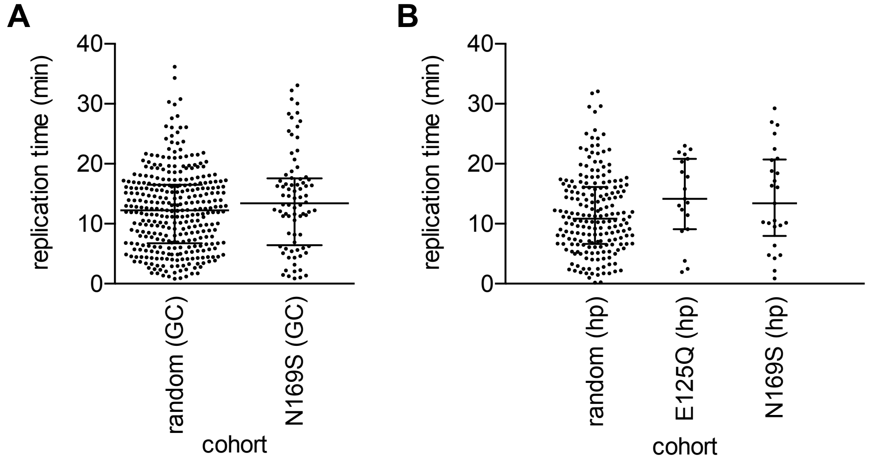

Fig K. The replication times of mutated loci are randomly distributed. (A) Replication times for mutated GC base pairs in the N169S cohort (n=80) are compared to a set of randomly chosen GC base pairs (n=330). The line indicates the median and error bars are the interquartile range. (B) Replication times for mutated AT homopolymers with lengths between 10 and 16 nt. There is no significant difference between experimental cohorts and the randomly-generated cohort (Mann-Whitney U-test). Lines indicate the median and error bars are the interquartile range.

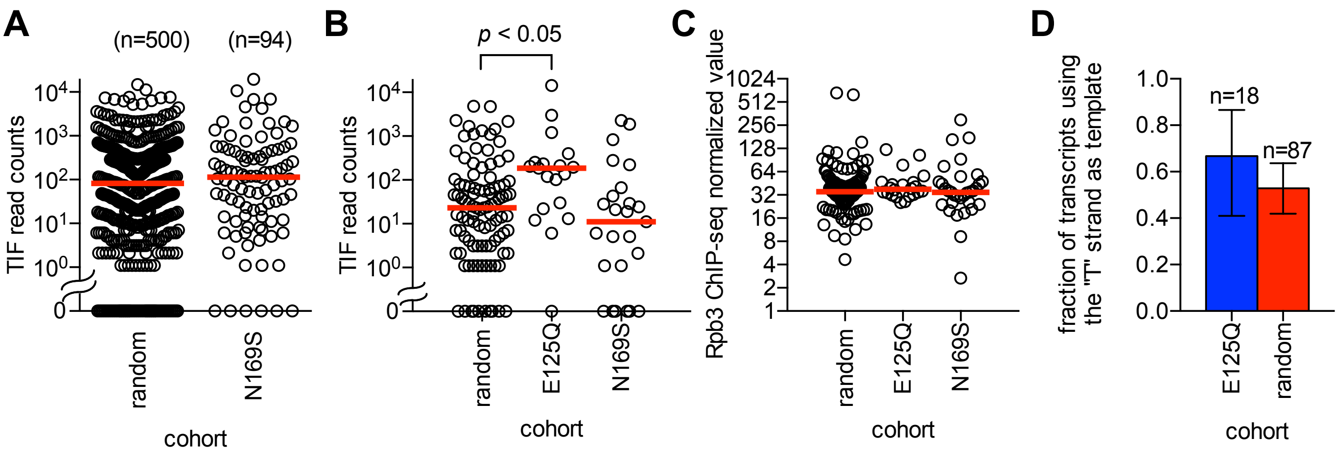

Fig L. Transcription is not associated with glycosylase-induced mutations. (A and B) Transcriptional isoform (TIF) read counts were used as a measure of steady state mRNA levels since the TIF-seq data set was obtained from yeast grown in galactose media, as our strains were. The TIF-seq method detects transcriptional isoforms via high throughput sequencing of uniquely barcoded and circularized cDNAs [3]. (A) TIF read counts for a set of computer-generated random point mutations and observed N169S cohort point mutations are shown on the y-axis. Lines indicate the median of each distribution. The medians are not significantly different by the Mann-Whitney U-test. (B) TIF counts for randomly chosen and mutated homopolymers in the E125Q and N169S cohorts. The E125Q cohort had an increased median transcription level relative to the random set of loci (Kruskal-Wallis test with Dunn's correction for multiple comparisons). (C) To validate the observation in (B) we analyzed occupancy of the RNA polymerase II subunit Rpb3 at the same set of loci in yeast grown in glucose media. No significant effect was observed. (D) No correlation between homopolymer sequence and the transcribed strand was observed in the E125Q or random cohorts.

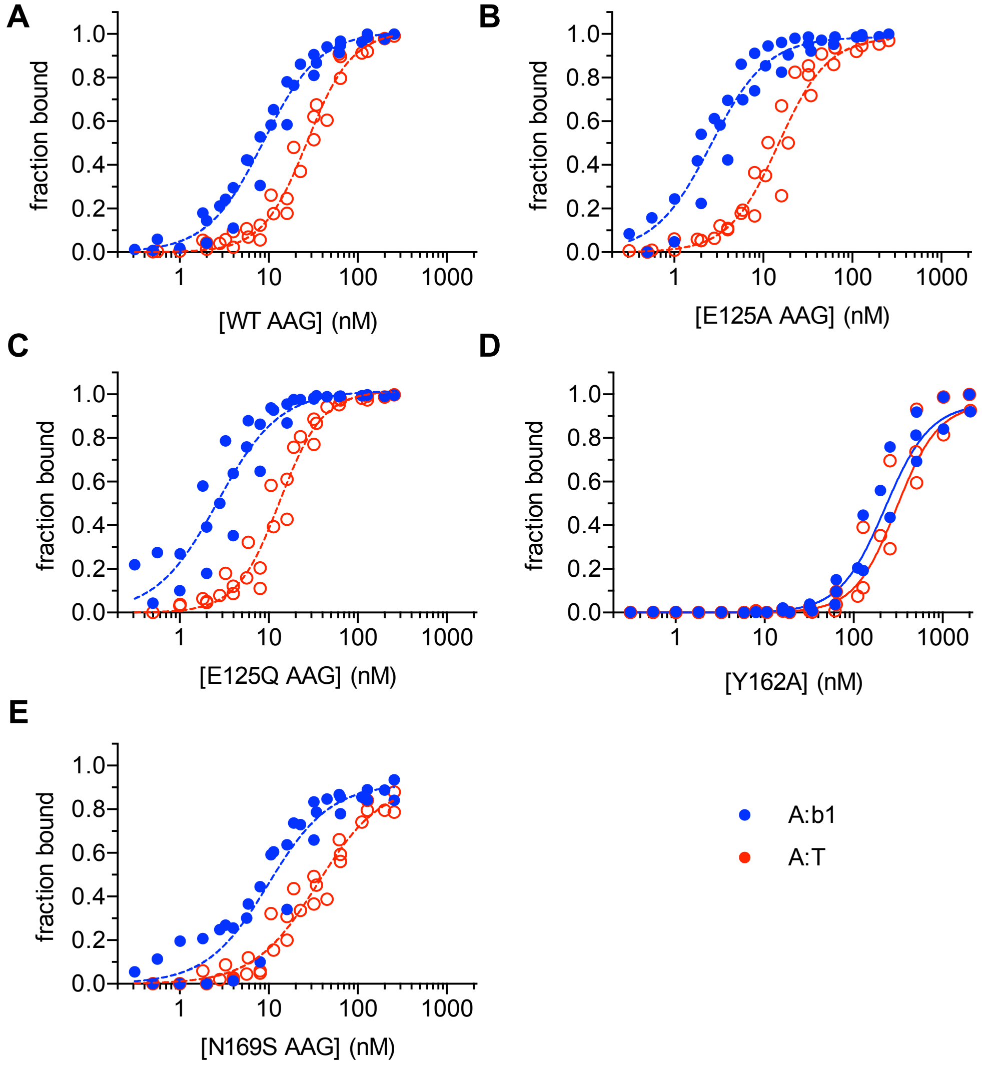

Fig M. Binding data for all AAG constructs used in the EMSA experiments. The fraction of substrate DNA bound is shown as a function of the concentration of active AAG added. The substrate with a bulged A is shown in blue closed circles while the fully duplex substrate is shown in open red circles. A single globally-fit curve is shown for clarity. A) WT AAG B) E125A AAG C) E125Q AAG D) Y162A AAG E) N169S AAG.

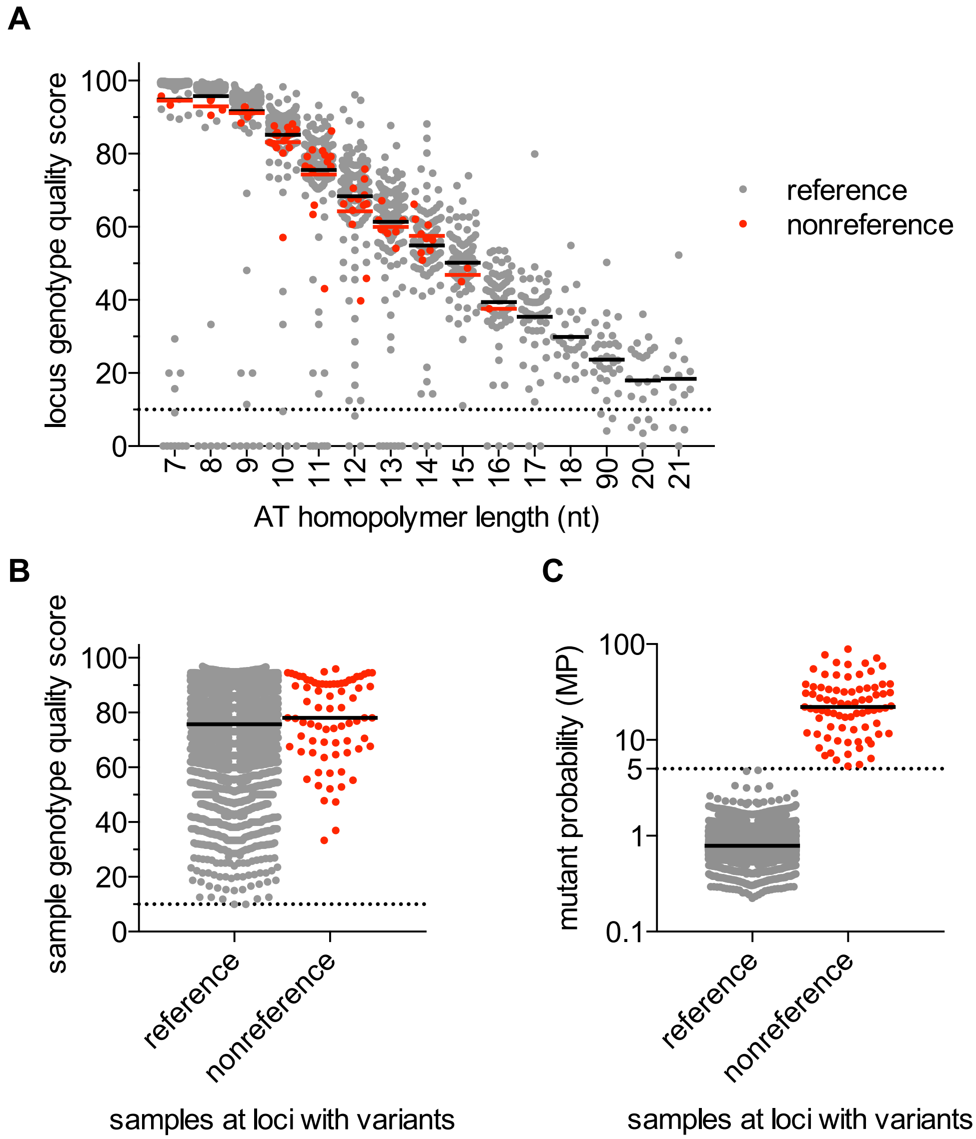

Fig N. Distribution of parameters calculated by hp_caller for callable loci in the yeast genome. A) The distribution of locus genotype quality scores (reported in the QUAL field of the VCF file) for loci with only reference genotype samples, or for loci with a nonreference sample. Scores are shown separately for homopolymers of different lengths since distributions become wider as homopolymer length increases. Loci with nonreference samples have slightly lower quality scores because the nonreference sample influences the calculation. B) The distribution of sample genotype quality scores for samples at loci where one sample was called as non-reference. C) The distribution of mutant probabilities for samples called as reference and nonreference at loci with at least one nonreference sample. For A, B, and C, solid lines indicate the median of each distribution, while dashed lines indicate the threshold values used by hp_caller to determine whether a locus or sample could be called.

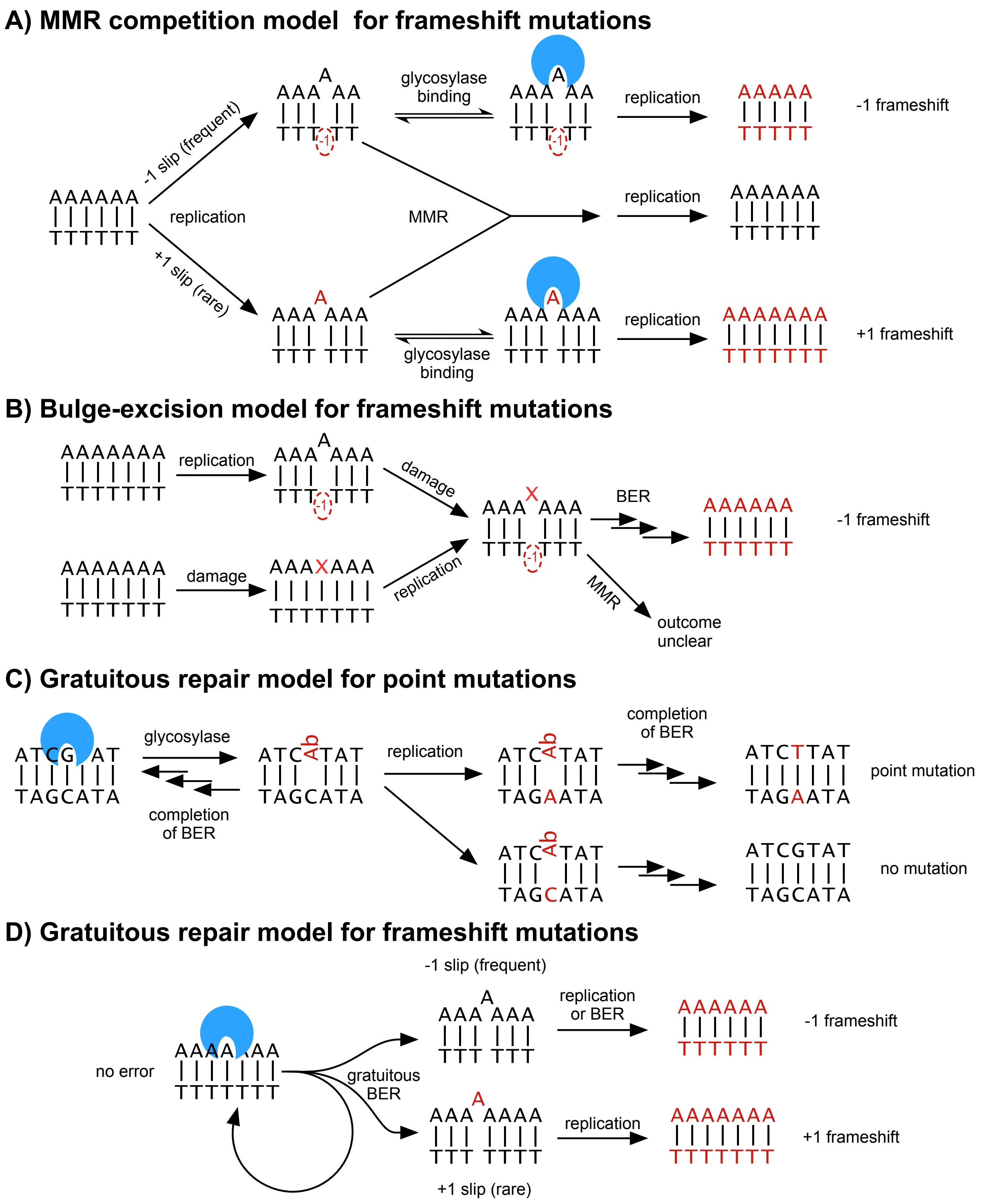

Fig O. Models for glycosylase-induced mutagenesis. This Fig shows additional pathways and intermediates. Note that equivalent models can be shown for replication of A:T homopolymer that results in formation of a bulge T (A,B,D). We also do not show the +1 slip event in panel B.

Table A. Parameters used for bowtie2 and samtools mutation calling.

command	parameter	meaning
bowtie2 v2.1.0	-q	reads are in fastq format
	--phred33	phred encoding of fastq file
	-X 750	maximum insert size 750 bp
	--very-sensitive	sets a number of alignment parameters
samtools mpileup	-g	BCF output
	-p	-m and –F are applied per-sample
	-D	output per-sample DP
	-S	output per-sample strand bias P-value
	-q 30	skip alignments with mapQ < 30
	-ff 1280	skip reads marked as duplicates (1024) or as non-unique (256)
	--rf 2	require alignments to have the paired flag (2)
	-F 0.05	fraction of gapped reads for indel candidates
	-m 5	minimum gapped reads for indel candidates
bcftools view	-bvcg	output only variant sites in bcf format
	-d 0.8	skip loci where less than 80% of samples have coverage
	-s FILE	FILE has lines with “sample_name 1”; sets haploid calling

Table B. Strains used in this study.

strain	construct	number	note
E134	pYES2	22537	LYS-; has 14-nt A run in the LYS2 reporter allele
n/a	pYES2	22538	LYS+; has 13-nt A run in the LYS2 reporter allele
E133	pYES2	22539	starting strain; has 12-nt run in the LYS2 reporter allele
E134	pYES2-AAG-WT	22540	starting strain; 14-nt A run in the LYS2 reporter allele
	pYES2-AAG-E125Q	22541	starting strain; 14-nt A run in the LYS2 reporter allele
	pYES2-AAG-Y162A	22542	starting strain; 14-nt A run in the LYS2 reporter allele
	pYES2-AAG-N169S	22543	starting strain; 14-nt A run in the LYS2 reporter allele
E133	pYES2	22544	used for end strain fluctuation analysis
		22545	
		22546	
		22547	
		22548	
		22549	
		22550	
		22551	
E134	pYES2-AAG-WT	22552	used for end strain fluctuation analysis
		22553	
		22554	
		22555	
		22556	
		22557	
		22558	
		22559	
	pYES2-AAG-E125Q	22560	
		22561	independent mutator phenotype; excluded
		22562	
		22563	
		22564	used for end strain fluctuation analysis
		22565	
		22566	
		22567	
	pYES2-AAG-Y162A	22568	
		22569	
		22570	
		22571	
		22572	
		22573	
		22574	
		22575	
	pYES2-AAG-N169S	22576	used for end strain fluctuation analysis
		22577	used for end strain fluctuation analysis
		22578	used for end strain fluctuation analysis
		22579	independent mutator phenotype; excluded
		22580	nonsense codon in AAG (E268X, GAG>TAG); excluded
		22581	
		22582	pseudodiploid; excluded
		22583	inactivating mutation in AAG (R182G, CGA>GGA);

Table C. Parameters values used in hp_caller.

parameter	value	meaning
--min-sdp	5	minimum sample depth required to make a call
--max-sdp	150	maximum sample depth allowed for making a call
--nsr	1	net supporting reads
--uncallable	3	maximum number of uncallable samples before declaring the locus uncallable
--loc-min	200	minimum locus depth required for calling the locus
--loc-max	2500	maximum locus depth permitted for calling the locus
--mp	5	PHRED scaled probability that the sample distribution has the same mode as the locus distribution.
--asymmetry	0.2	Maximum fractional asymmetry around the mode in a distribution. This is used to filter out loci where the entire locus has a skewed distribution.
--variants_only	0	output data for all loci
--minSGQ	10	minimum sample distribution quality required for calling
--minLQ	10	minimum locus distribution quality required for calling

Table D. K_α/K_S ratios for point mutations in the passaged strains.

	empty	WT	E125Q	Y162A	N169S
total nucleotide substitutions in ORFs	21	14	24	19	60
synonymous nucleotide substitutions	5	7	5	5	16
nonsynonymous nucleotide substitutions	16	7	19	14	44
total nonsynonymous sites	50	22	60	39	134
total synonymous sites	13	20	12	18	46
total number of sites	63	42	72	57	180
K_α/K_S ± SEM	0.83 ± 0.42	0.91 ± 0.48	0.76 ± 0.38	1.29 ± 0.67	0.94 ± 0.27
