# Supplementary material for: Mechanisms of glycosylase induced genomic instability
Source: PLoS One. 2017 Mar 23;12(3):e0174041. doi: 10.1371/journal.pone.0174041 (PMC5363859; doi:10.1371/journal.pone.0174041)
Supplement: S4 File — Each histogram shows the distribution of homopolymer lengths in all reads at the locus (“LOCUS”) and the distribution of homopolymer lengths in reads from samples called as mutants (indicated by sample numbers). The read depth of the locus distribution is normalized to the read depth in the sample with the fewest reads at the locus, which is not necessarily the mutant sample. The read depths for the mutant sample distributions are not normalized. The title for each panel indicates the chromosome and start position of the homopolymer, as well as the hp_caller LQ score for the locus. The two mutant calls at G:C homopolymers have “GC” appended to their titles; all other loci are A:T homopolymers. The legend indicates the sample name and the hp_caller LQ score for mutant samples. (PDF) [file pone.0174041.s004.pdf]

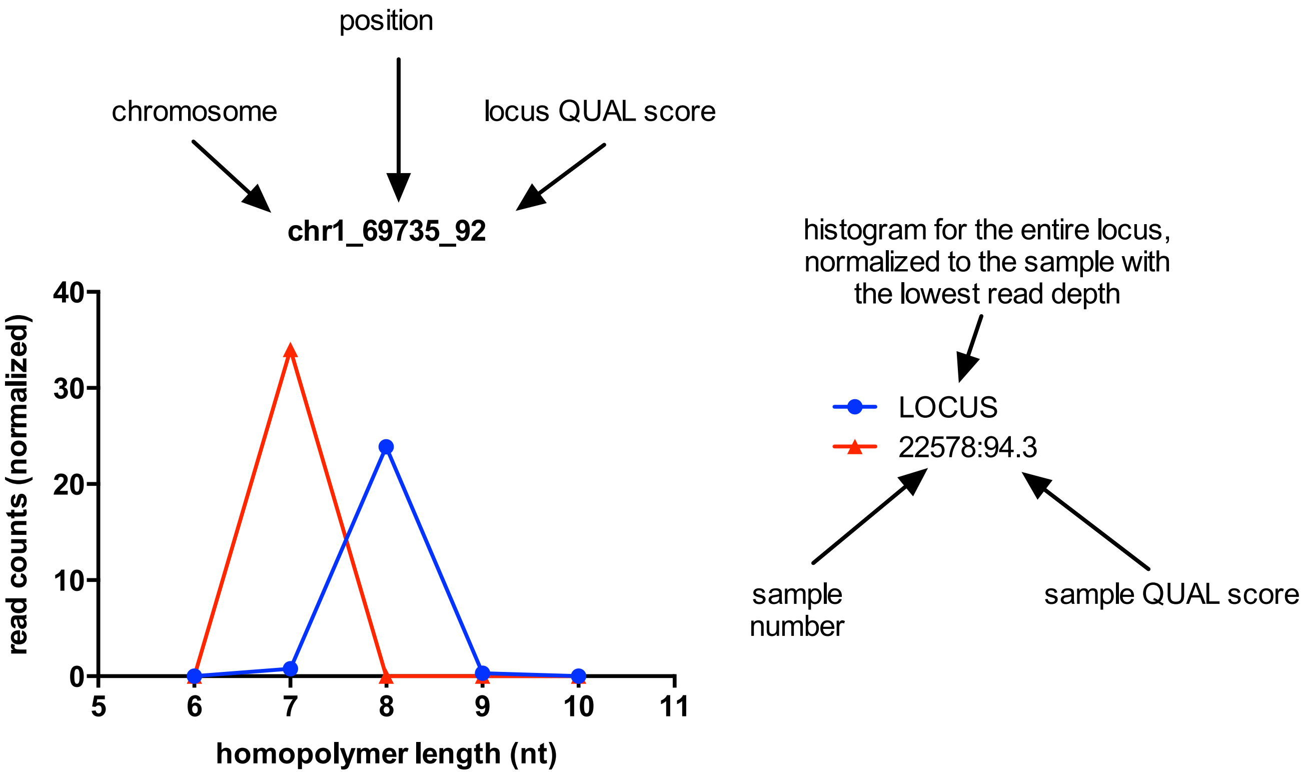

S4 File. Histograms for homopolymer loci called as having mutations by hp_caller. Each histogram shows the distribution of homopolymer lengths in all reads at the locus ("LOCUS") and the distribution of homopolymer lengths in reads from samples called as mutants (indicated by sample numbers). The read depth of the locus distribution is normalized to the read depth in the sample with the fewest reads at the locus, which is not necessarily the mutant sample. The read depths for the mutant sample distributions are not normalized. The title for each panel indicates the chromosome and start position of the homopolymer, as well as the hp_caller QUAL score for the locus. The two mutant calls at G:C homopolymers have "GC" appended to their titles; all other loci are A:T homopolymers. The legend indicates the sample name and the hp_caller QUAL score for mutant samples.

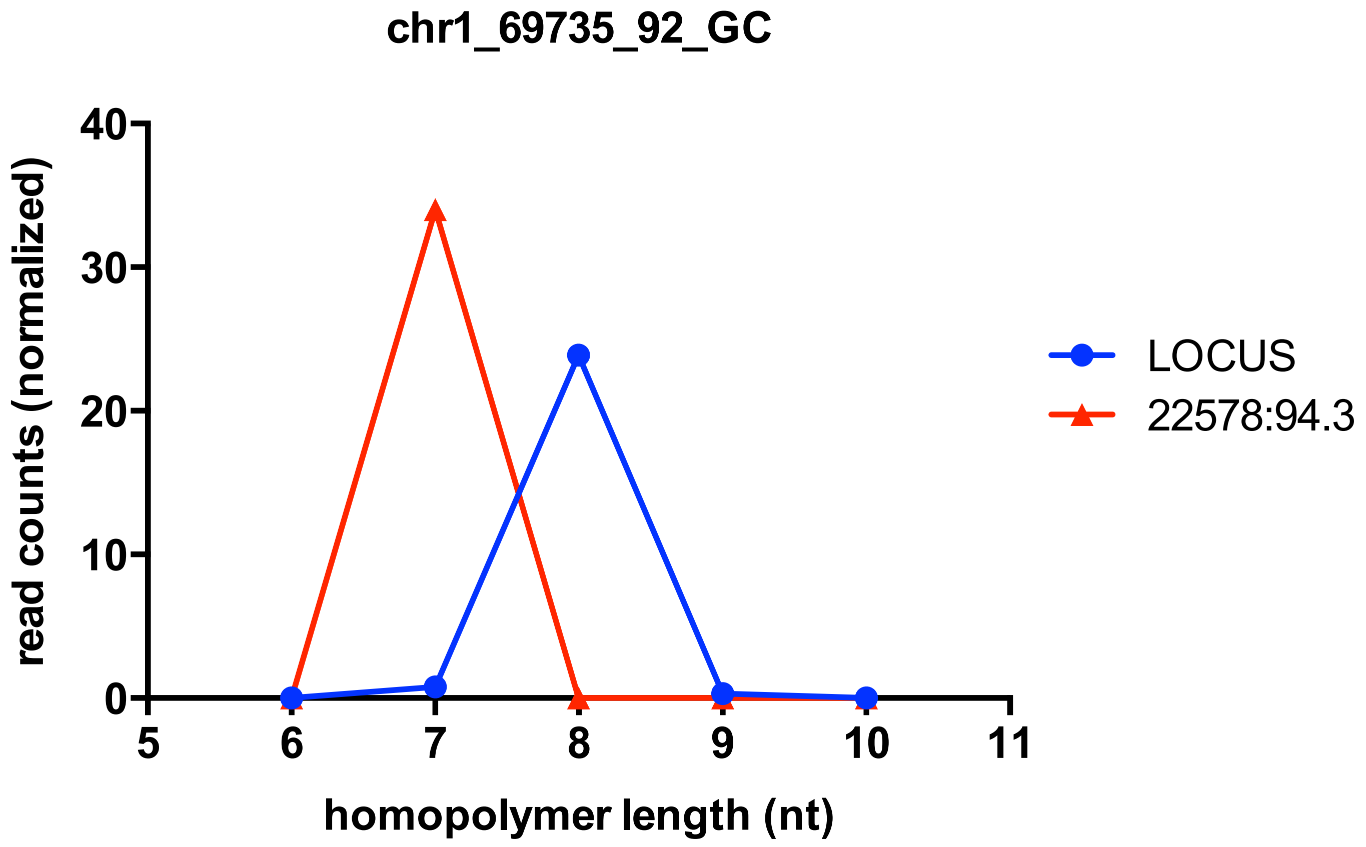
chr1_69735_92_GC
40
30
20
10
0
read counts (normalized)
5
6
7
8
9
10
11
homopolymer length (nt)
LOCUS
22578:94.3

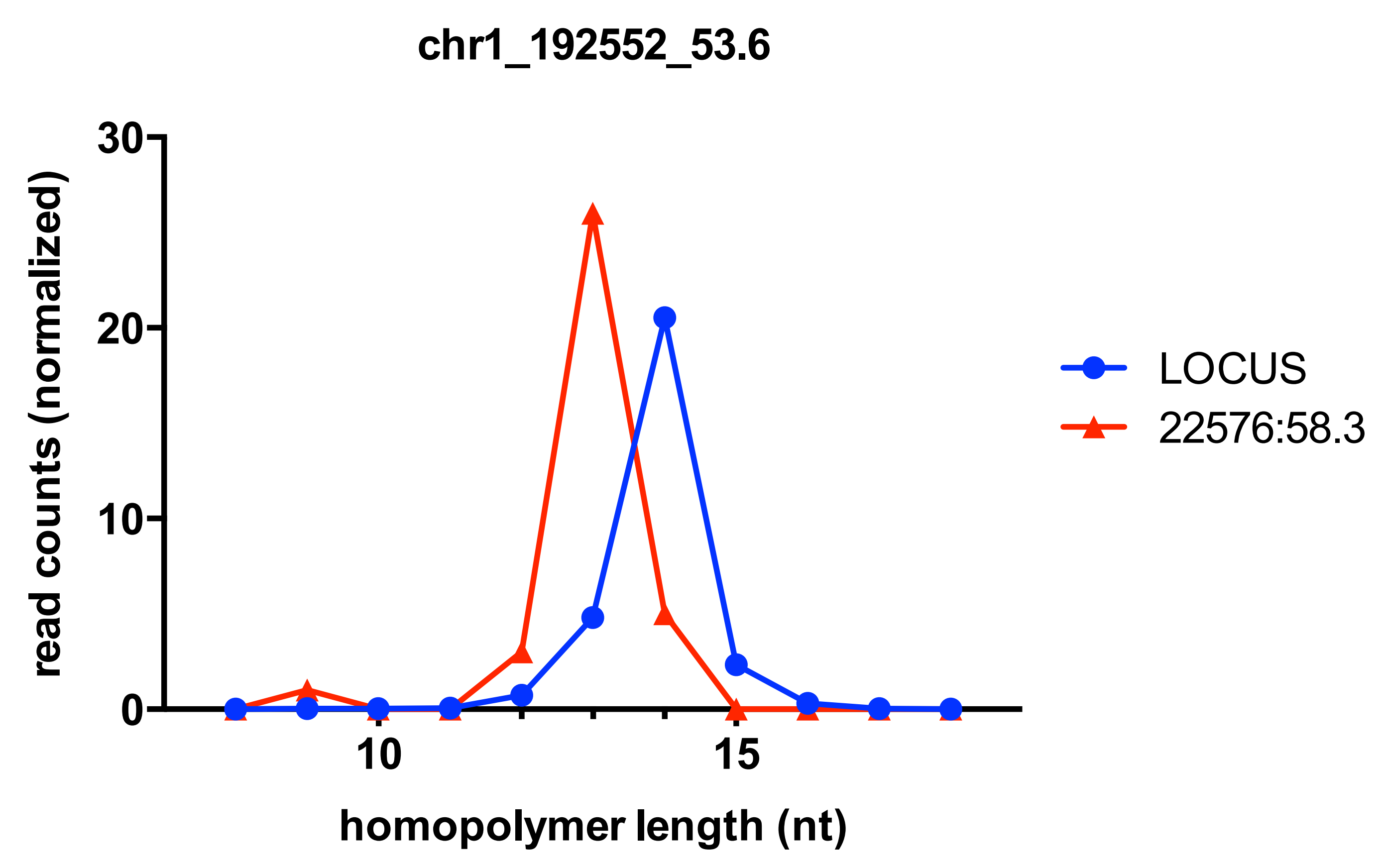

chr1_192552_53.6
30
20
10
0
read counts (normalized)
10
15
homopolymer length (nt)
LOCUS
22576:58.3

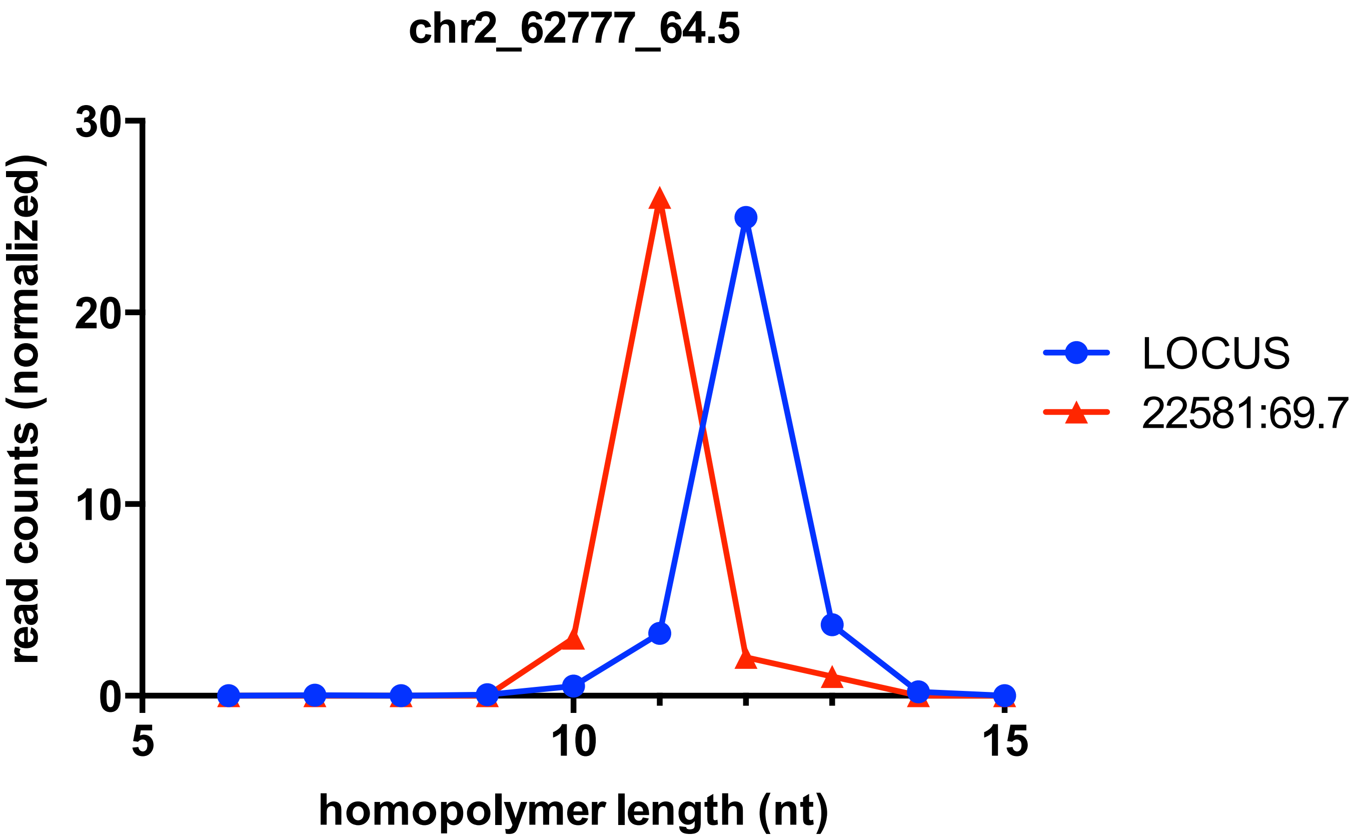
chr2_62777_64.5
read counts (normalized)
30
20
10
0
5
10
15
homopolymer length (nt)
LOCUS
22581:69.7

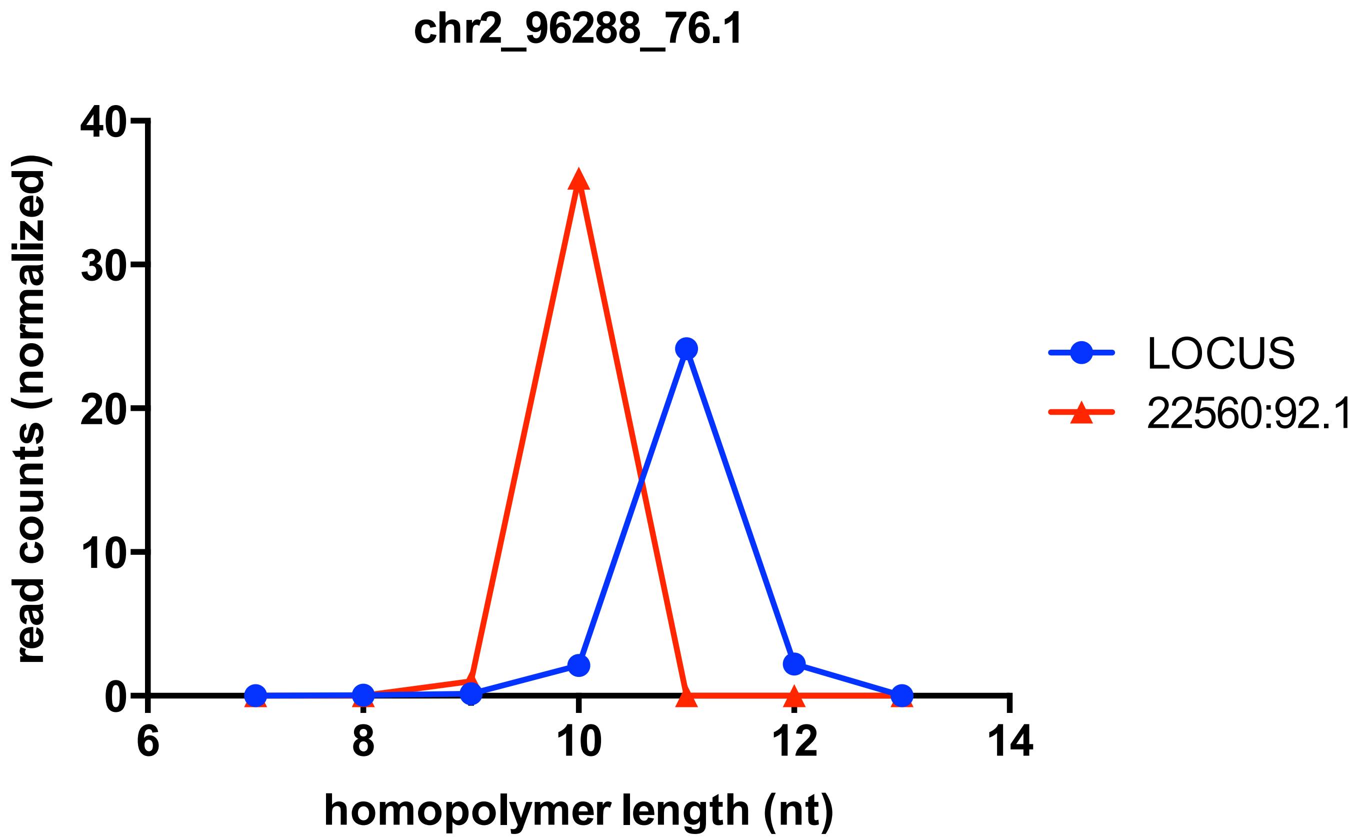
chr2_96288_76.1
read counts (normalized)
40
30
20
10
0
6
8
10
12
14
homopolymer length (nt)
LOCUS
22560:92.1

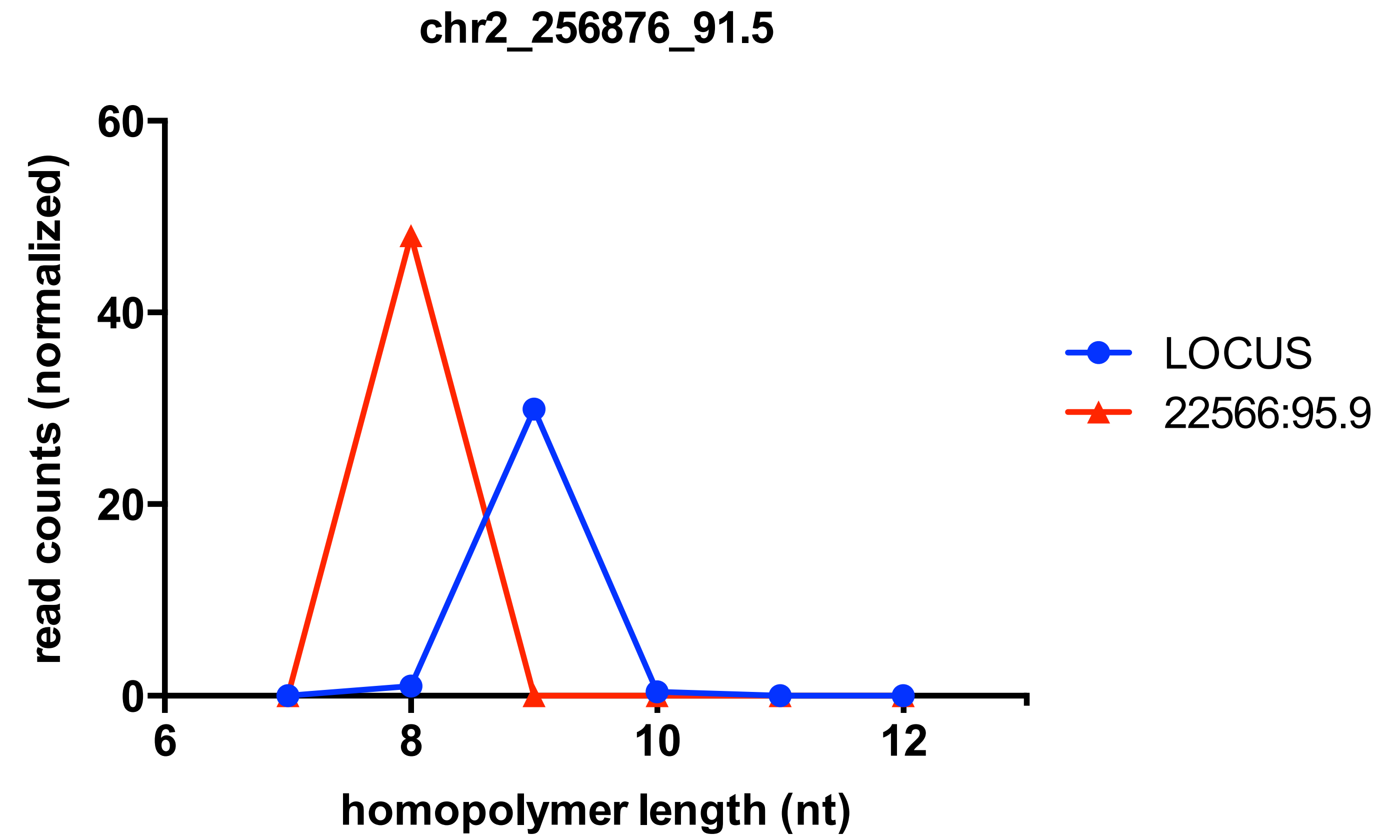
chr2_256876_91.5
read counts (normalized)
60
40
20
0
6
8
10
12
homopolymer length (nt)
LOCUS
22566:95.9

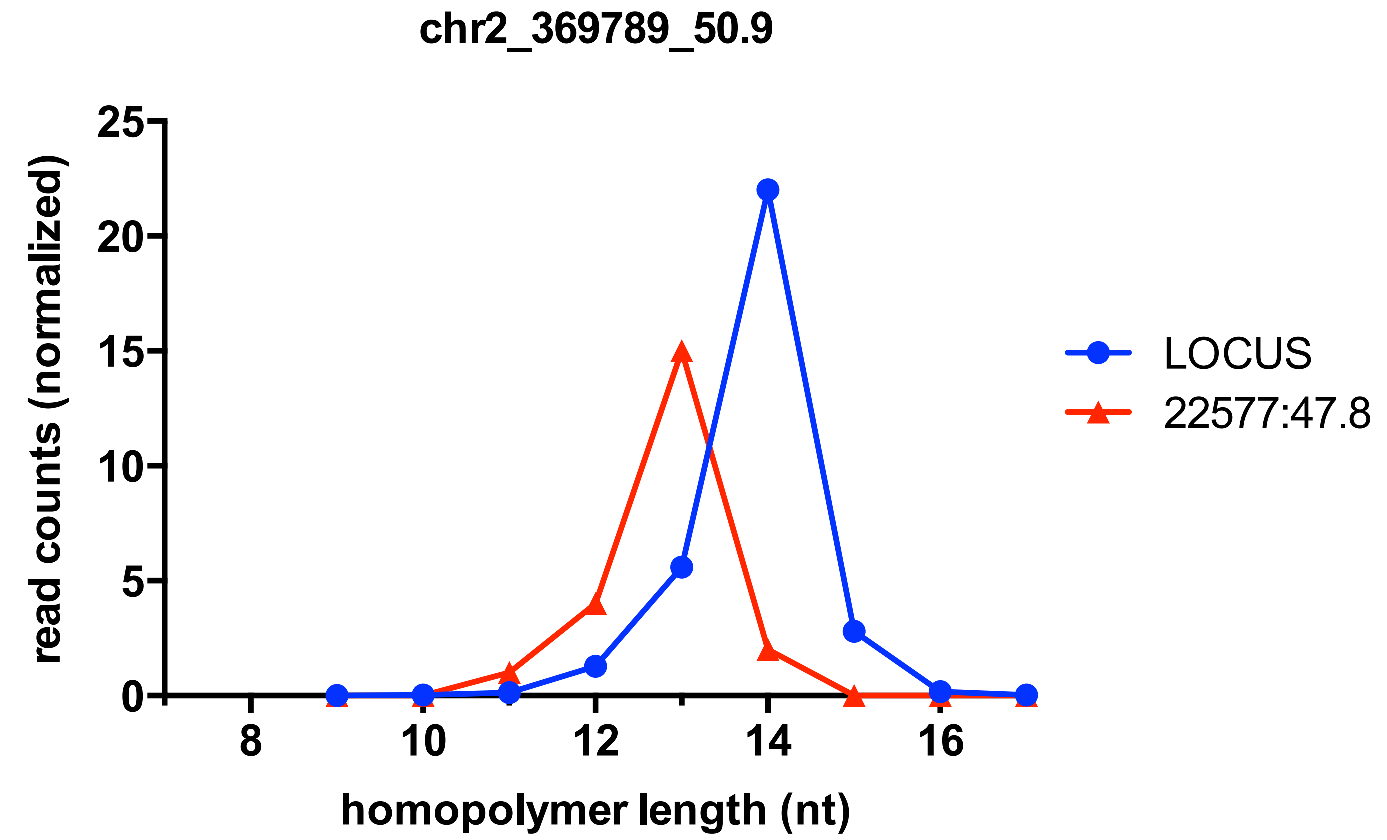
chr2_369789_50.9
read counts (normalized)
25
20
15
10
5
0
8
10
12
14
16
homopolymer length (nt)
LOCUS
22577:47.8

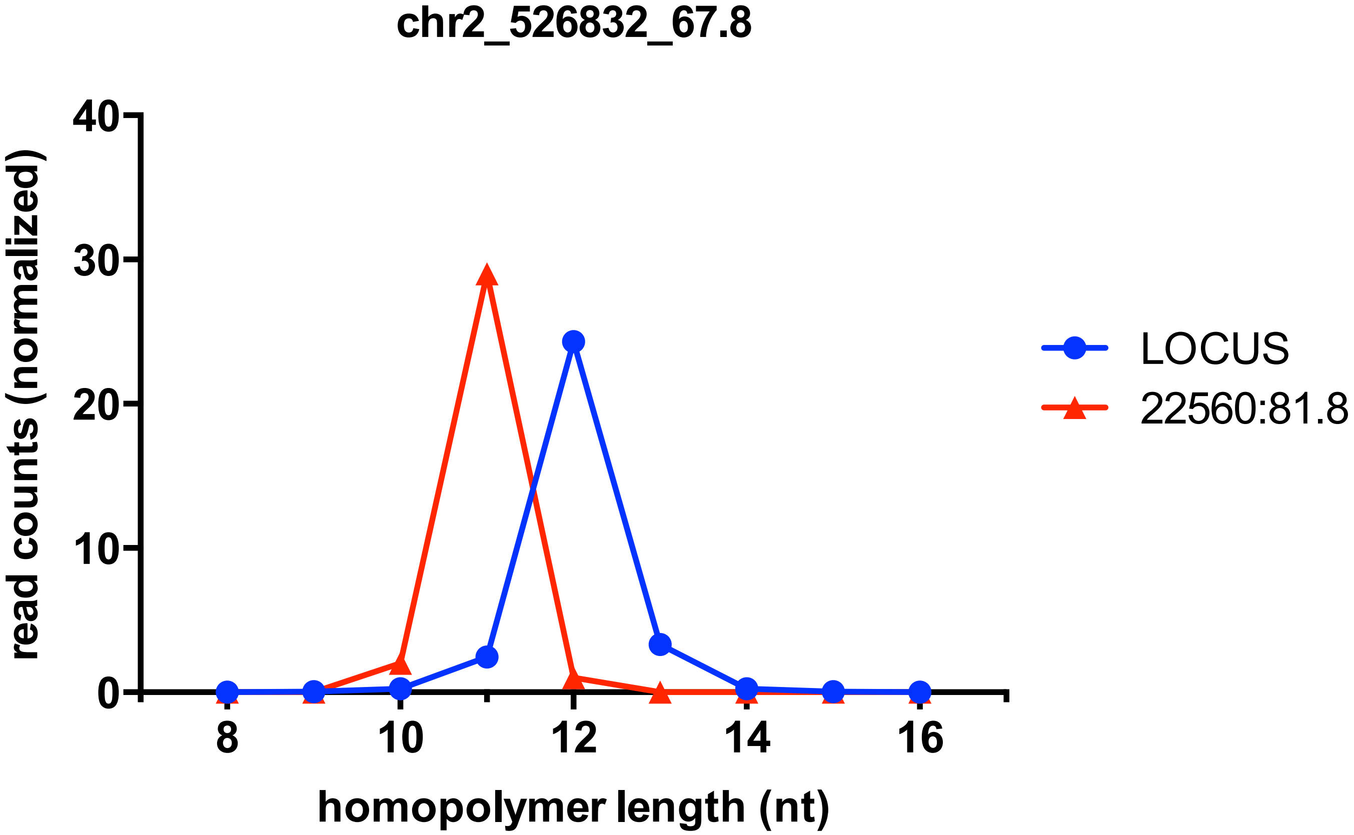

chr2_526832_67.8
read counts (normalized)
0
10
20
30
40
8
10
12
14
16
homopolymer length (nt)
LOCUS
22560:81.8

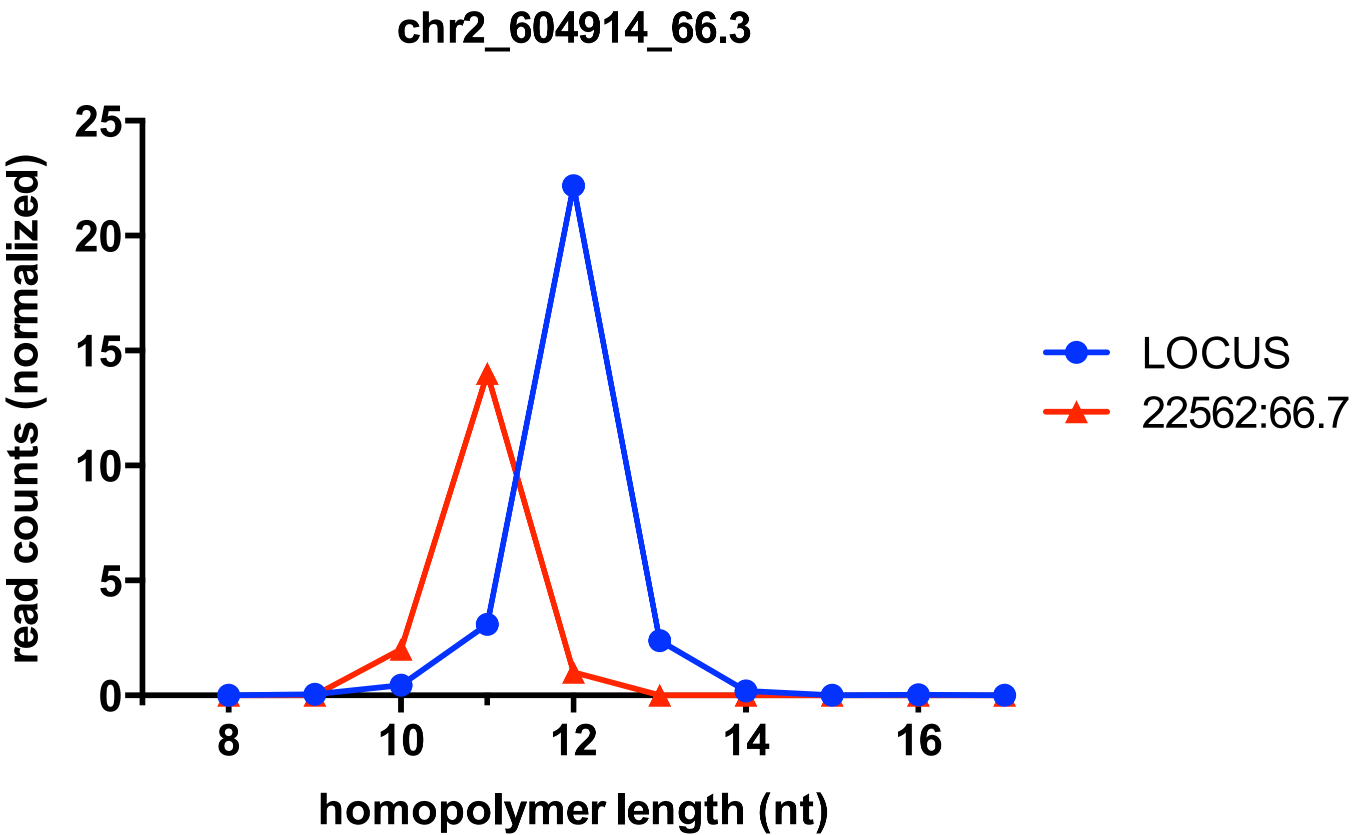
chr2_604914_66.3
read counts (normalized)
0
5
10
15
20
25
8
10
12
14
16
homopolymer length (nt)
LOCUS
22562:66.7

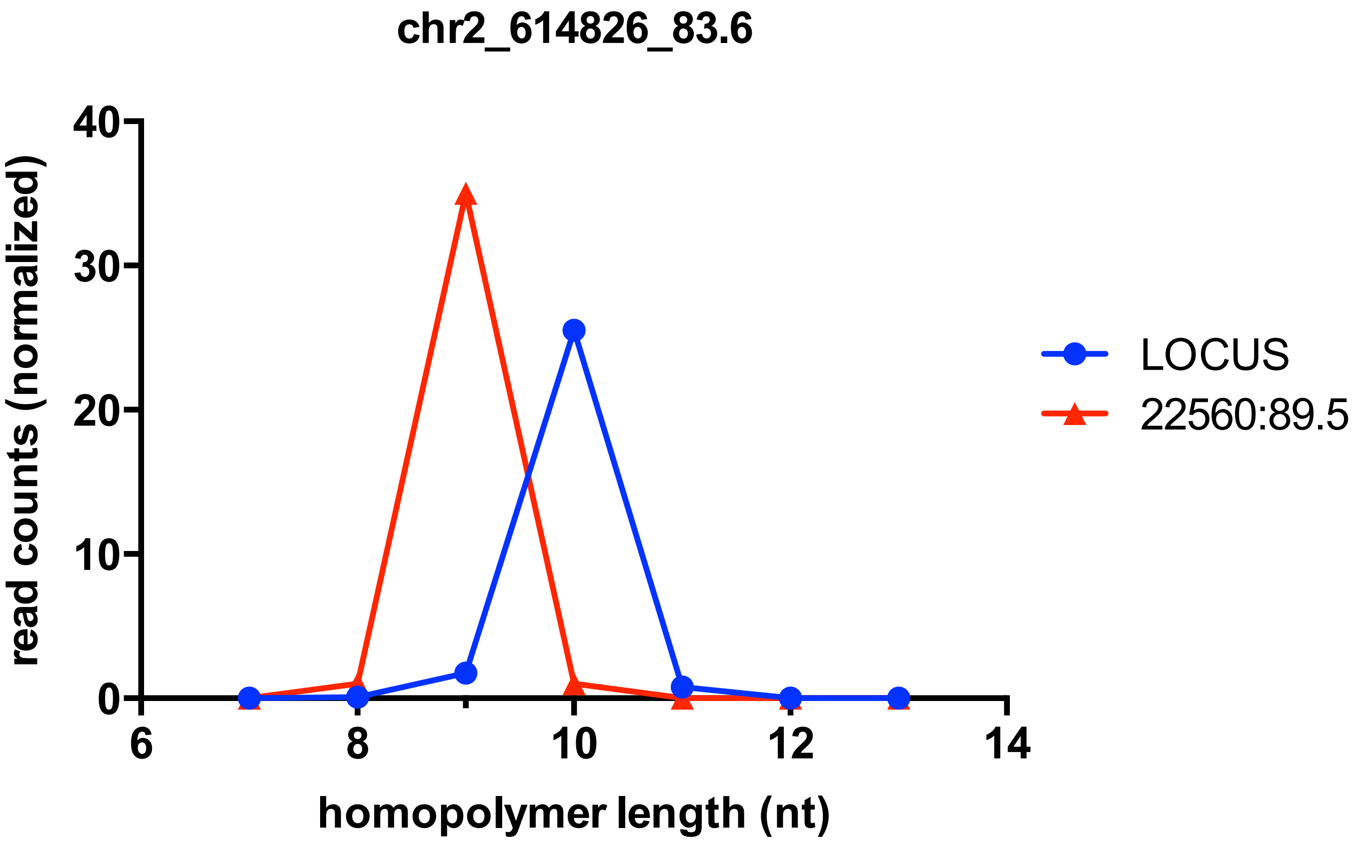

chr2_614826_83.6
read counts (normalized)
0
10
20
30
40
6
8
10
12
14
homopolymer length (nt)
LOCUS
22560:89.5

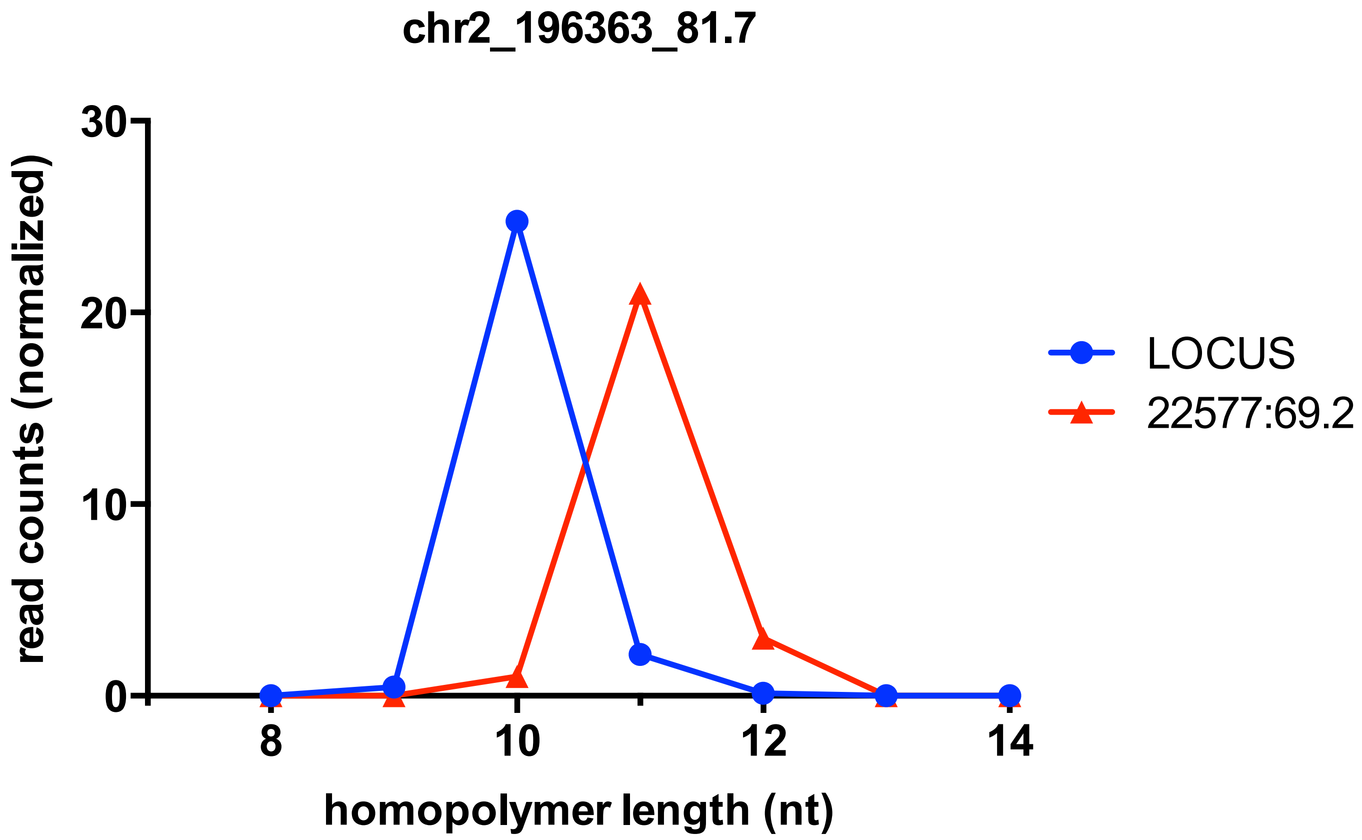
chr2_196363_81.7
30
20
10
0
read counts (normalized)
8
10
12
14
homopolymer length (nt)
LOCUS
22577:69.2

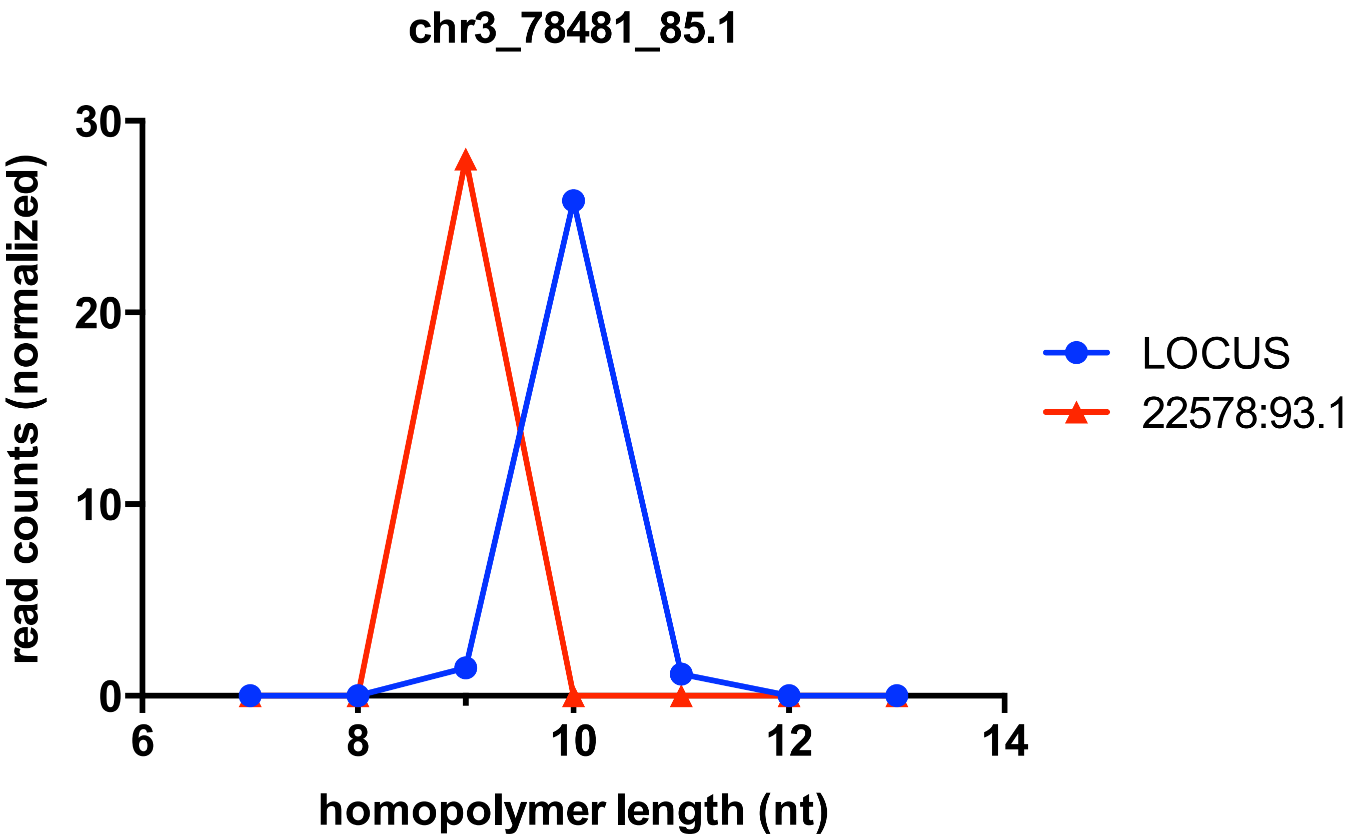
chr3_78481_85.1
read counts (normalized)
30
20
10
0
6
8
10
12
14
homopolymer length (nt)
LOCUS
22578:93.1

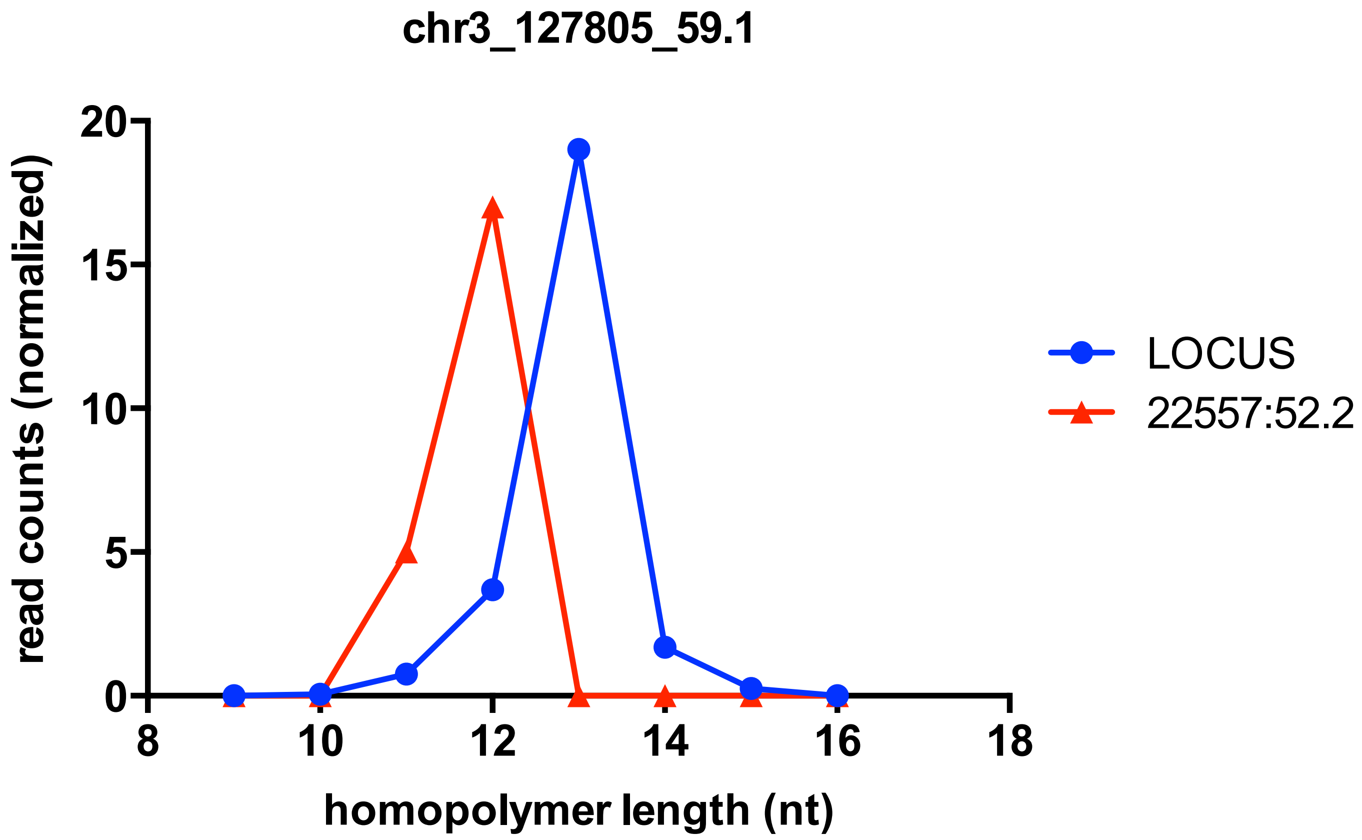
chr3_127805_59.1
20
15
10
5
0
read counts (normalized)
8
10
12
14
16
18
homopolymer length (nt)
LOCUS
22557:52.2

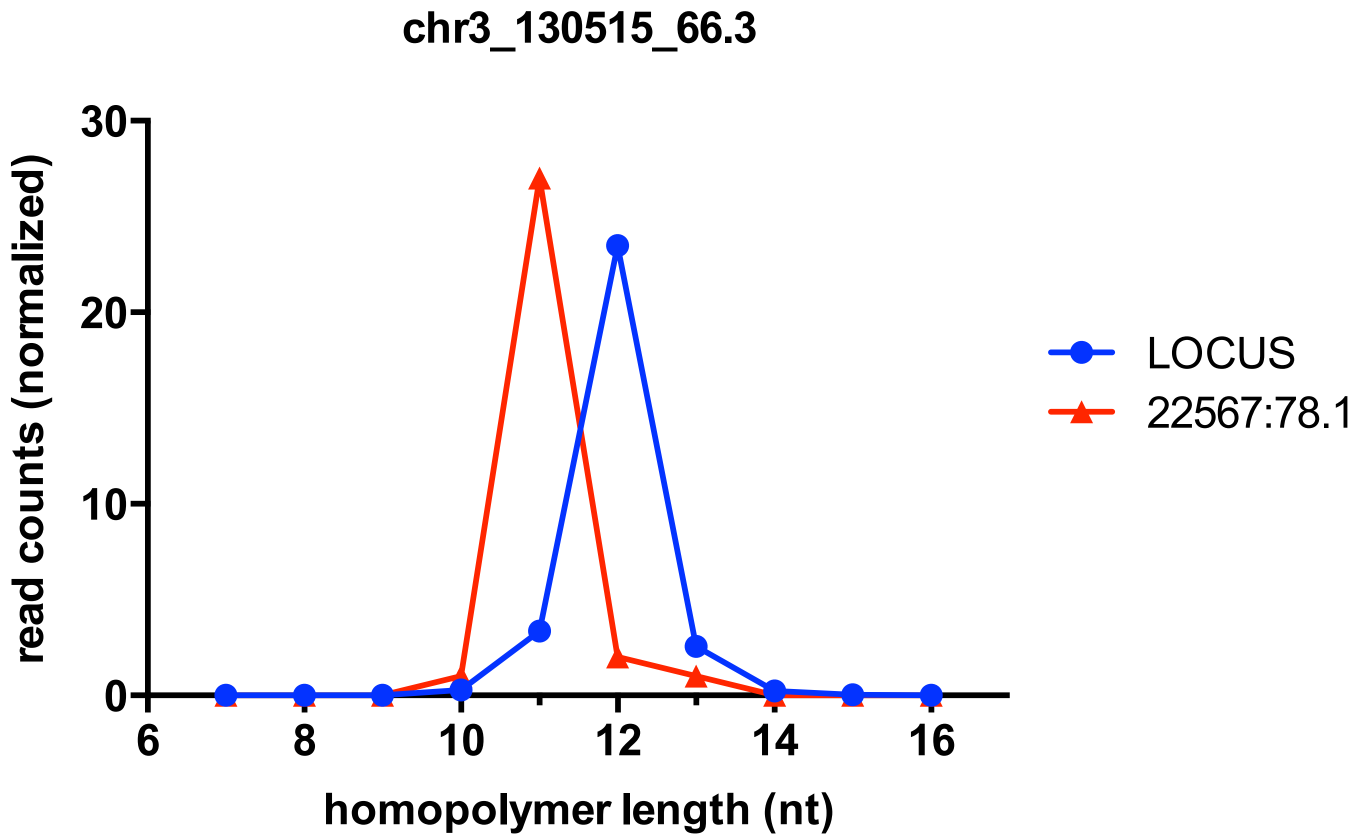

chr3_130515_66.3
read counts (normalized)
30
20
10
0
6
8
10
12
14
16
homopolymer length (nt)
LOCUS
22567:78.1

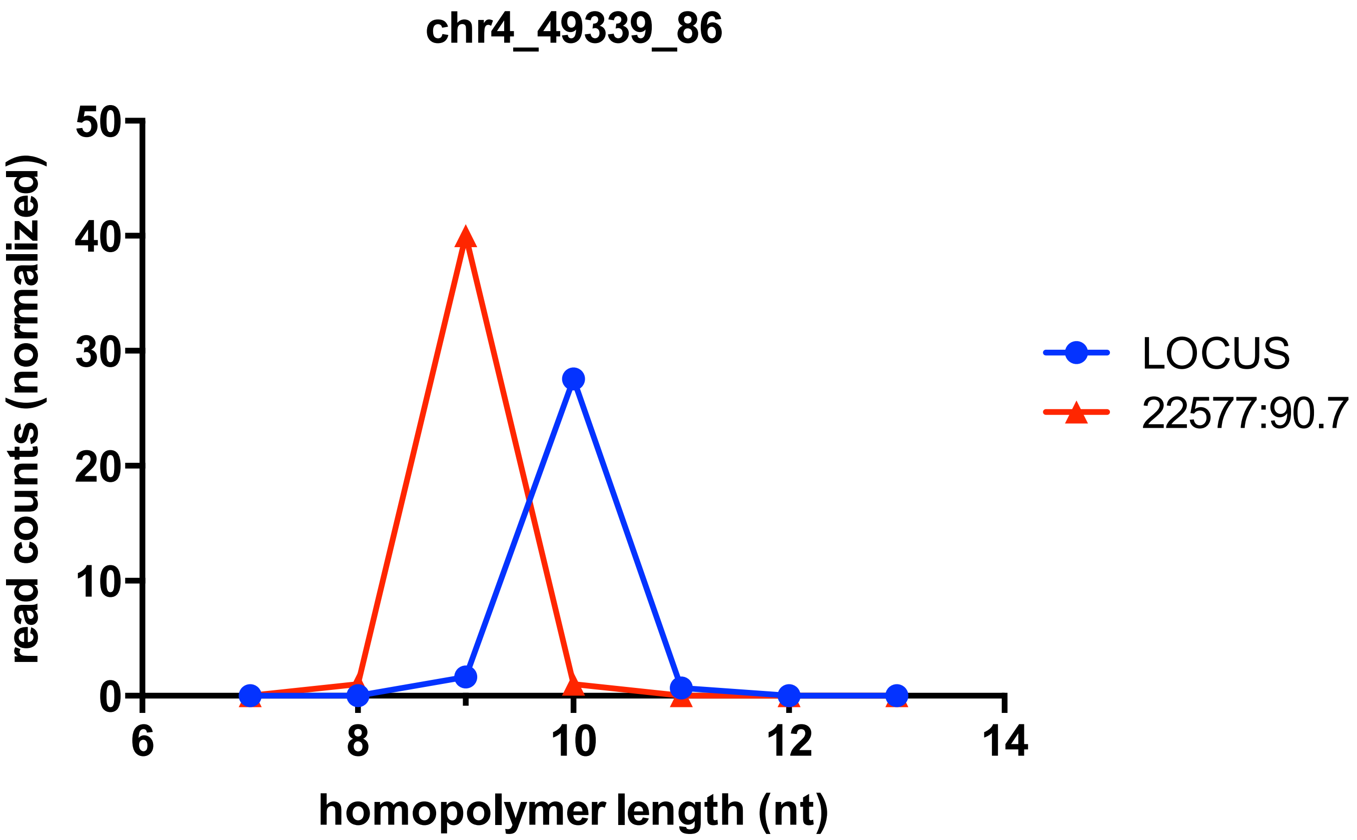
chr4_49339_86
read counts (normalized)
50
40
30
20
10
0
6
8
10
12
14
homopolymer length (nt)
LOCUS
22577:90.7

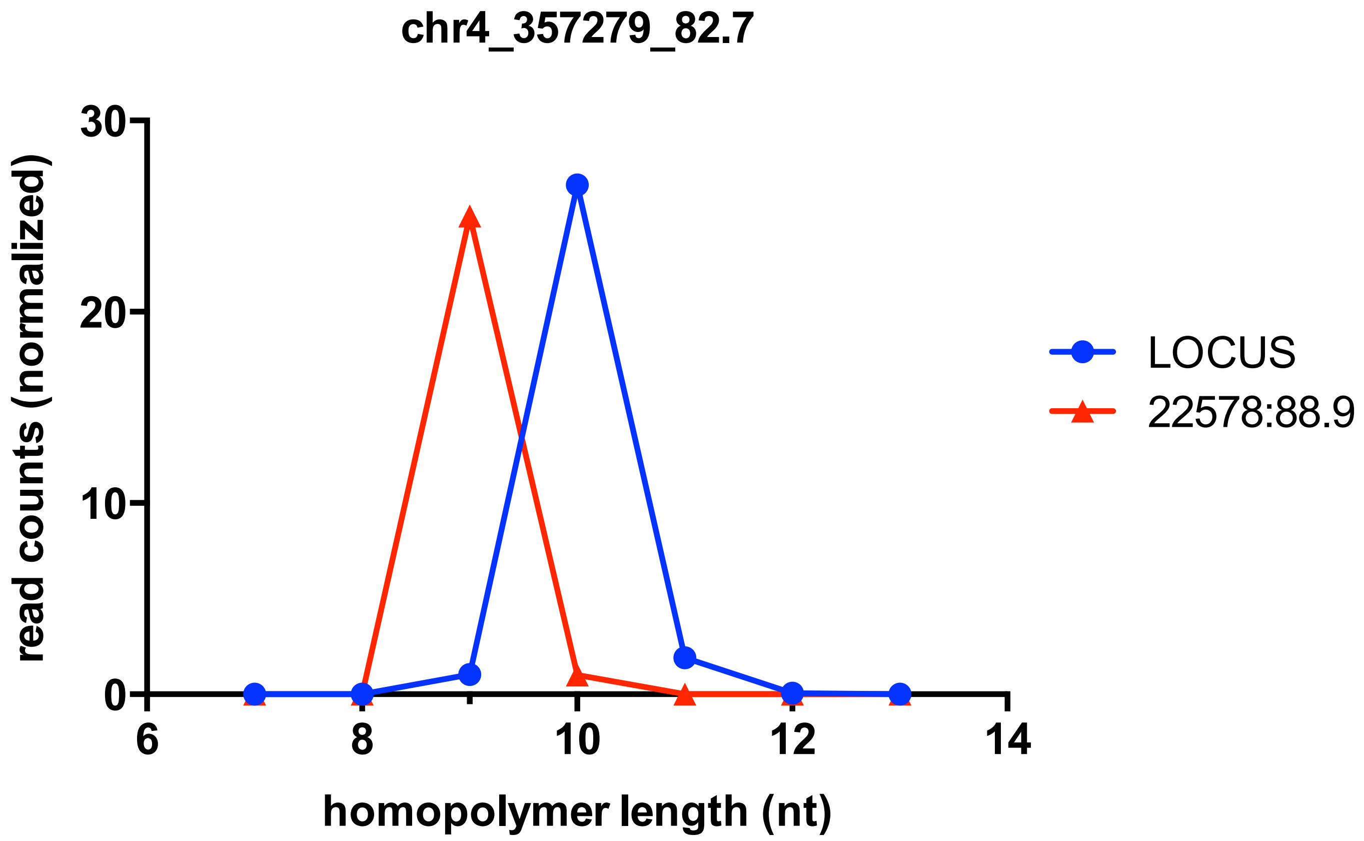
chr4_357279_82.7
read counts (normalized)
30
20
10
0
6
8
10
12
14
homopolymer length (nt)
LOCUS
22578:88.9

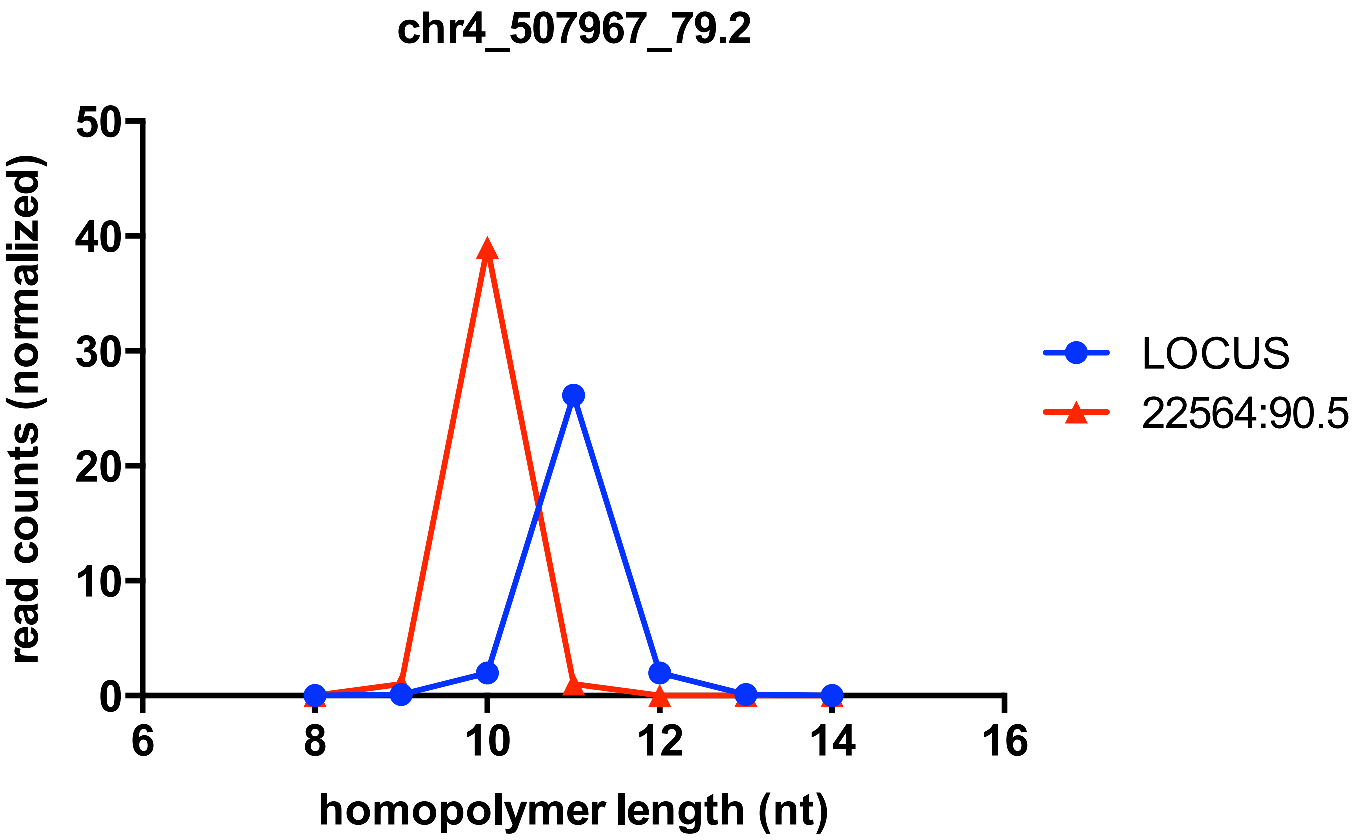
chr4_507967_79.2
read counts (normalized)
50
40
30
20
10
0
6
8
10
12
14
16
homopolymer length (nt)
LOCUS
22564:90.5

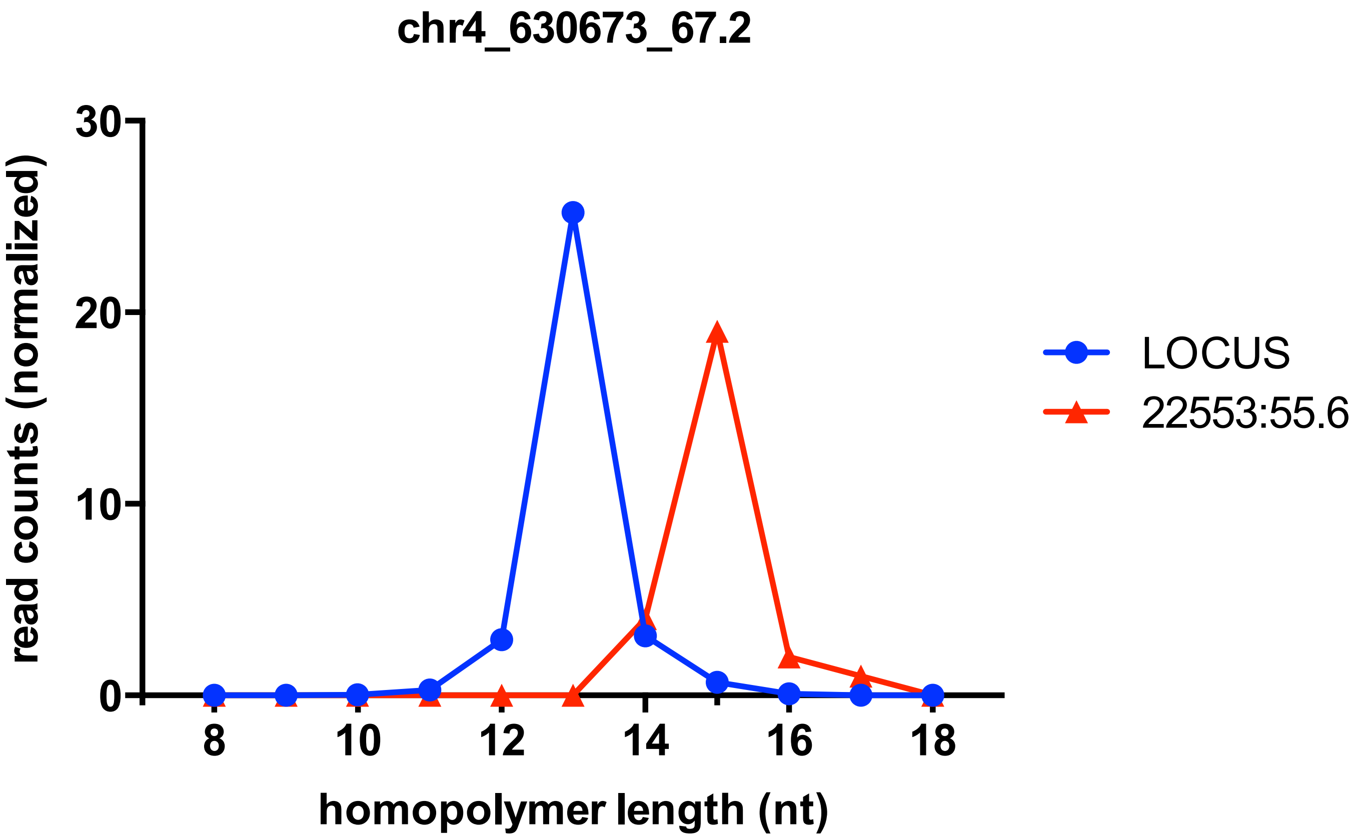
chr4_630673_67.2
read counts (normalized)
30
20
10
0
8
10
12
14
16
18
homopolymer length (nt)
LOCUS
22553:55.6

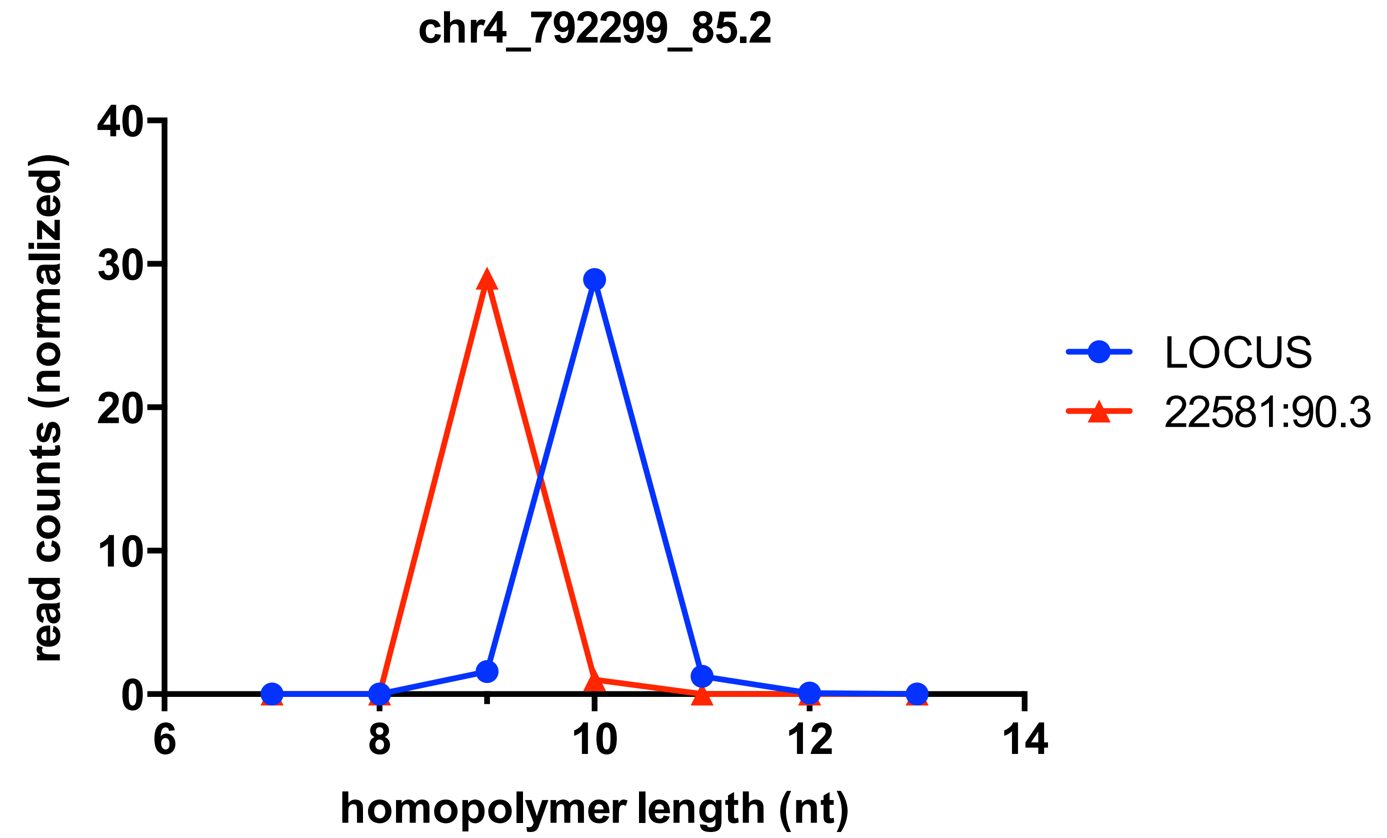
chr4_792299_85.2
read counts (normalized)
0
10
20
30
40
6
8
10
12
14
homopolymer length (nt)
LOCUS
22581:90.3

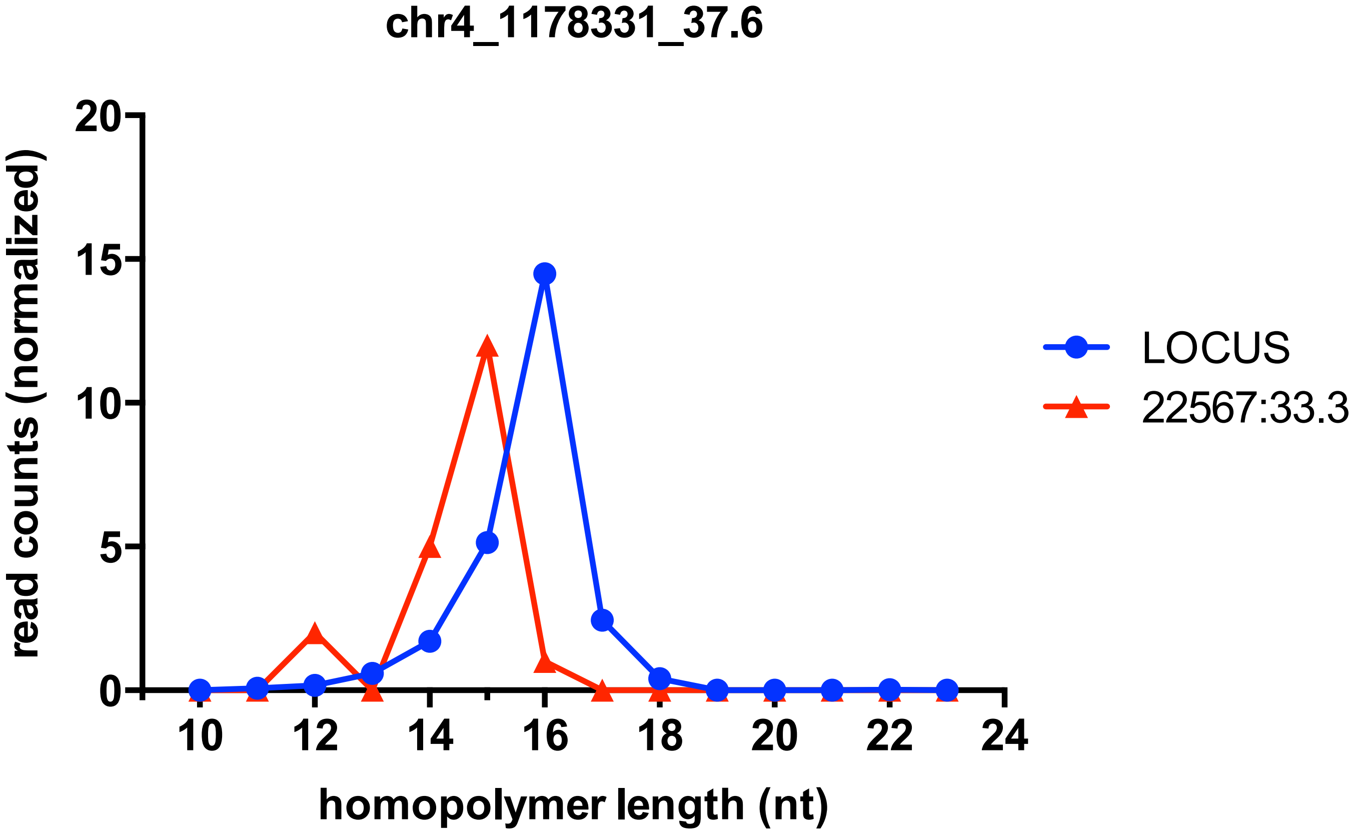
chr4_1178331_37.6
read counts (normalized)
homopolymer length (nt)
0
5
10
15
20
10
12
14
16
18
20
22
24
LOCUS
22567:33.3

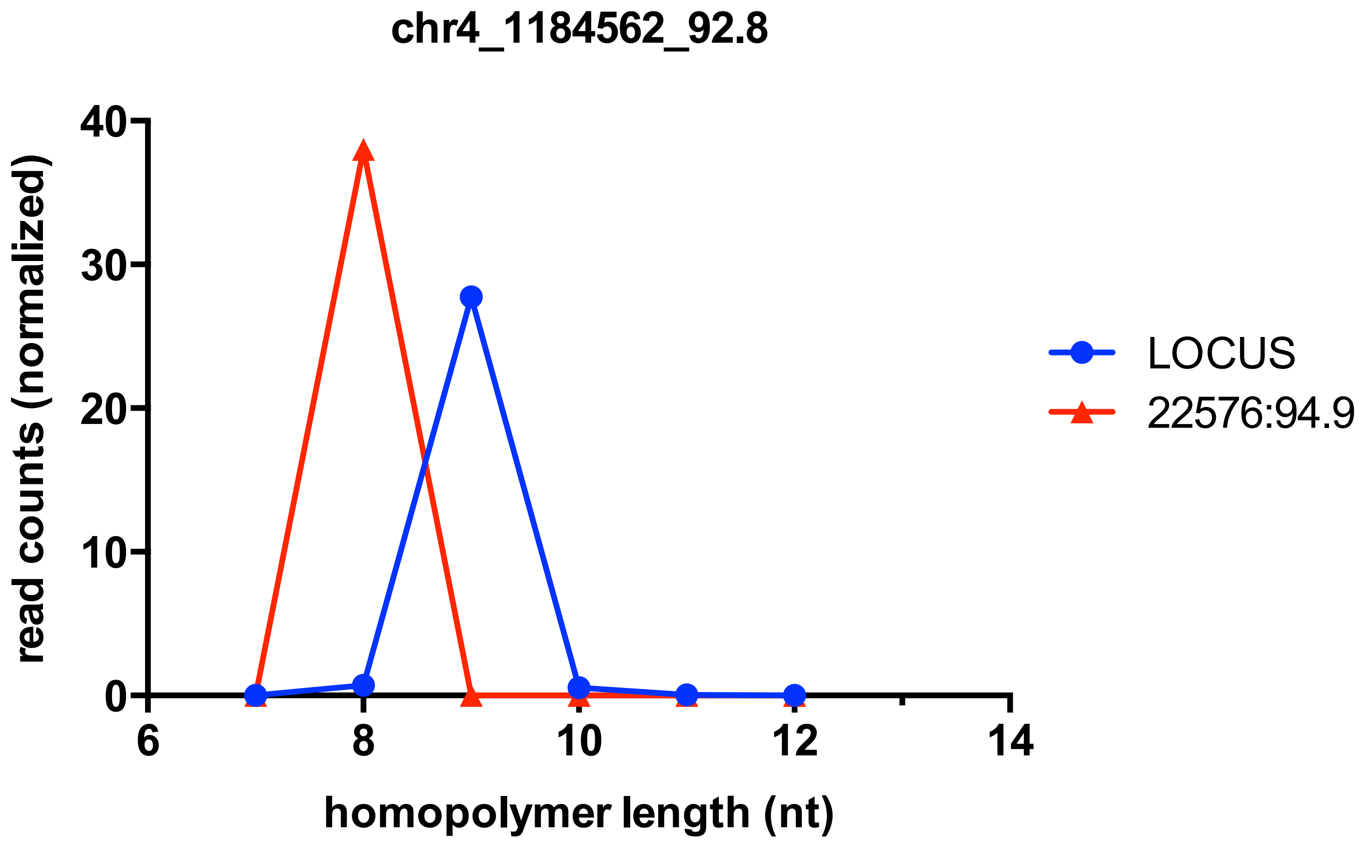
chr4_1184562_92.8
read counts (normalized)
40
30
20
10
0
6
8
10
12
14
homopolymer length (nt)
LOCUS
22576:94.9

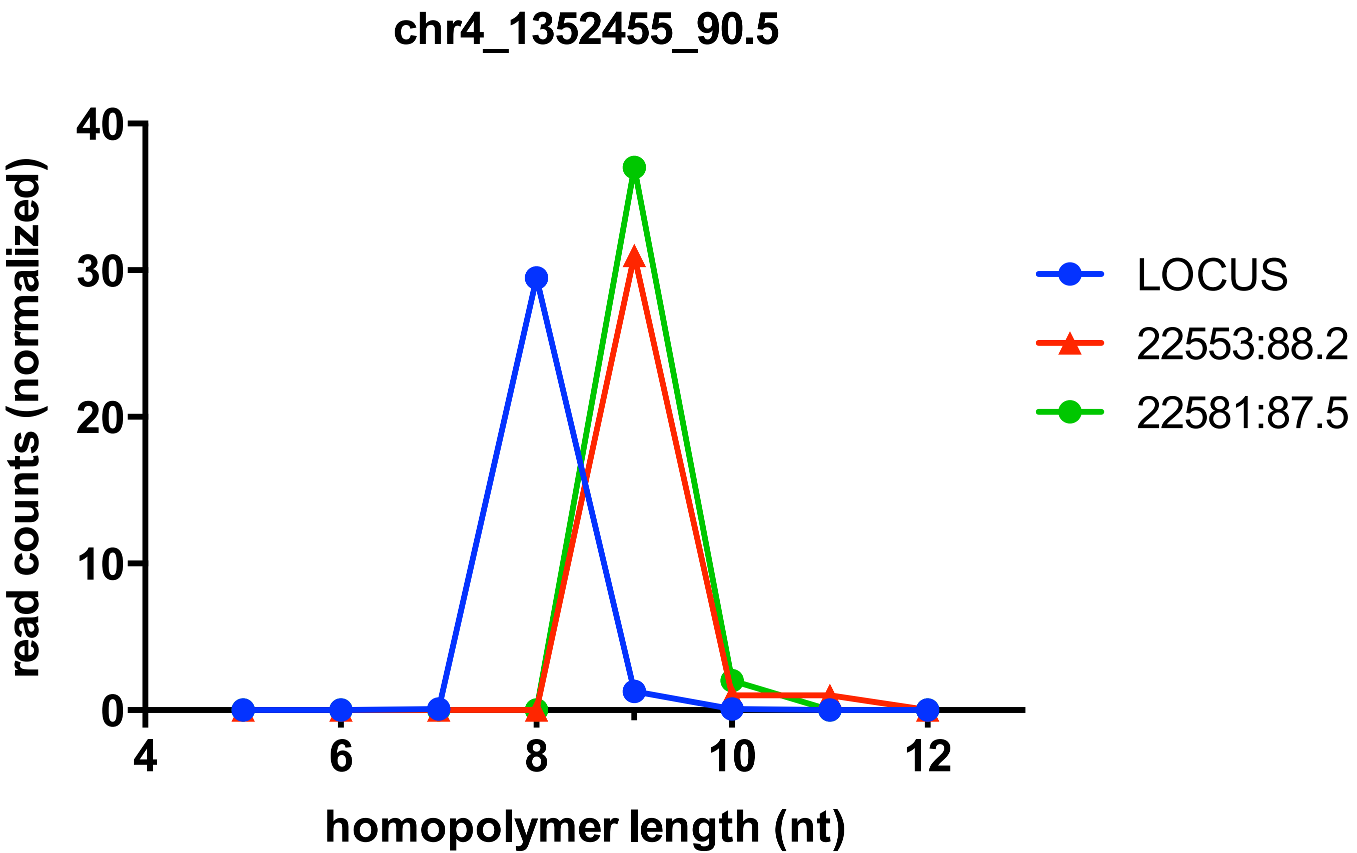
chr4_1352455_90.5
read counts (normalized)
0
10
20
30
40
4
6
8
10
12
homopolymer length (nt)
LOCUS
22553:88.2
22581:87.5

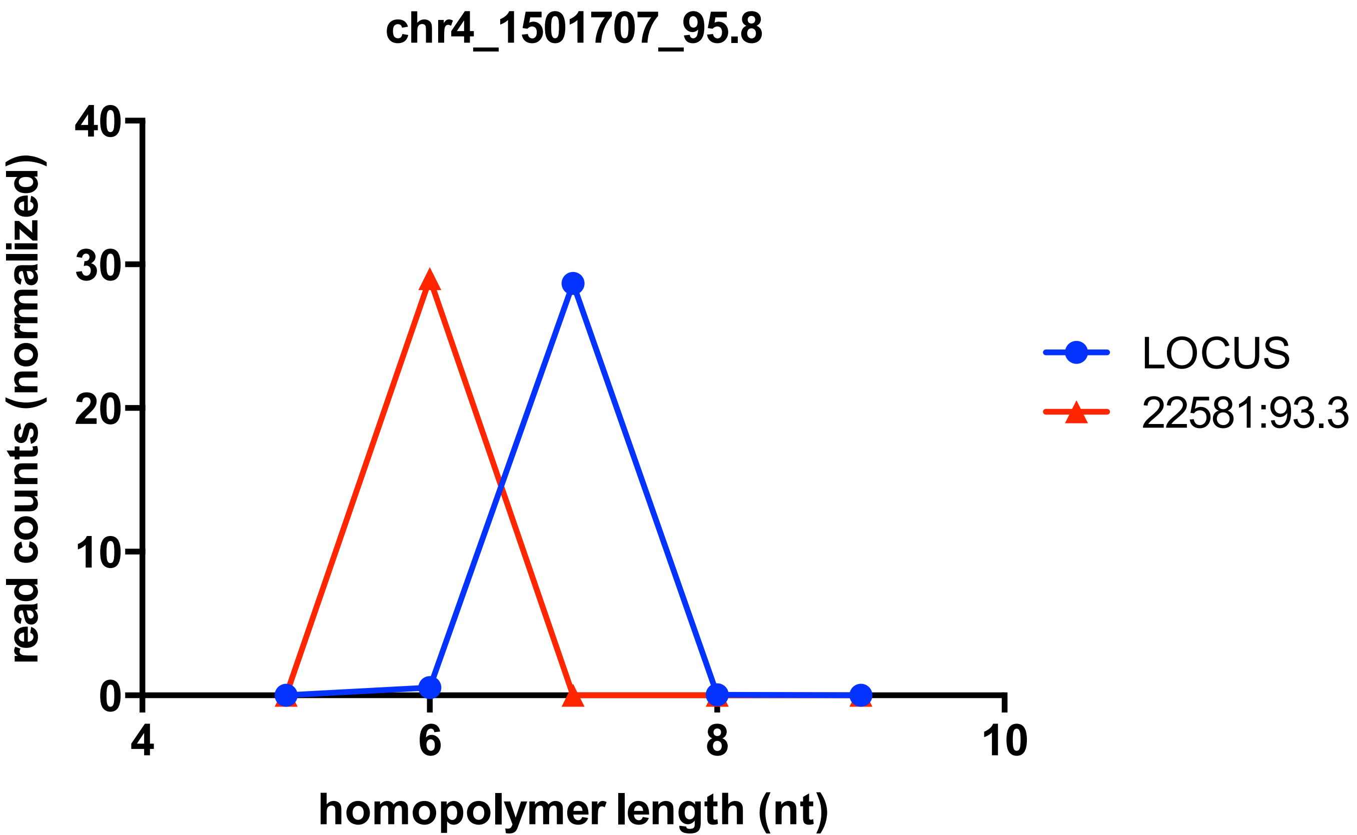

chr4_1501707_95.8
read counts (normalized)
0
10
20
30
40
4
6
8
10
homopolymer length (nt)
LOCUS
22581:93.3

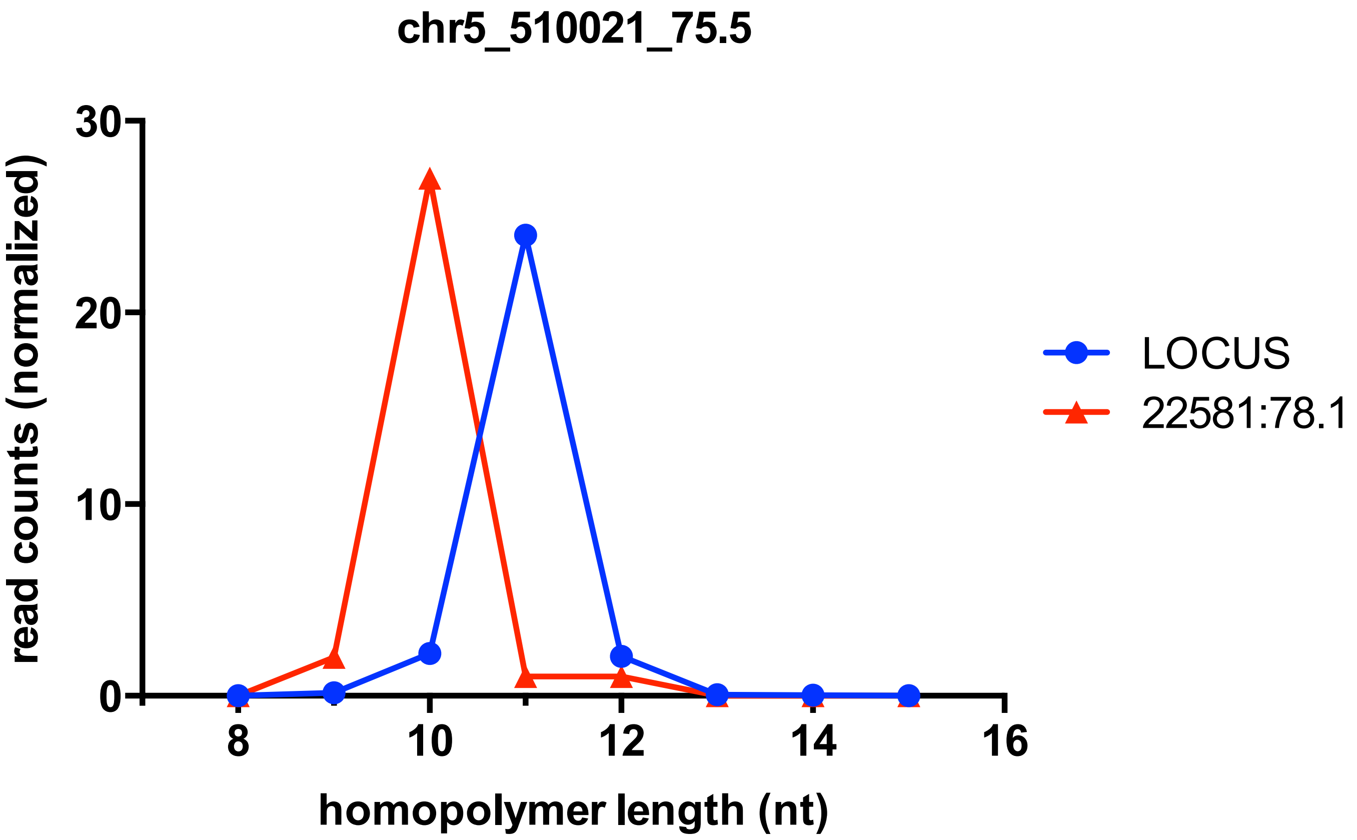

chr5_510021_75.5
read counts (normalized)
30
20
10
0
8
10
12
14
16
homopolymer length (nt)
LOCUS
22581:78.1

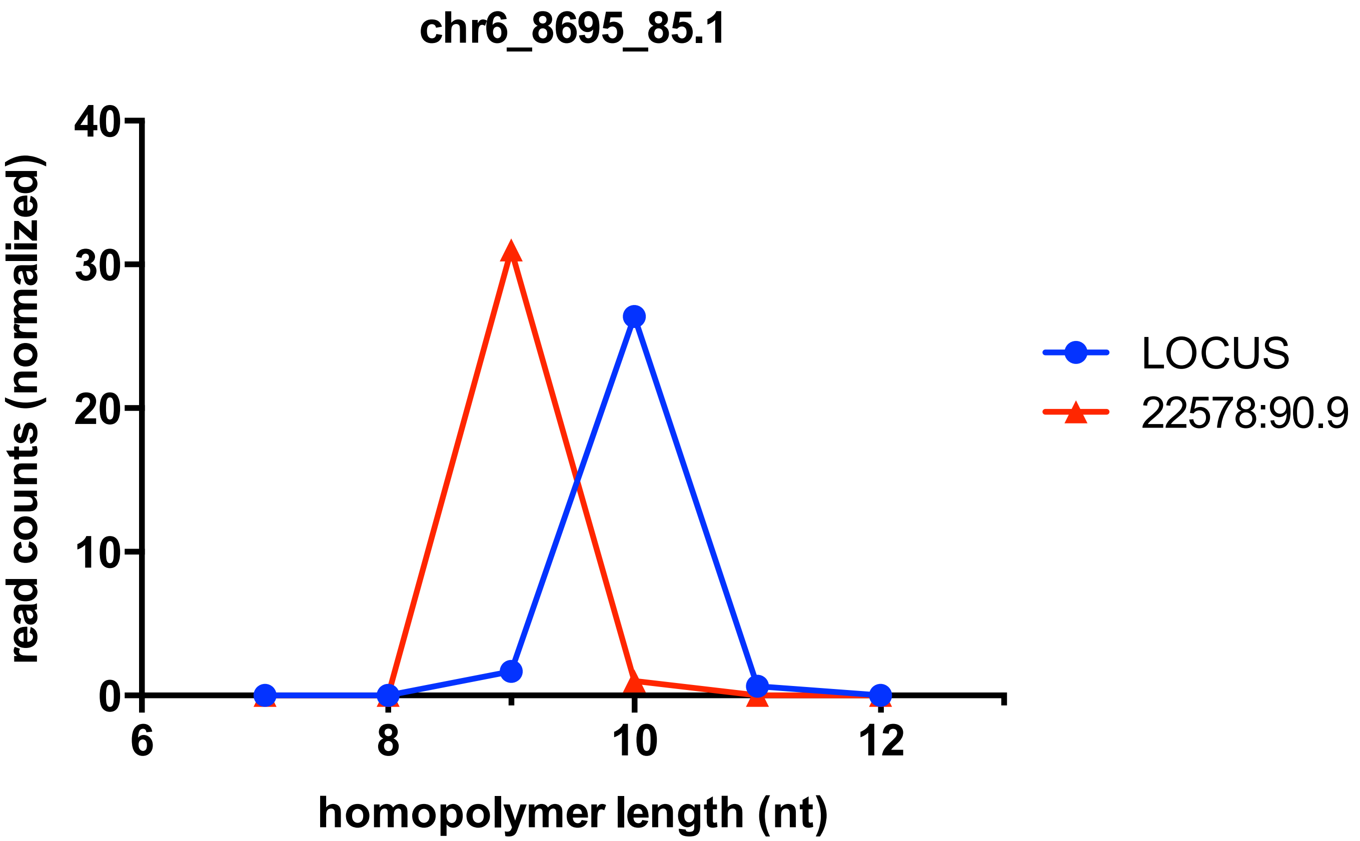

chr6_8695_85.1
read counts (normalized)
40
30
20
10
0
6
8
10
12
homopolymer length (nt)
LOCUS
22578:90.9

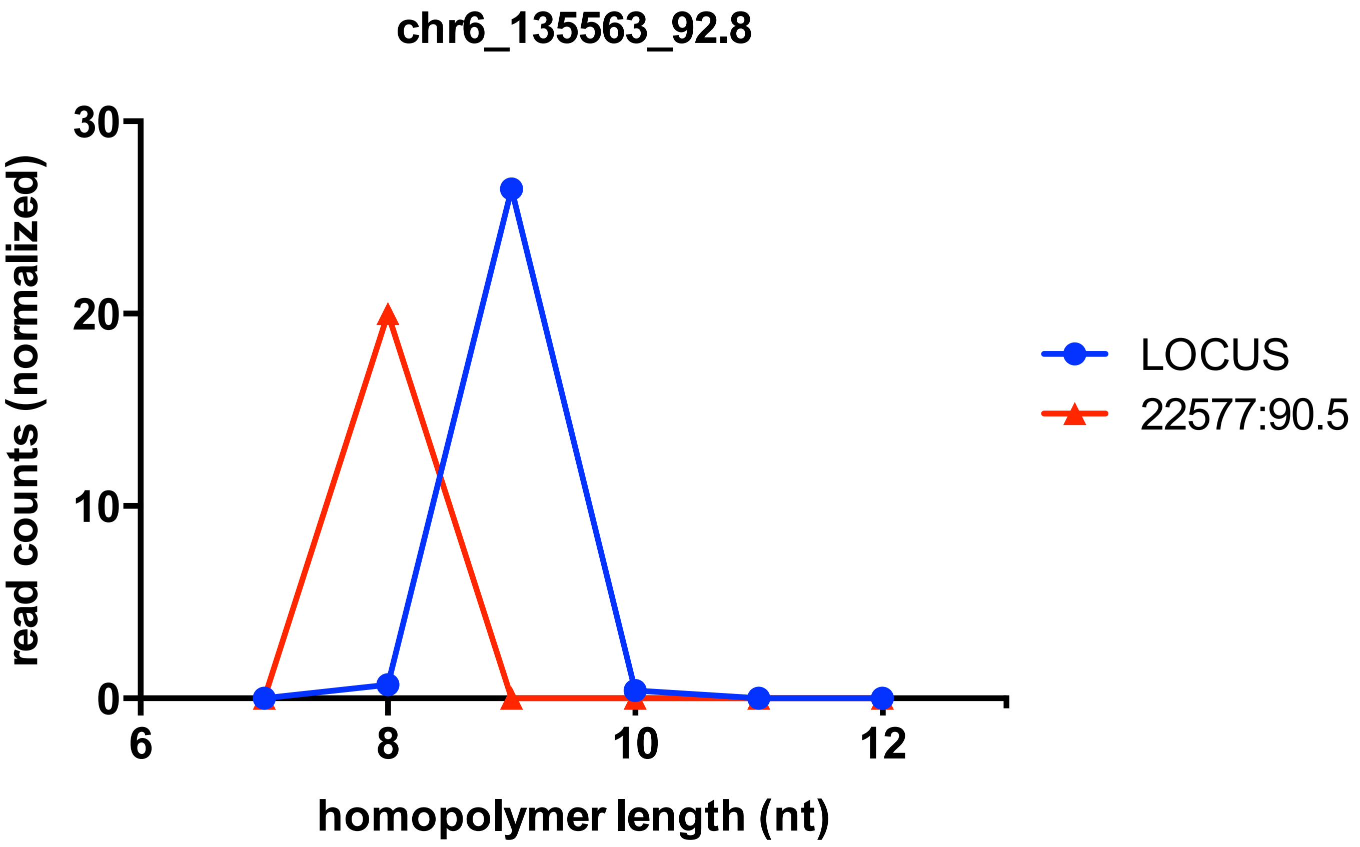

chr6_135563_92.8
30
20
10
0
6
8
10
12
read counts (normalized)
homopolymer length (nt)
LOCUS
22577:90.5

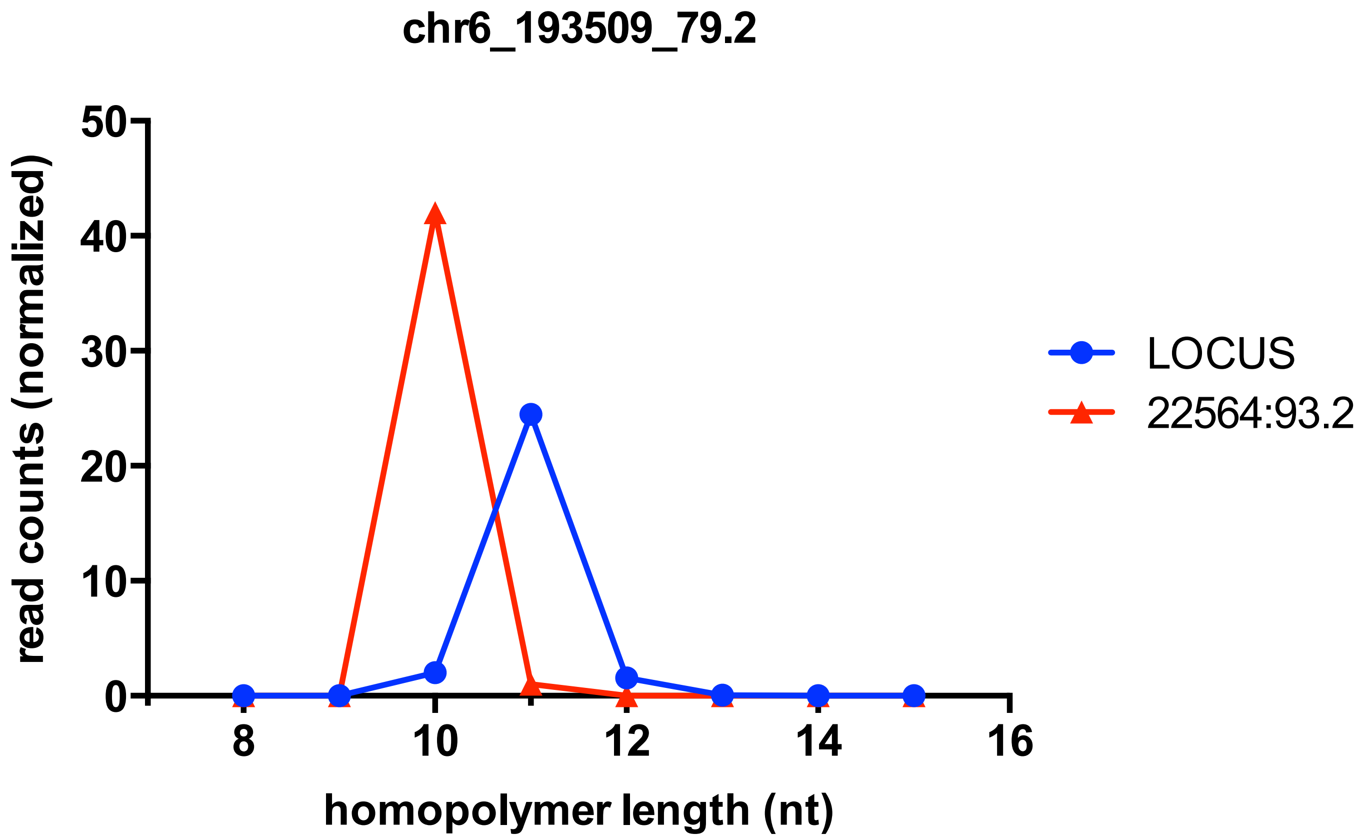
chr6_193509_79.2
read counts (normalized)
50
40
30
20
10
0
8
10
12
14
16
homopolymer length (nt)
LOCUS
22564:93.2

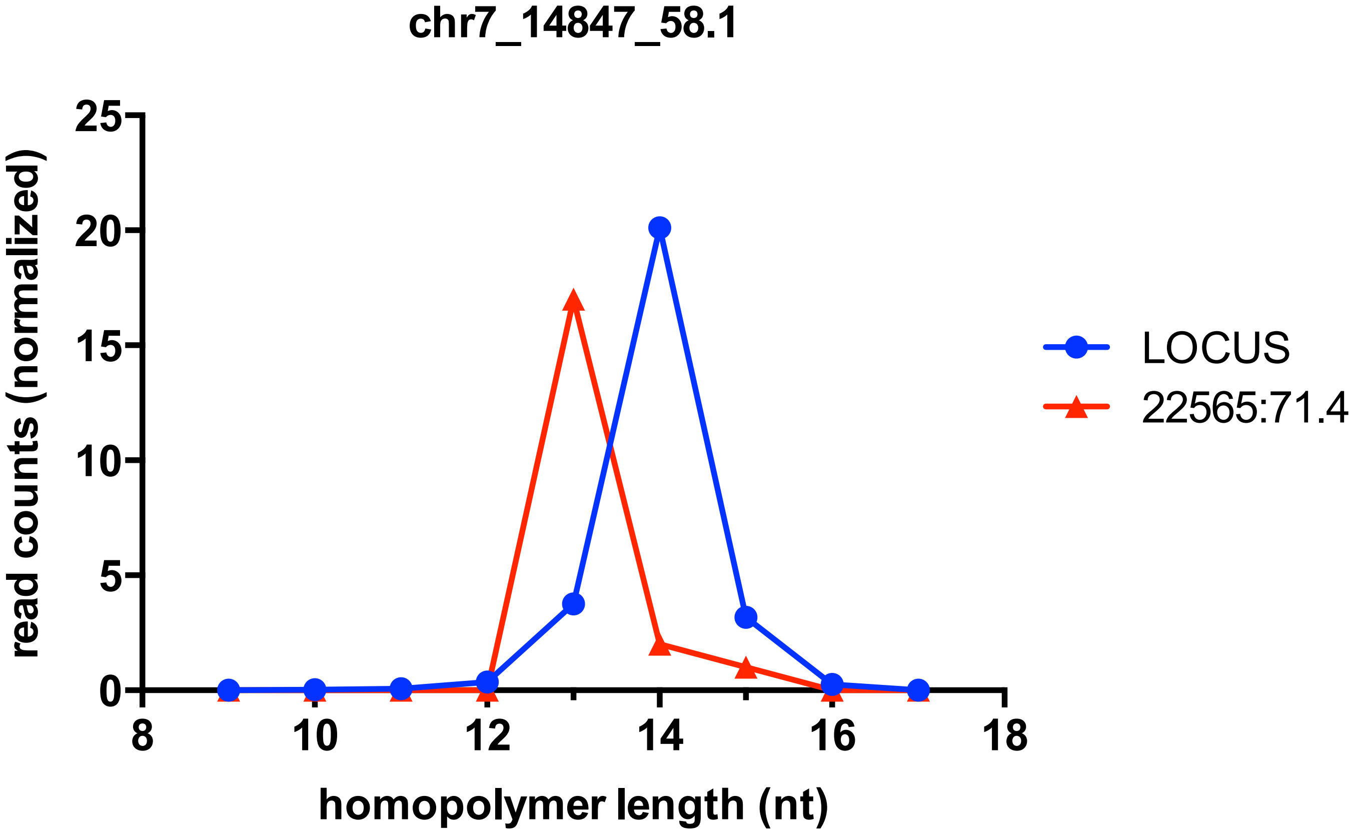
chr7_14847_58.1
read counts (normalized)
0
5
10
15
20
25
8
10
12
14
16
18
homopolymer length (nt)
LOCUS
22565:71.4

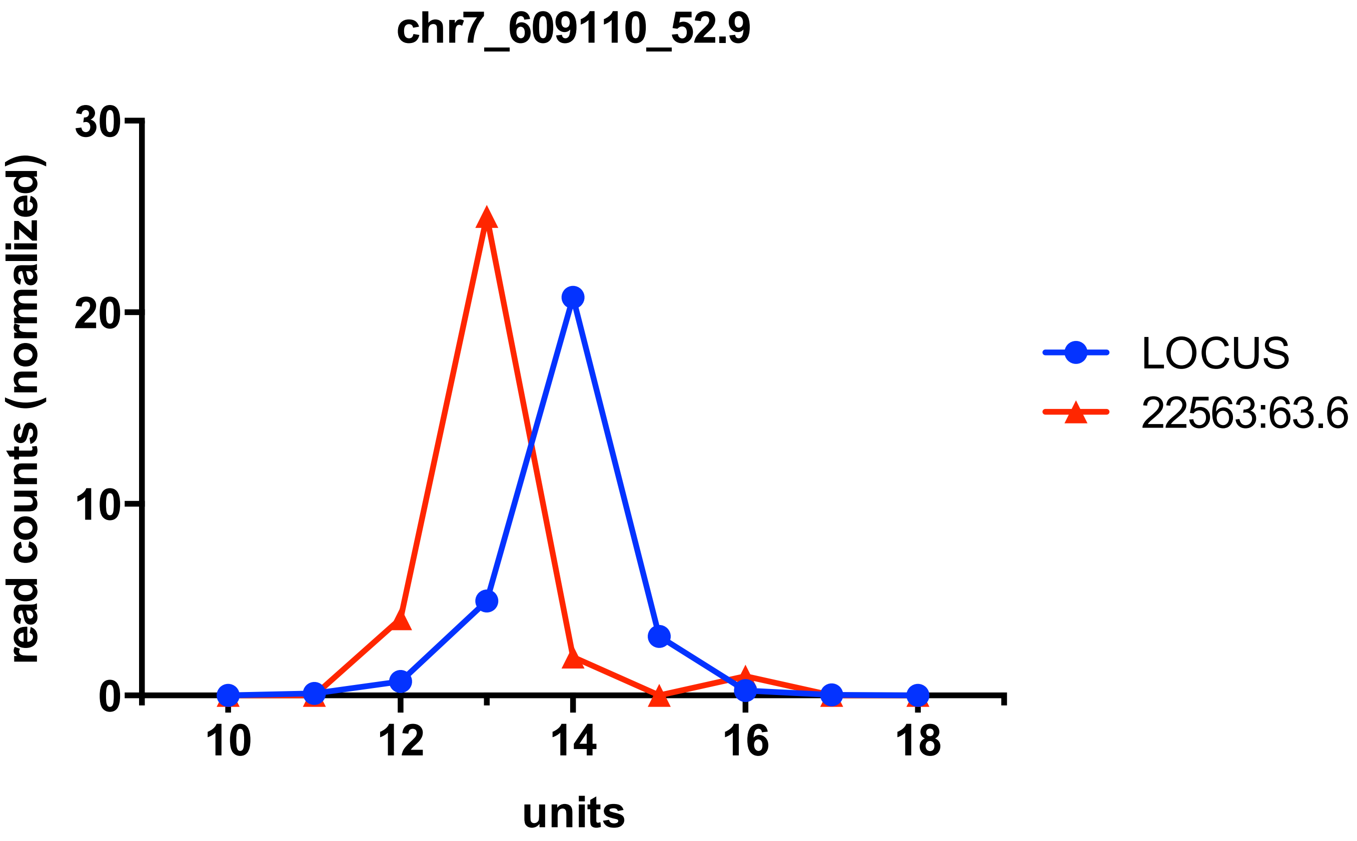

chr7_609110_52.9
30
20
10
0
10
12
14
16
18
units
read counts (normalized)
LOCUS
22563:63.6

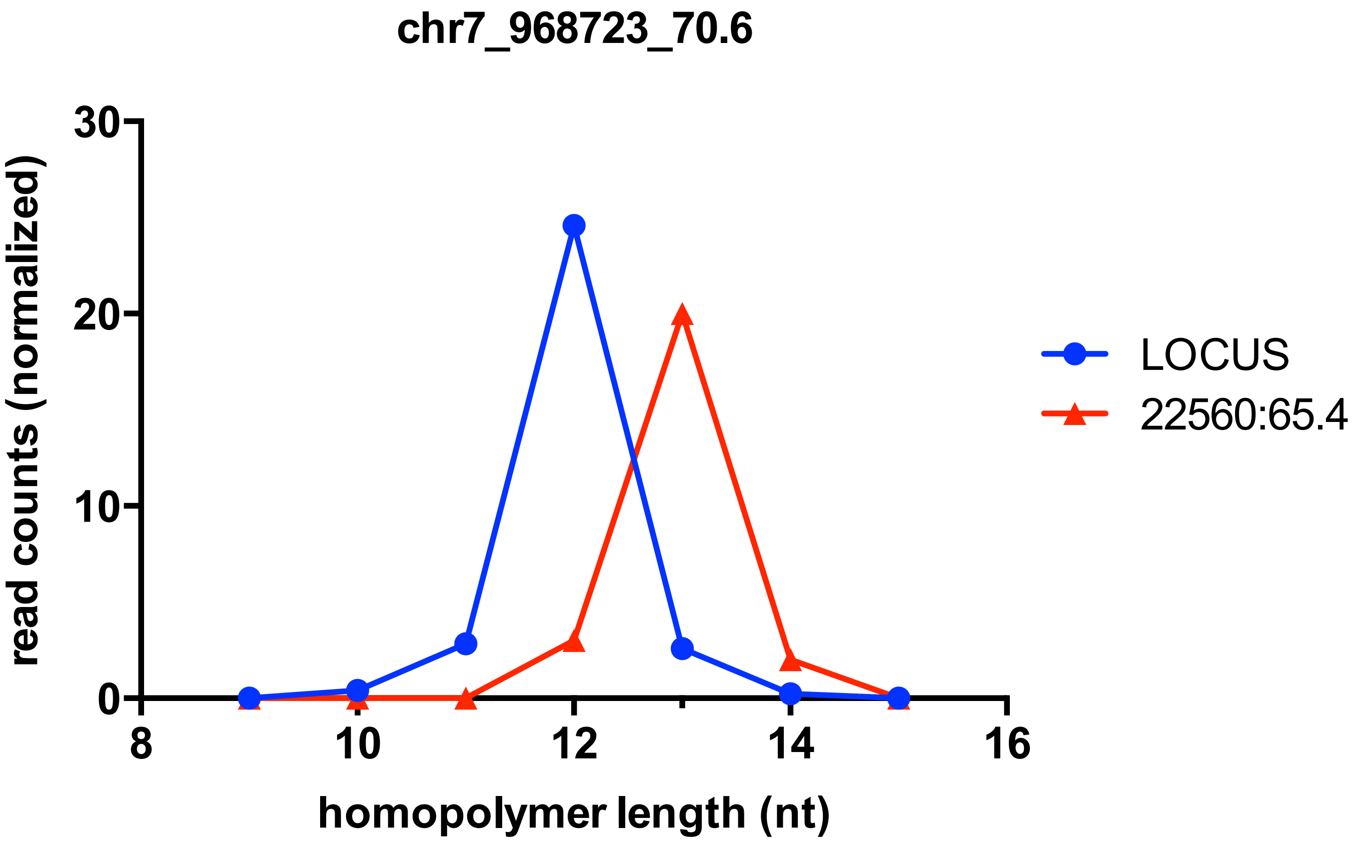

chr7_968723_70.6
30
20
10
0
8
10
12
14
16
read counts (normalized)
homopolymer length (nt)
LOCUS
22560:65.4

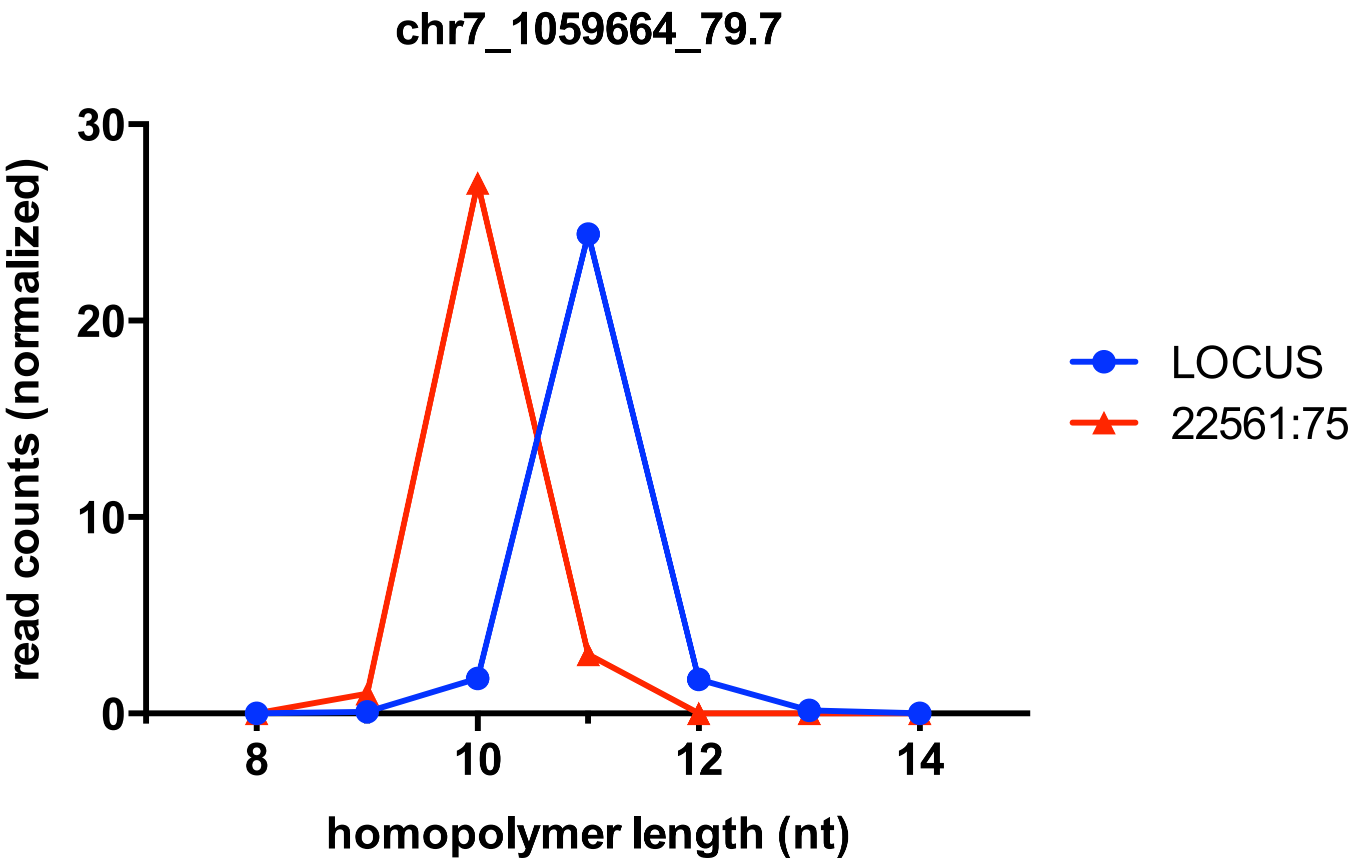
chr7_1059664_79.7
30
20
10
0
8
10
12
14
read counts (normalized)
homopolymer length (nt)
LOCUS
22561:75

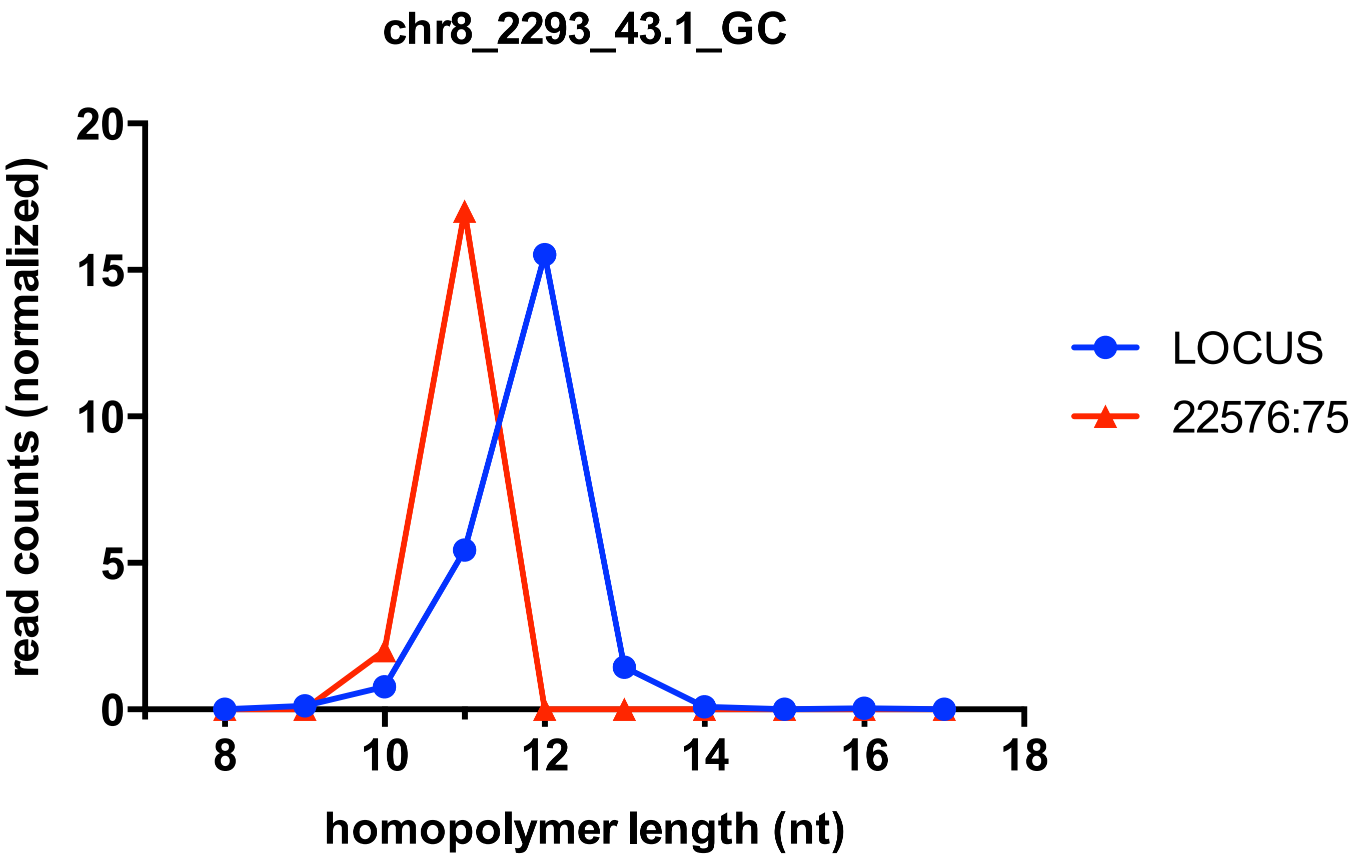
chr8_2293_43.1_GC
read counts (normalized)
0
5
10
15
20
8
10
12
14
16
18
homopolymer length (nt)
LOCUS
22576:75

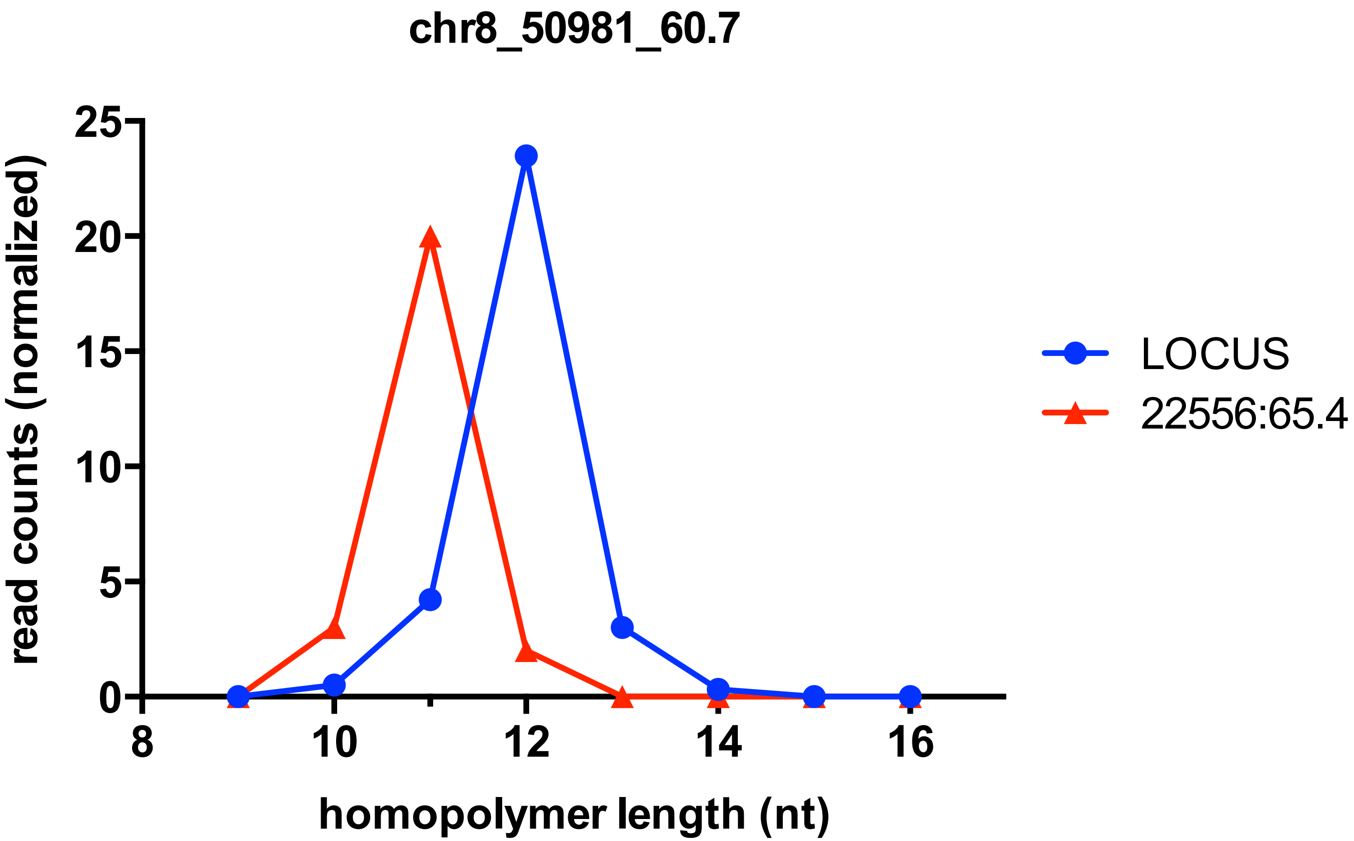
chr8_50981_60.7
read counts (normalized)
25
20
15
10
5
0
8
10
12
14
16
homopolymer length (nt)
LOCUS
22556:65.4

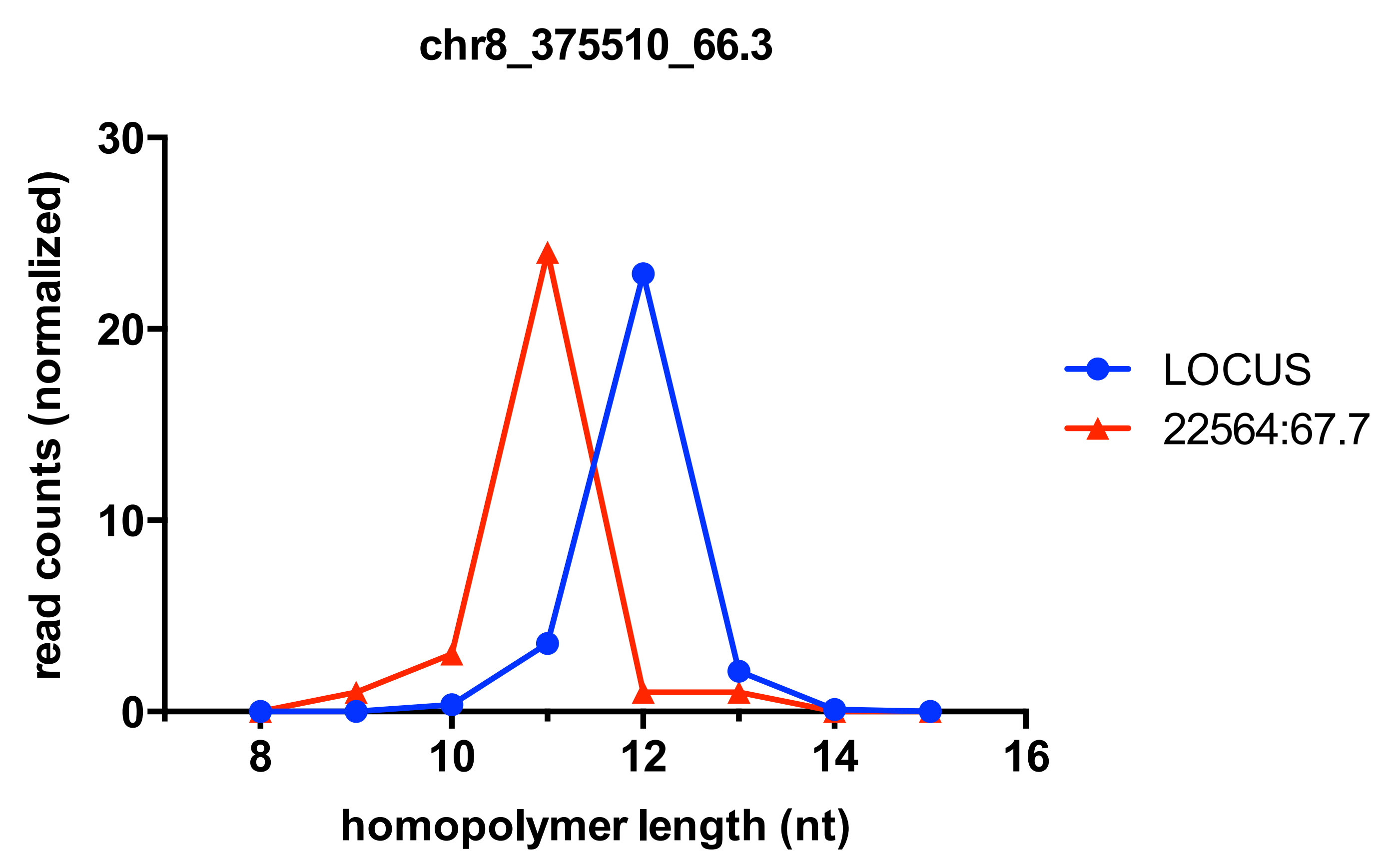

chr8_375510_66.3
read counts (normalized)
30
20
10
0
8
10
12
14
16
homopolymer length (nt)
LOCUS
22564:67.7

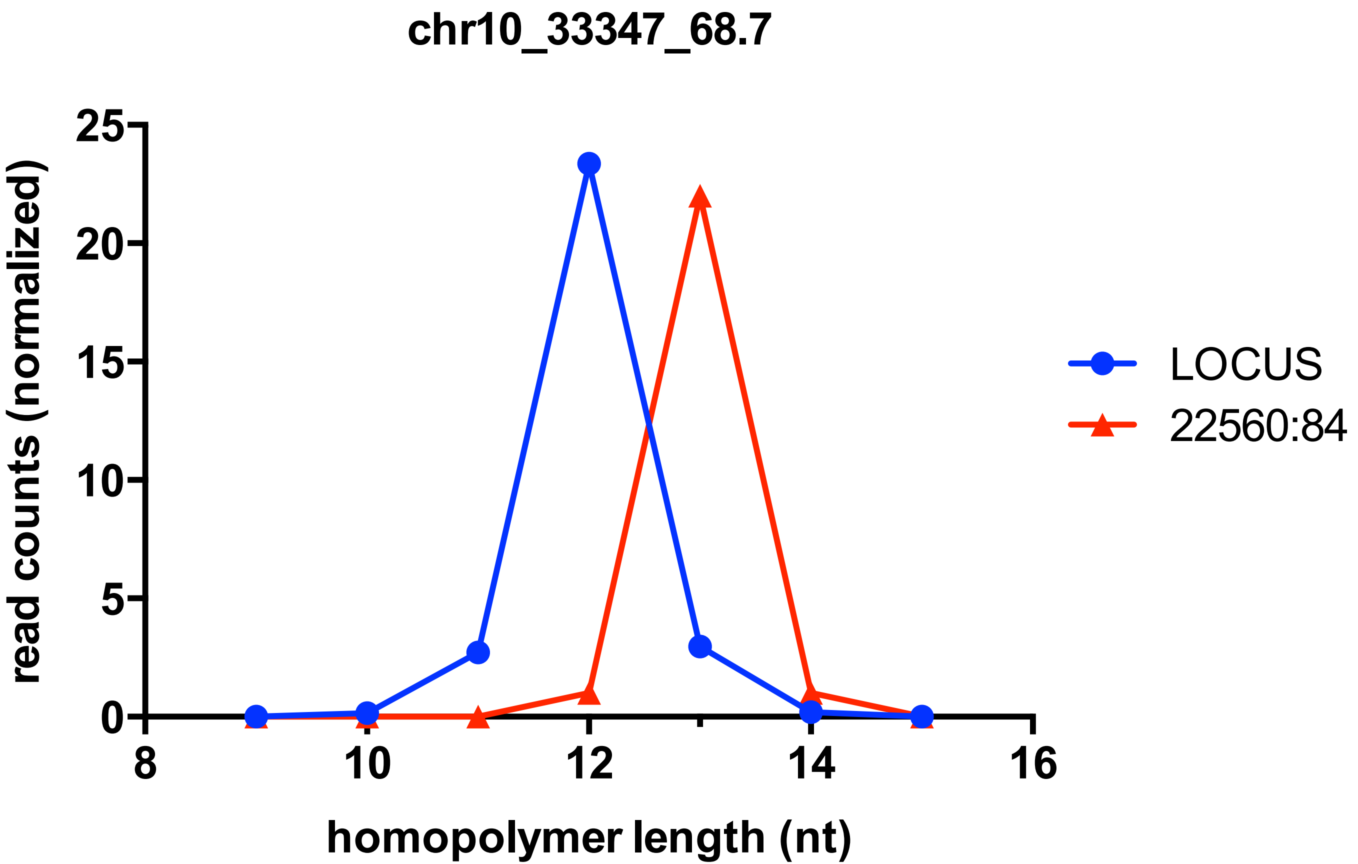
chr10_33347_68.7
read counts (normalized)
25
20
15
10
5
0
8
10
12
14
16
homopolymer length (nt)
LOCUS
22560:84

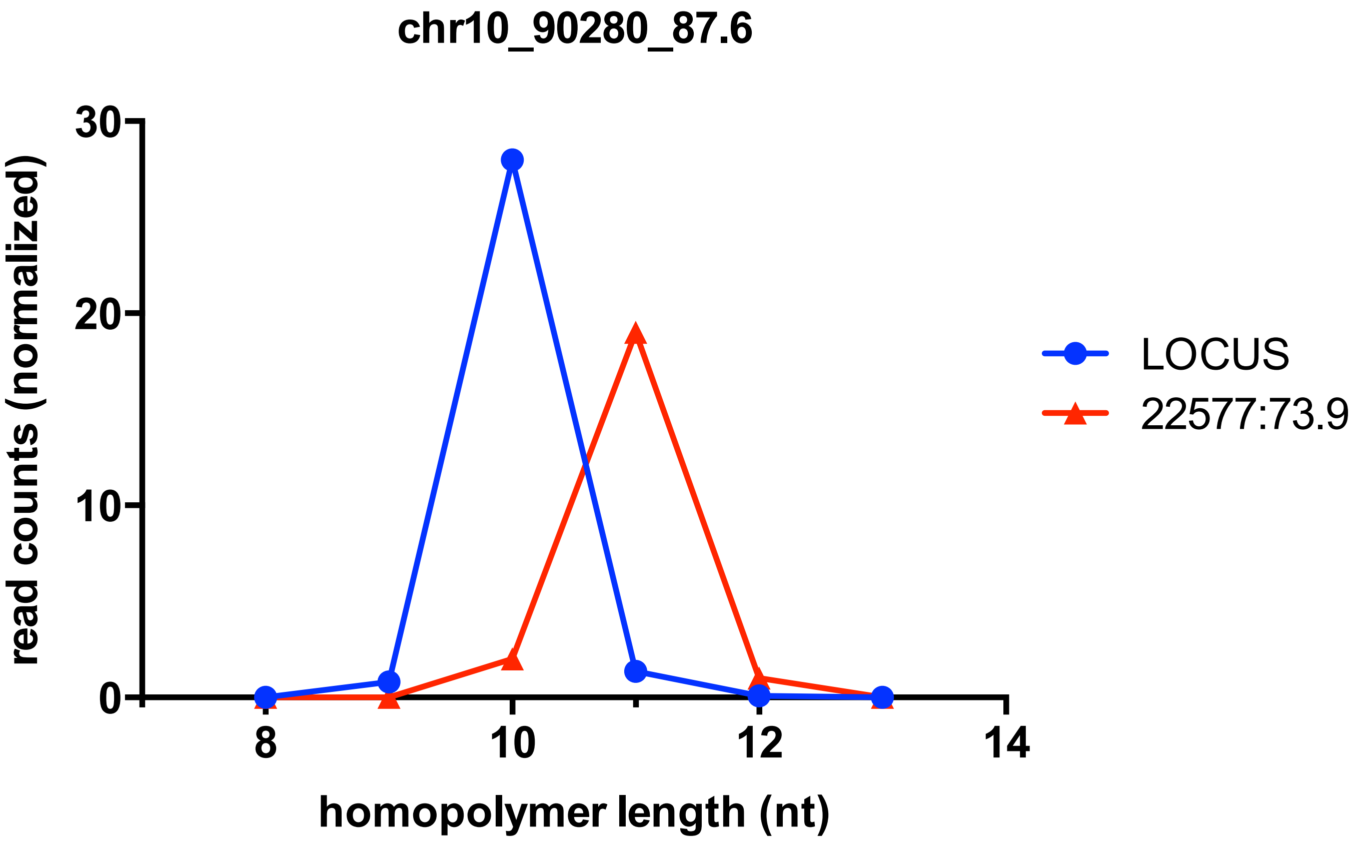

chr10_90280_87.6
read counts (normalized)
30
20
10
0
8
10
12
14
homopolymer length (nt)
LOCUS
22577:73.9

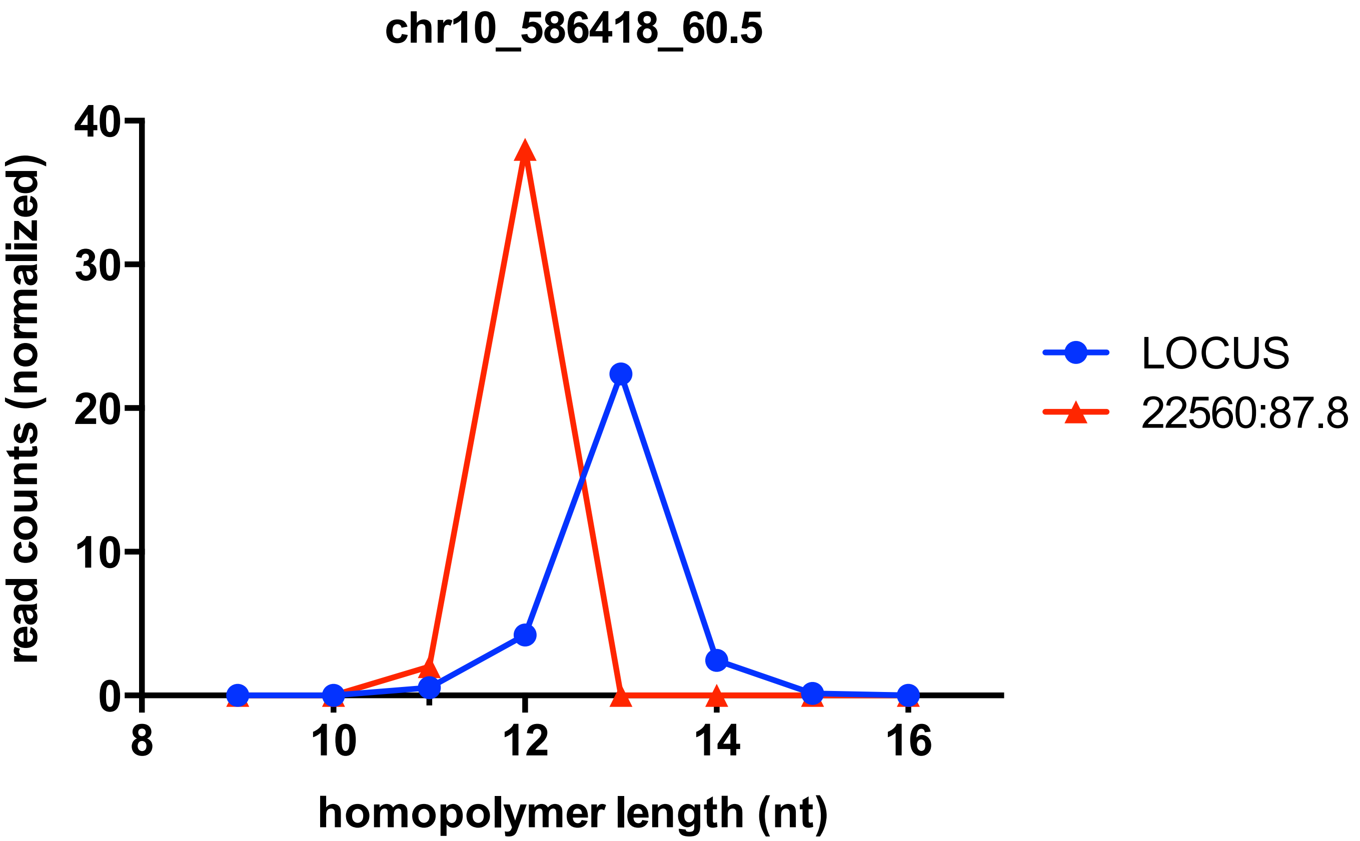
chr10_586418_60.5
read counts (normalized)
40
30
20
10
0
8
10
12
14
16
homopolymer length (nt)
LOCUS
22560:87.8

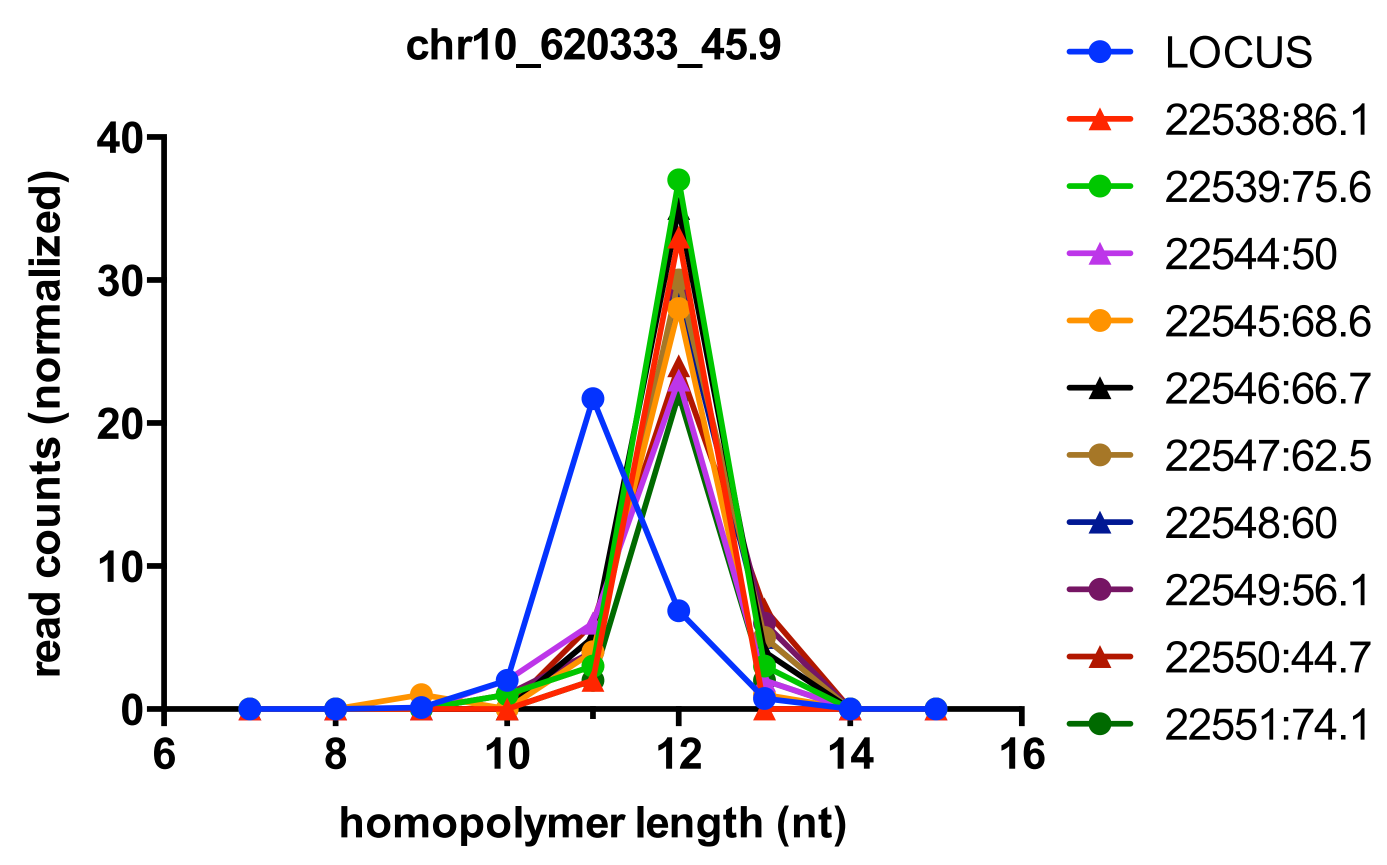

chr10_620333_45.9
read counts (normalized)
homopolymer length (nt)
0
10
20
30
40
6
8
10
12
14
16
LOCUS
22538:86.1
22539:75.6
22544:50
22545:68.6
22546:66.7
22547:62.5
22548:60
22549:56.1
22550:44.7
22551:74.1

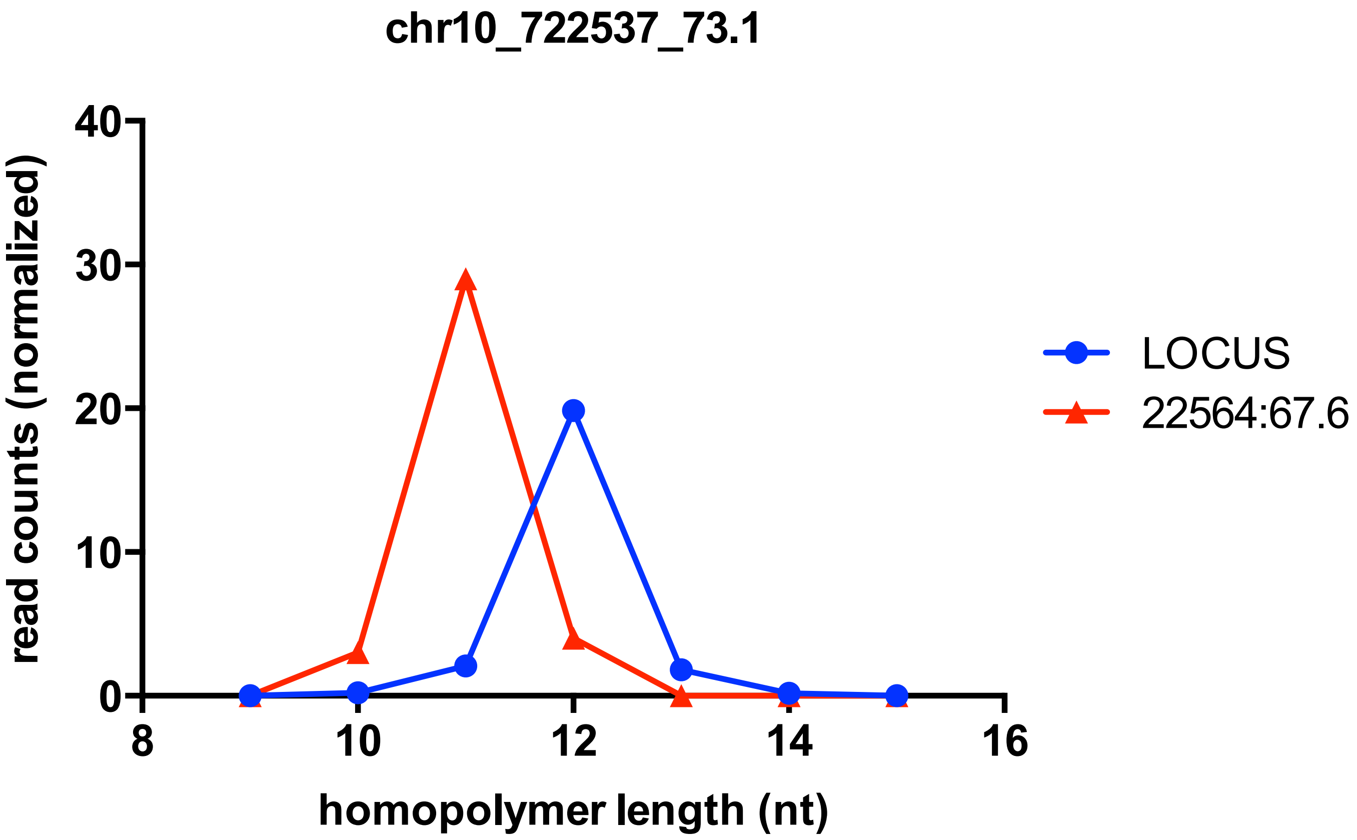
chr10_722537_73.1
read counts (normalized)
40
30
20
10
0
8
10
12
14
16
homopolymer length (nt)
LOCUS
22564:67.6

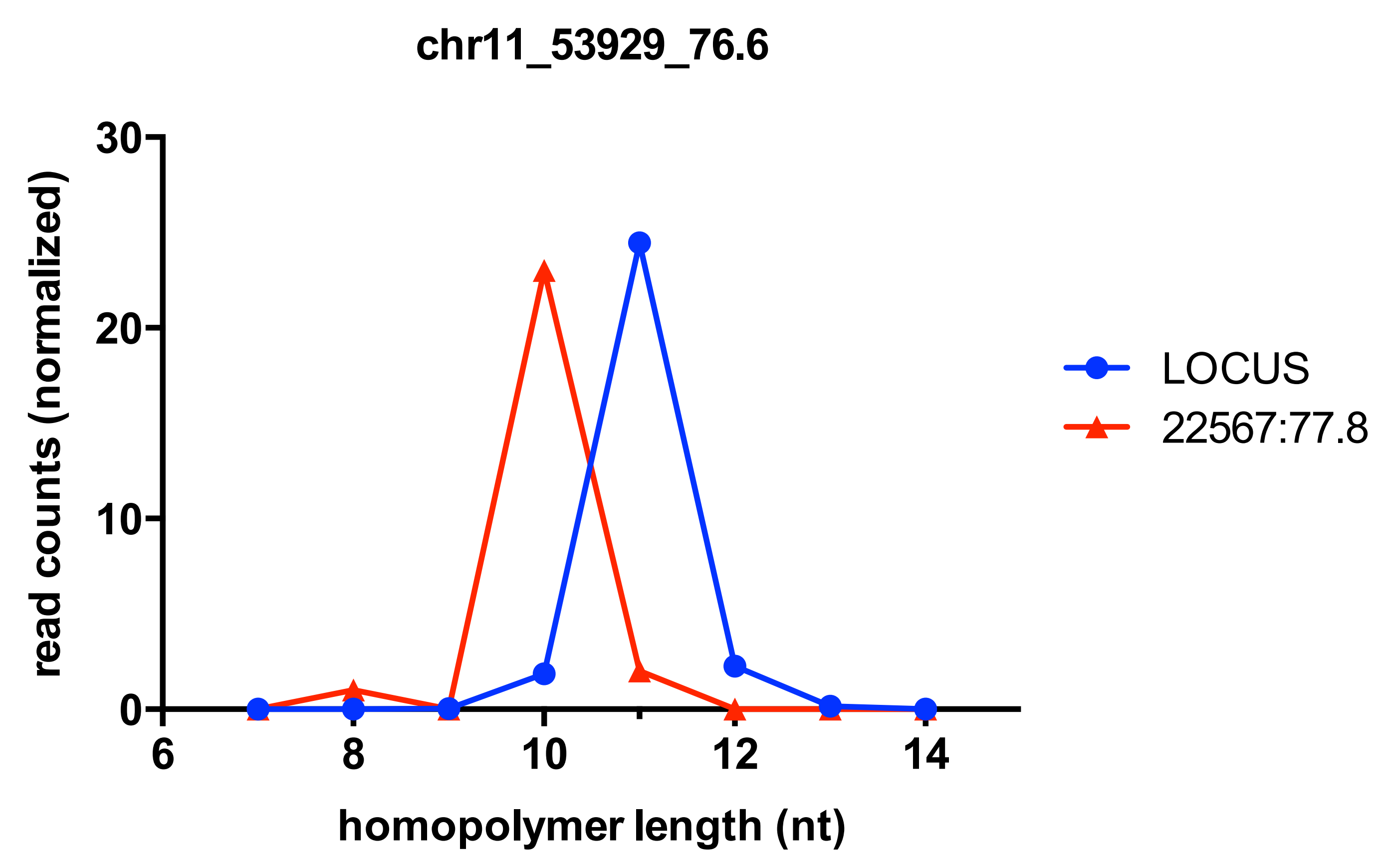

chr11_53929_76.6
read counts (normalized)
30
20
10
0
6
8
10
12
14
homopolymer length (nt)
LOCUS
22567:77.8

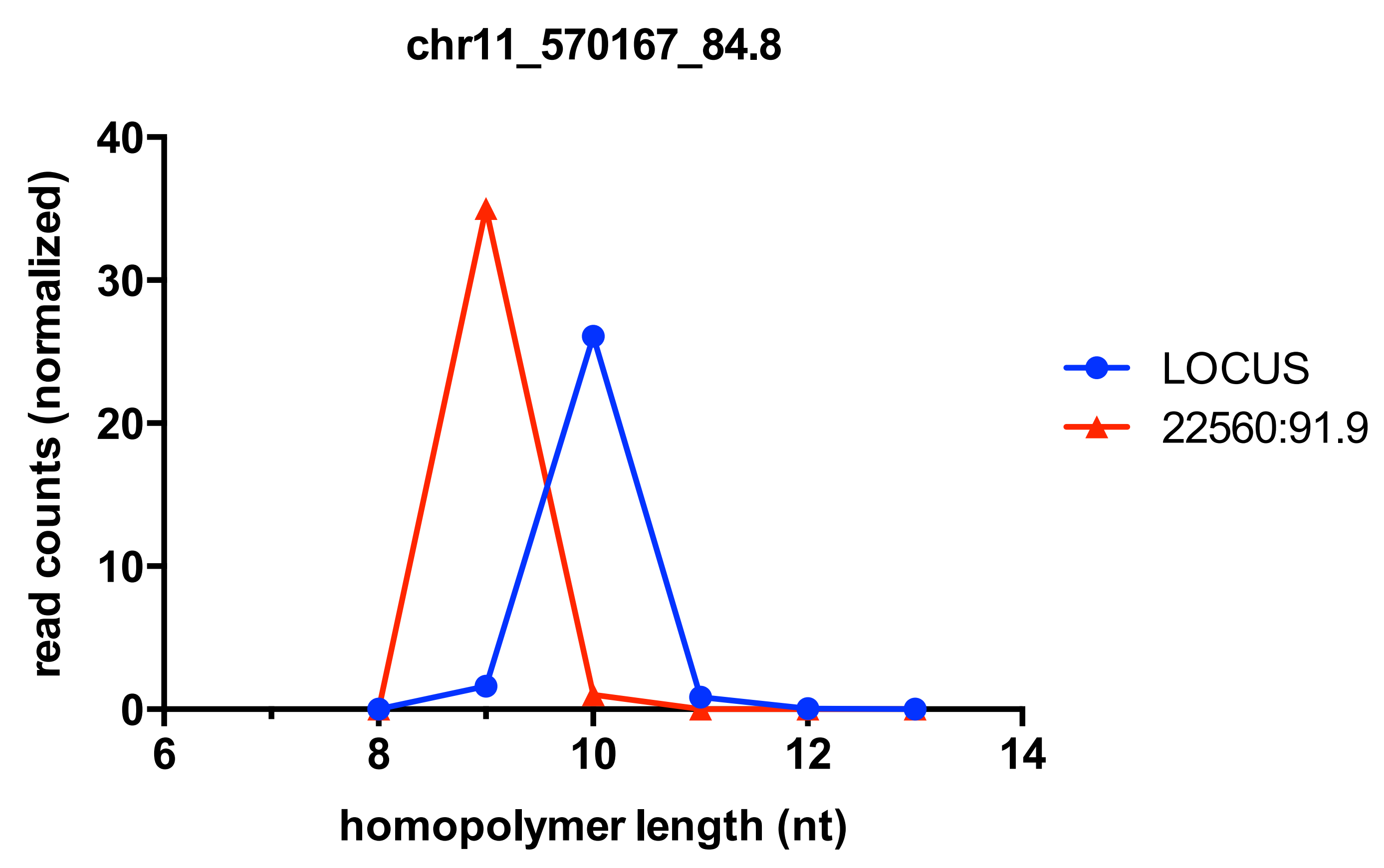
chr11_570167_84.8
read counts (normalized)
40
30
20
10
0
6
8
10
12
14
homopolymer length (nt)
LOCUS
22560:91.9

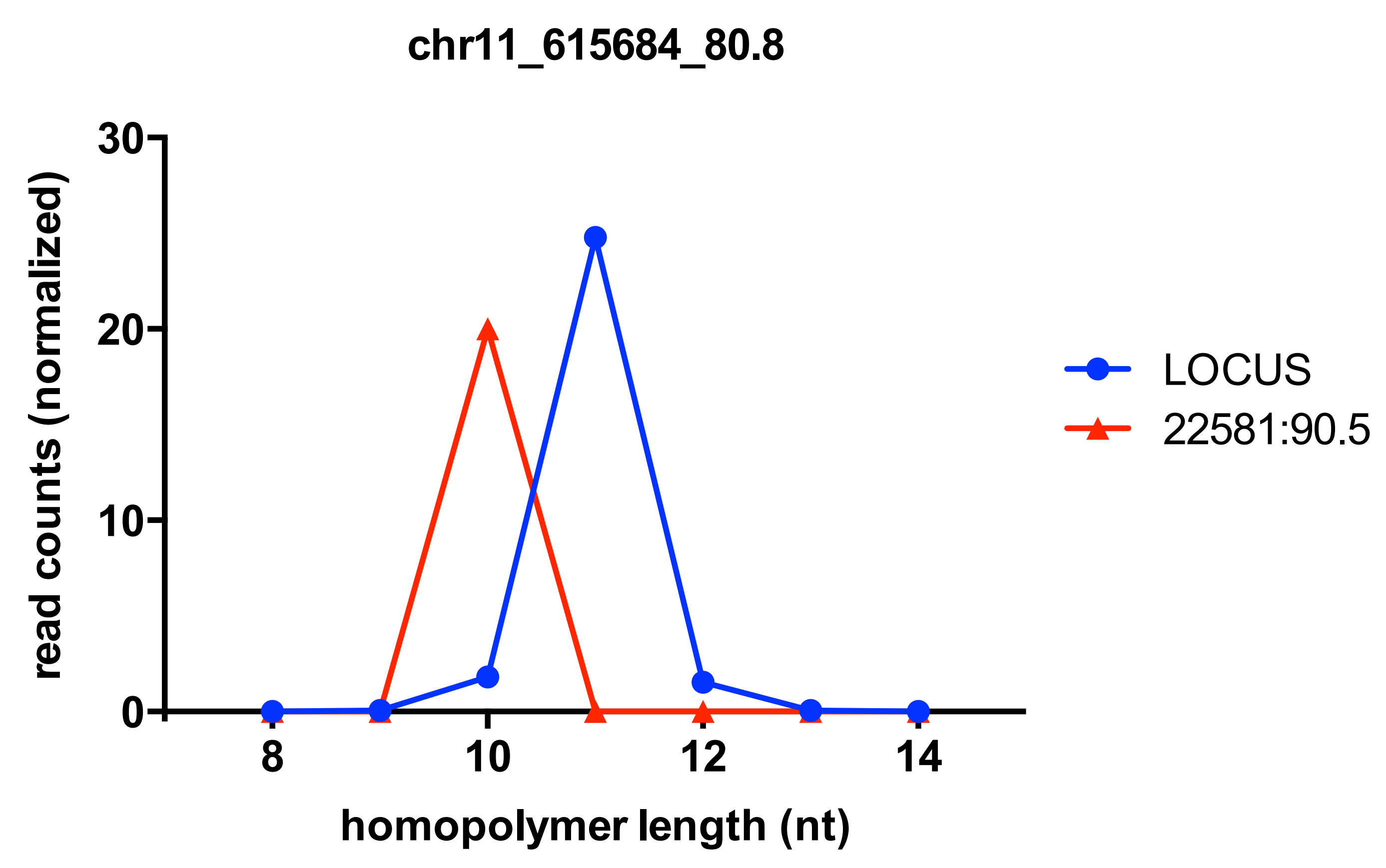
chr11_615684_80.8
read counts (normalized)
30
20
10
0
8
10
12
14
homopolymer length (nt)
LOCUS
22581:90.5

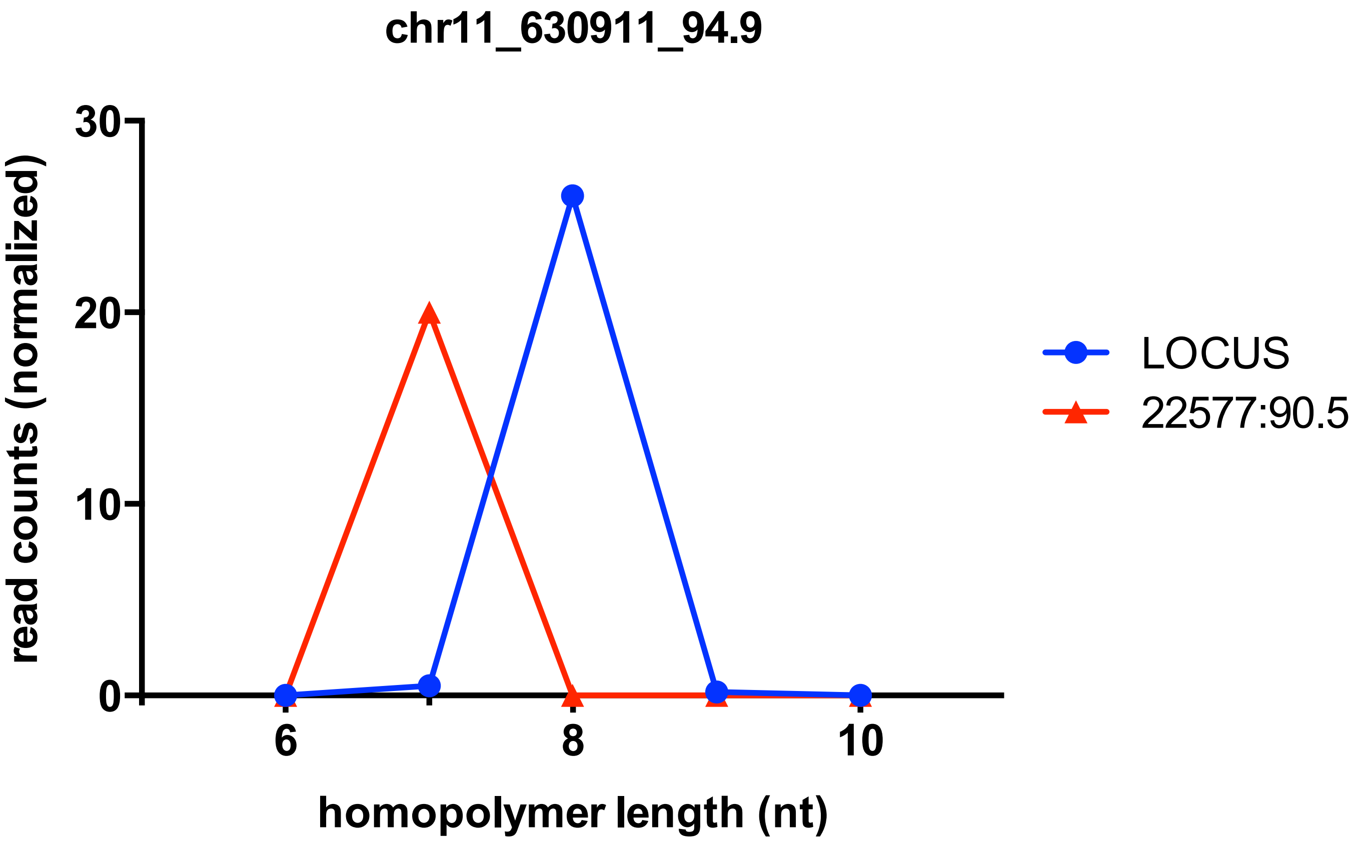

chr11_630911_94.9
read counts (normalized)
30
20
10
0
6
8
10
homopolymer length (nt)
LOCUS
22577:90.5

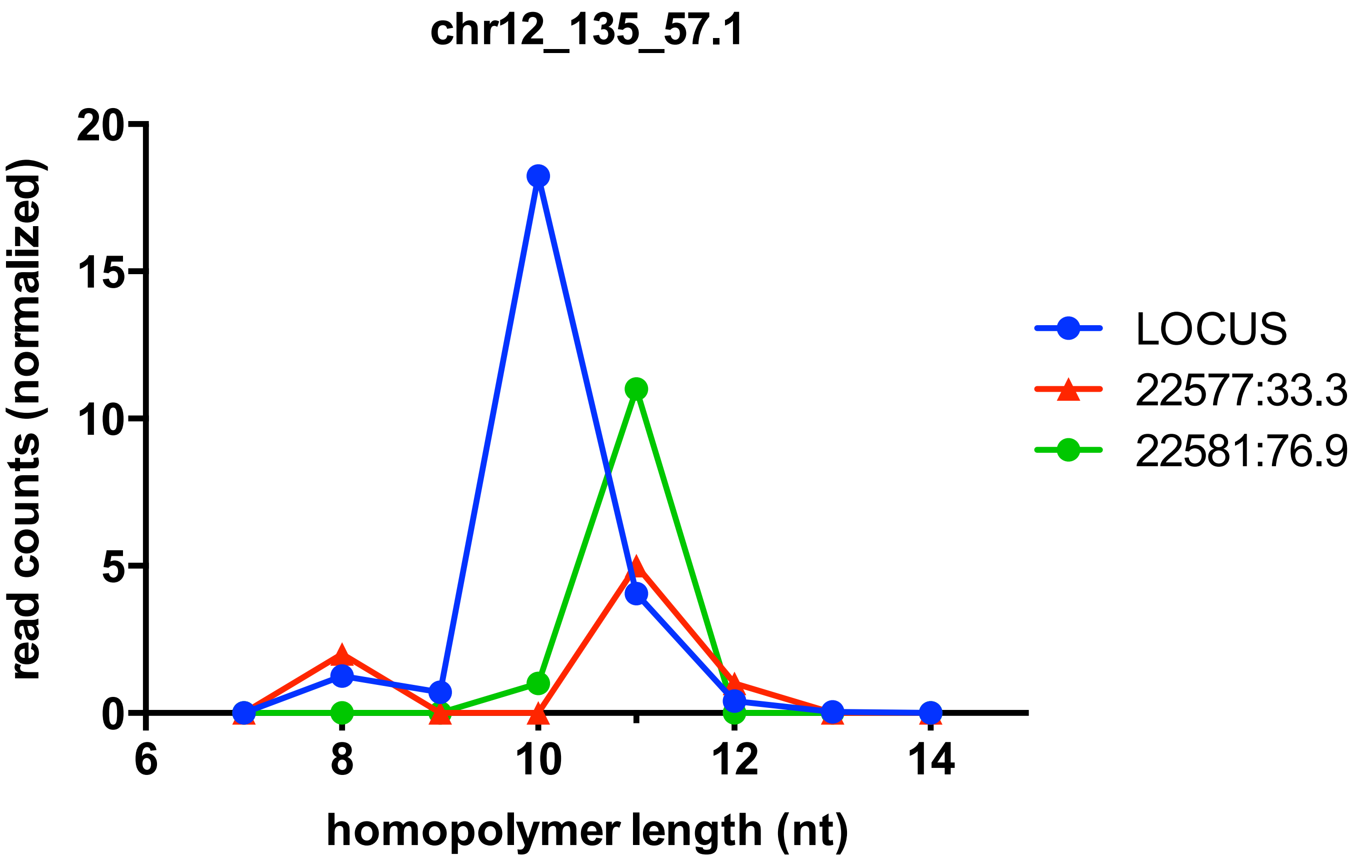
chr12_135_57.1
20
15
10
5
0
6
8
10
12
14
read counts (normalized)
homopolymer length (nt)
LOCUS
22577:33.3
22581:76.9

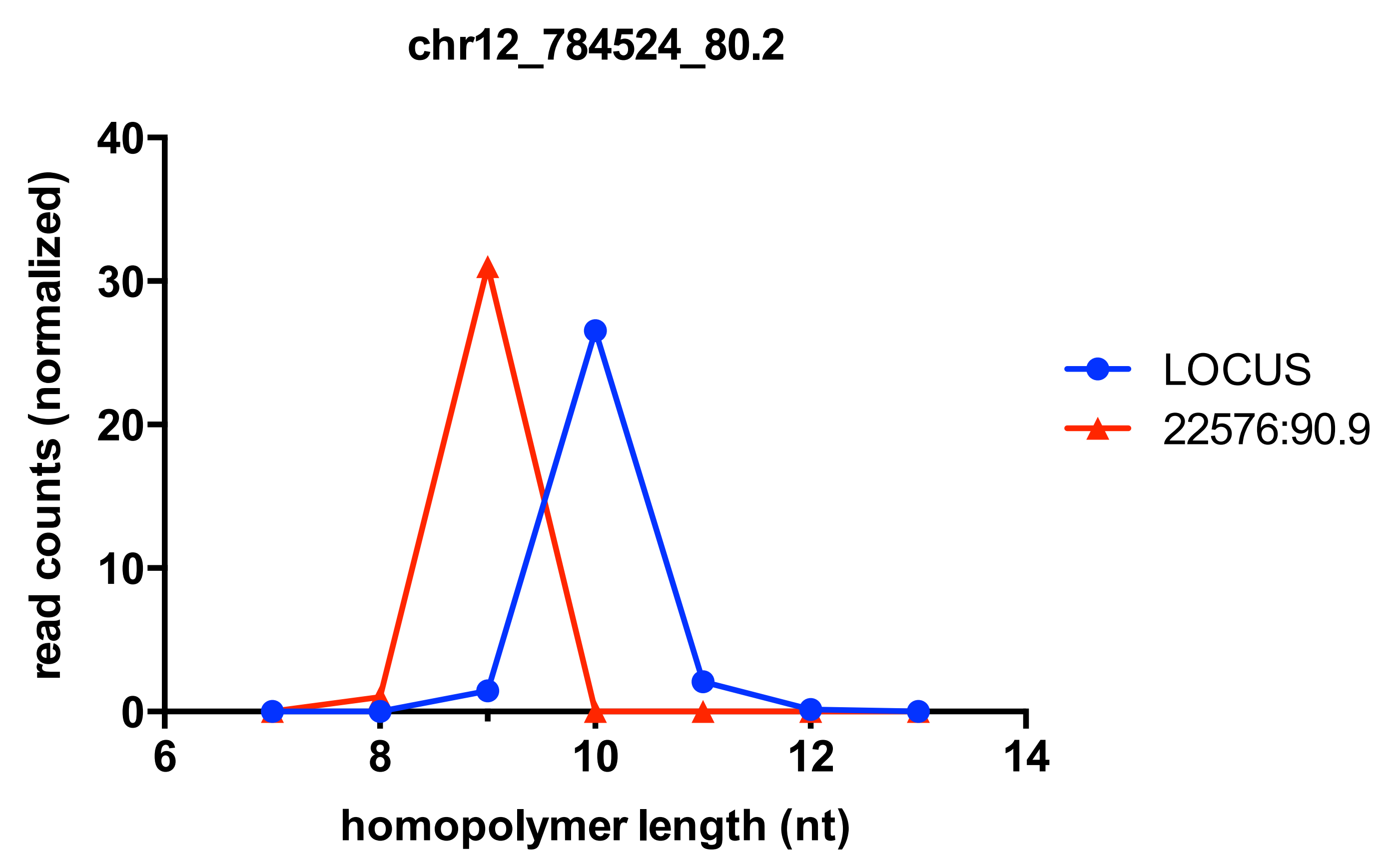
chr12_784524_80.2
read counts (normalized)
homopolymer length (nt)
0
10
20
30
40
6
8
10
12
14
LOCUS
22576:90.9

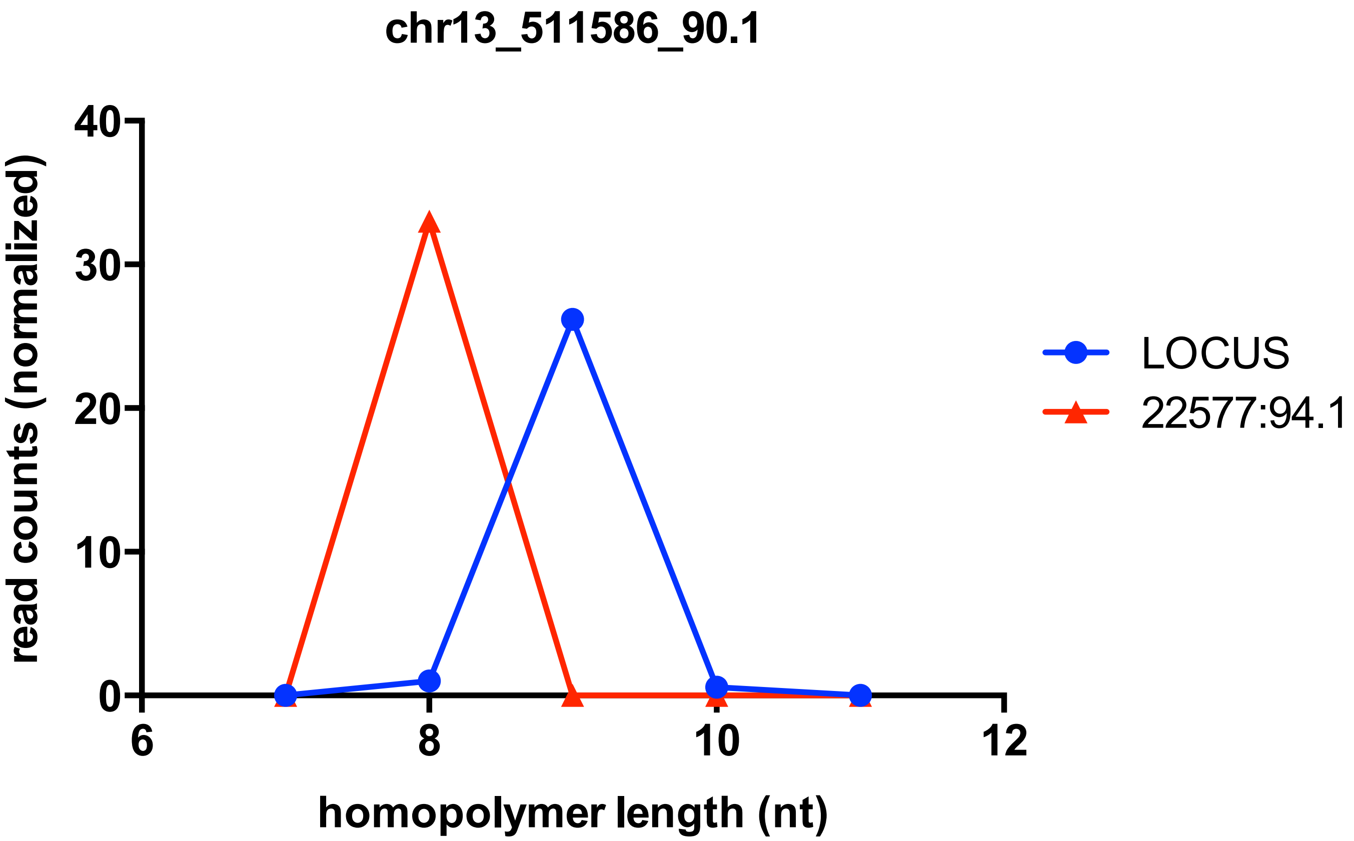

chr13_511586_90.1
read counts (normalized)
40
30
20
10
0
6
8
10
12
homopolymer length (nt)
LOCUS
22577:94.1

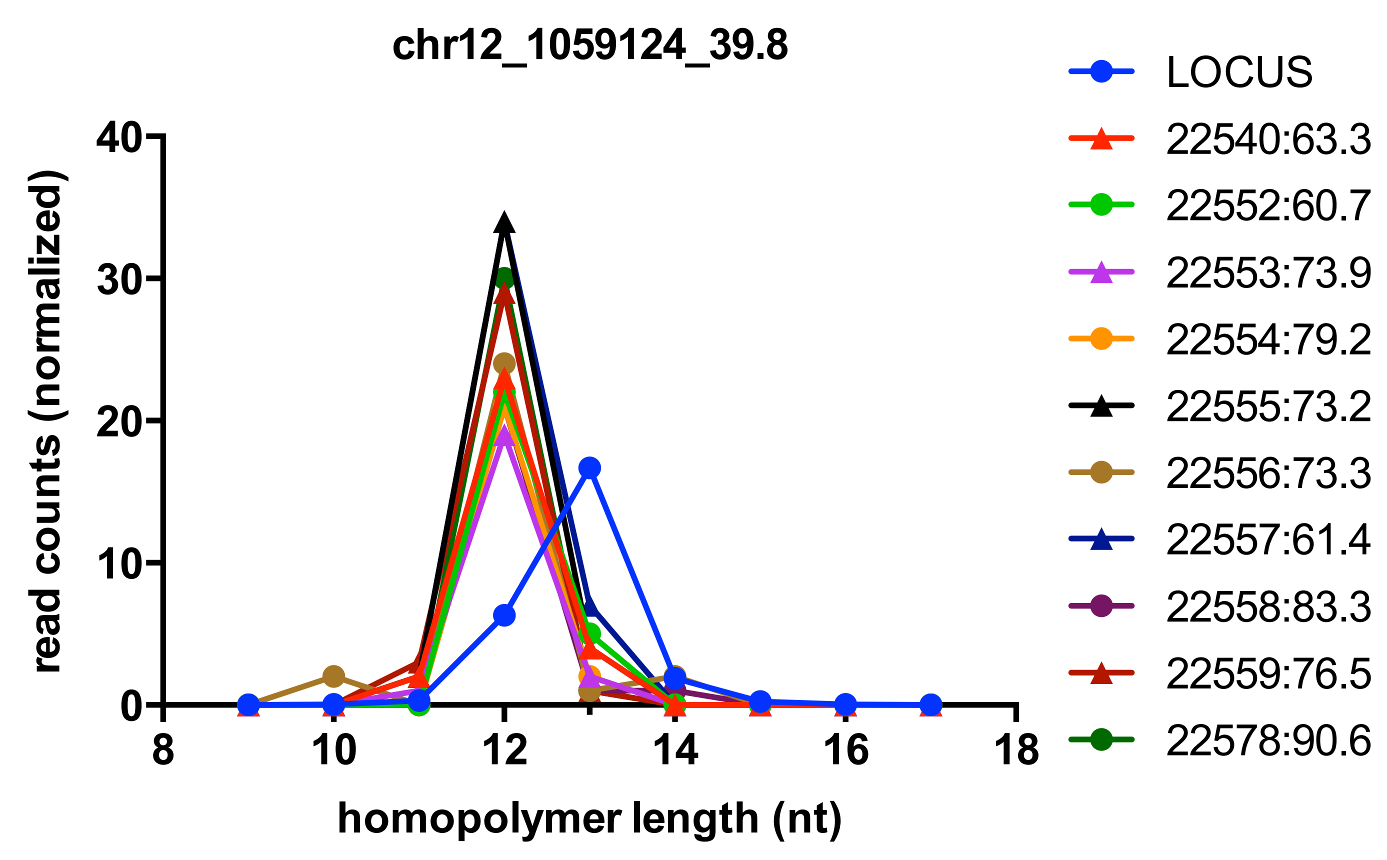
chr12_1059124_39.8
read counts (normalized)
homopolymer length (nt)
0
10
20
30
40
8
10
12
14
16
18
LOCUS
22540:63.3
22552:60.7
22553:73.9
22554:79.2
22555:73.2
22556:73.3
22557:61.4
22558:83.3
22559:76.5
22578:90.6

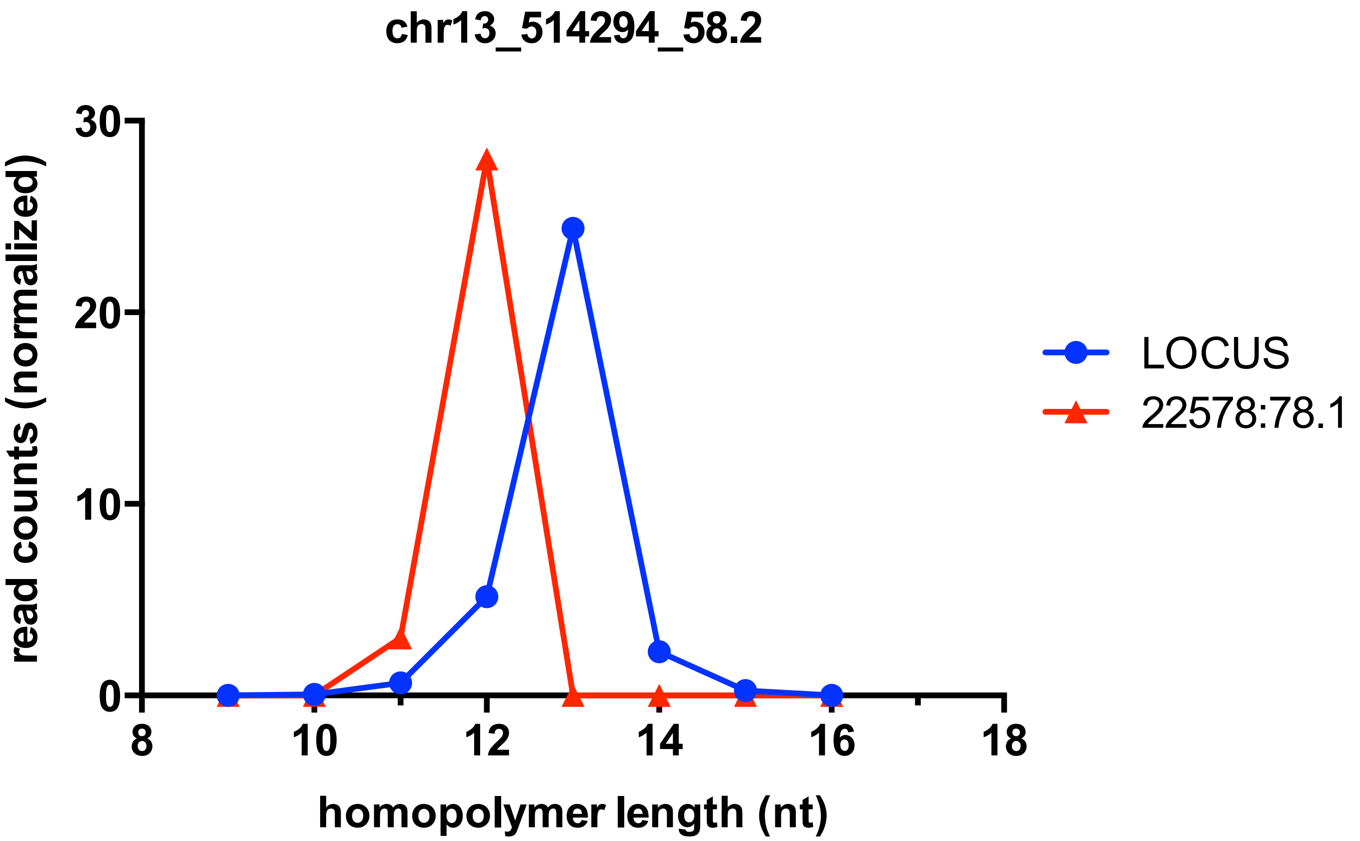
chr13_514294_58.2
read counts (normalized)
0
10
20
30
8
10
12
14
16
18
homopolymer length (nt)
LOCUS
22578:78.1

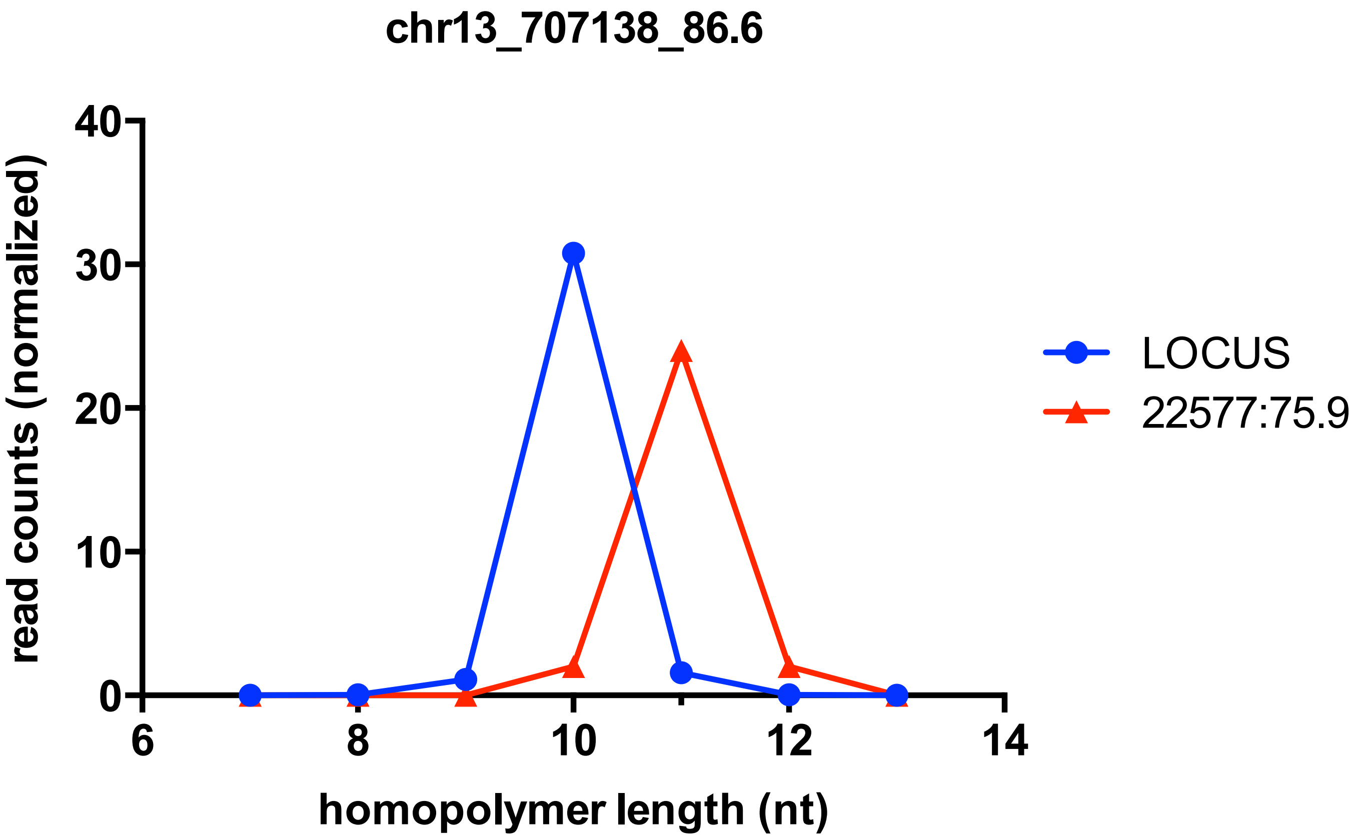
chr13_707138_86.6
read counts (normalized)
homopolymer length (nt)
0
10
20
30
40
6
8
10
12
14
LOCUS
22577:75.9

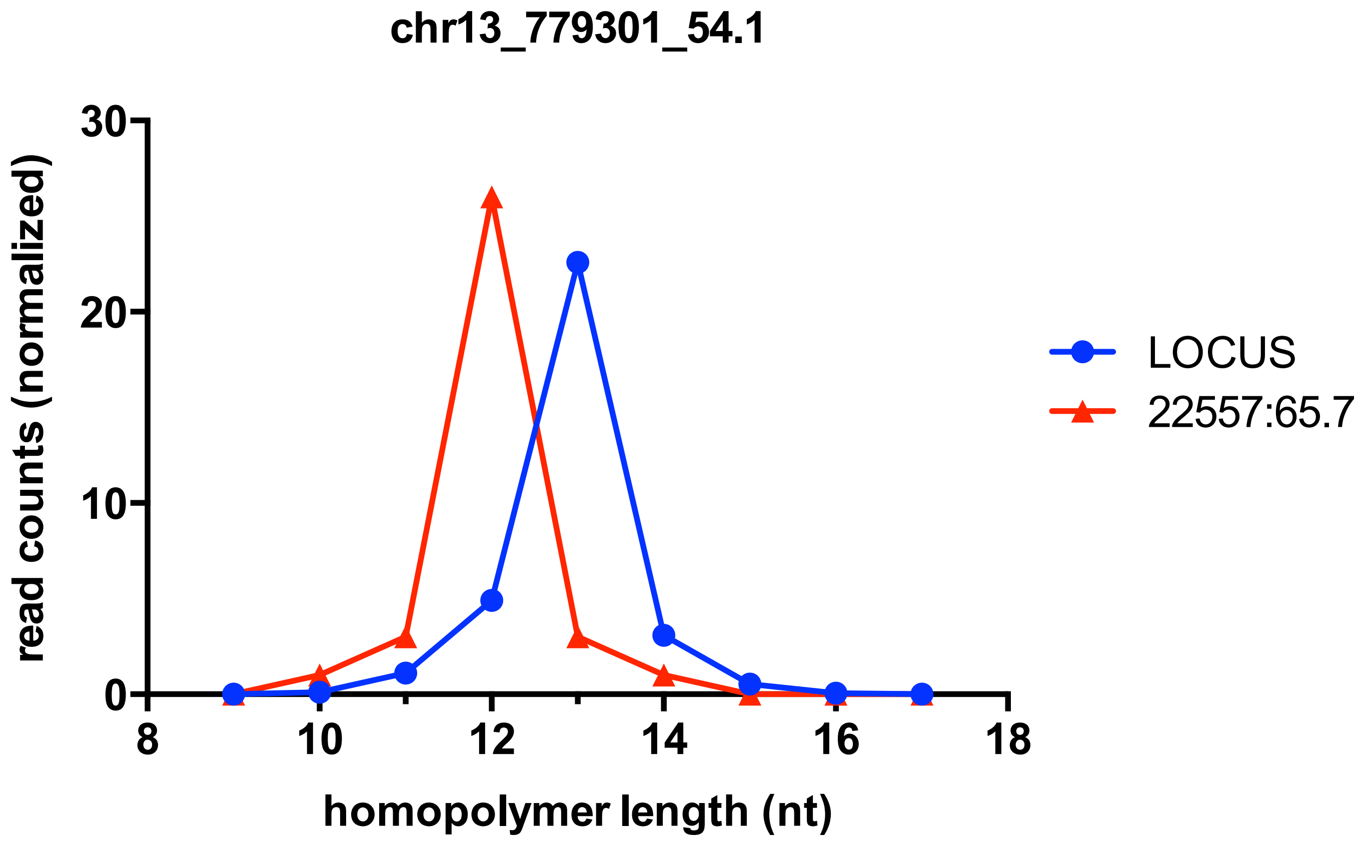

chr13_779301_54.1
read counts (normalized)
30
20
10
0
8
10
12
14
16
18
homopolymer length (nt)
LOCUS
22557:65.7

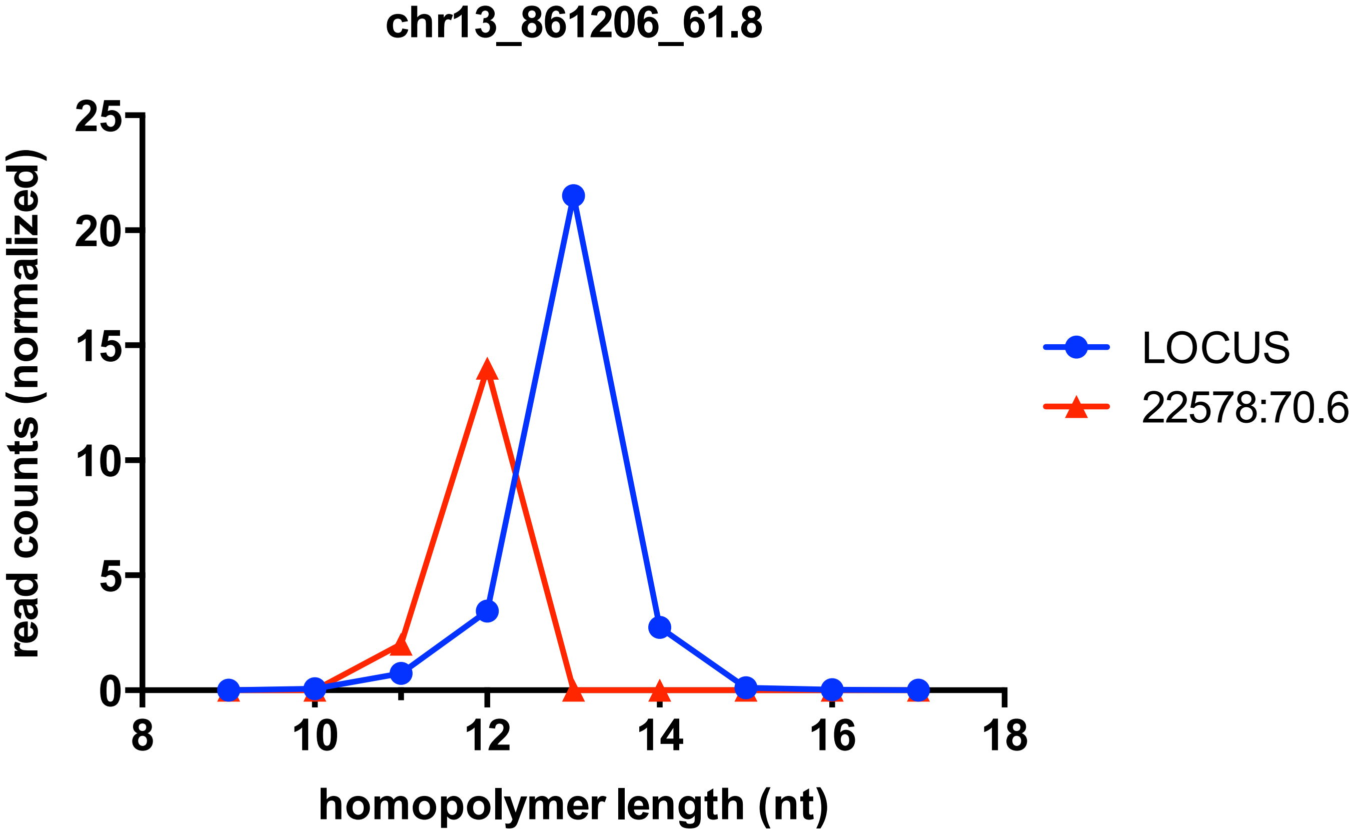
chr13_861206_61.8
25
20
15
10
5
0
read counts (normalized)
8
10
12
14
16
18
homopolymer length (nt)
LOCUS
22578:70.6

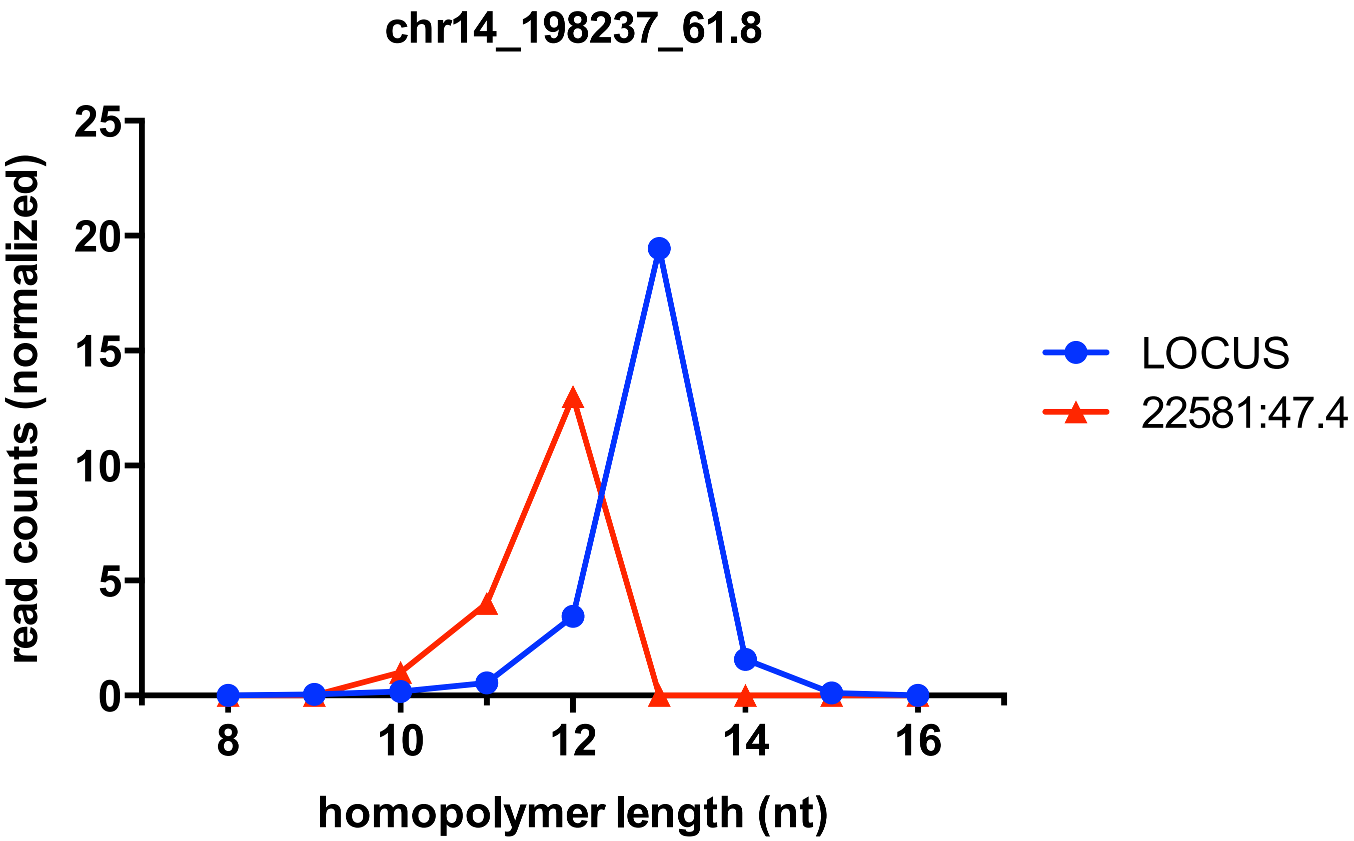

chr14_198237_61.8
read counts (normalized)
25
20
15
10
5
0
8
10
12
14
16
homopolymer length (nt)
LOCUS
22581:47.4

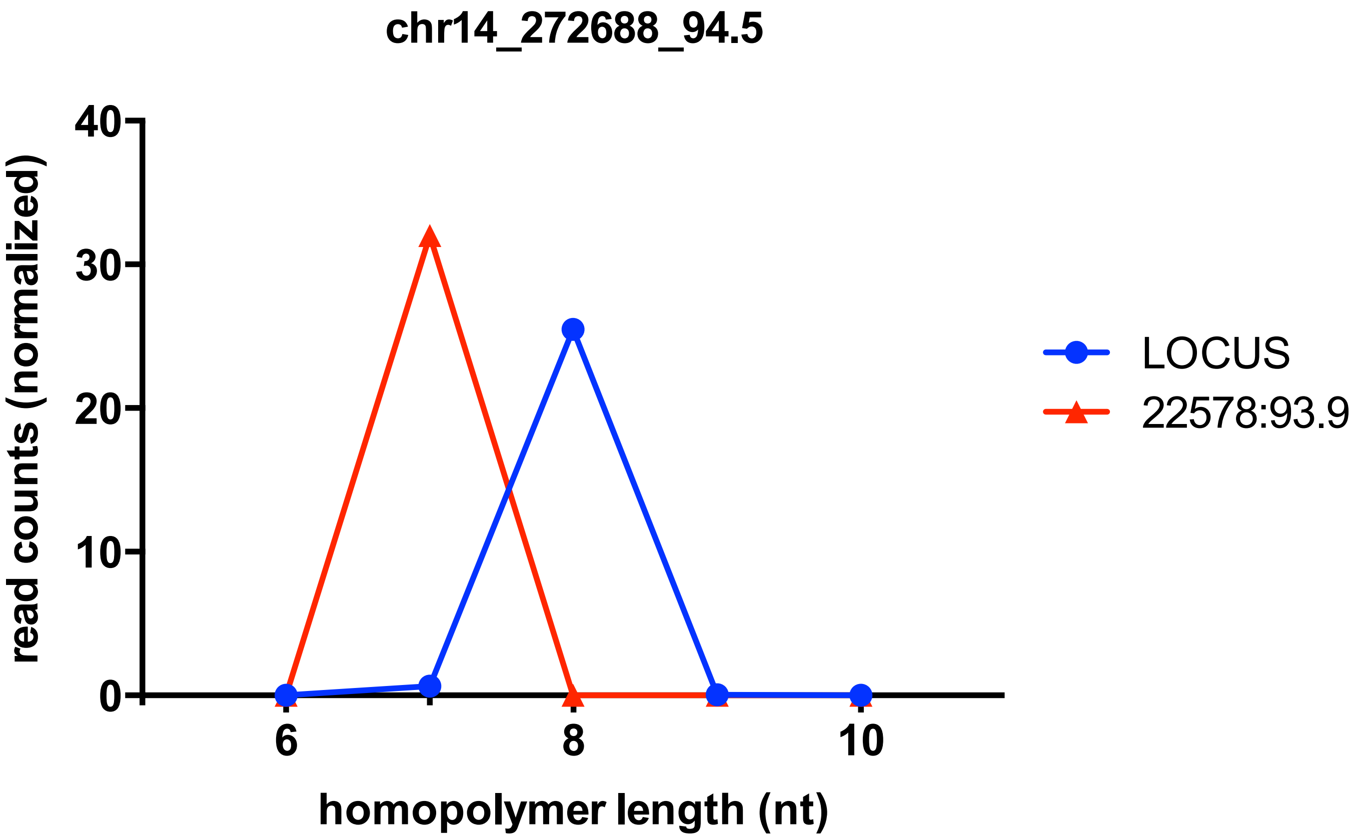
chr14_272688_94.5
read counts (normalized)
40
30
20
10
0
6
8
10
homopolymer length (nt)
LOCUS
22578:93.9

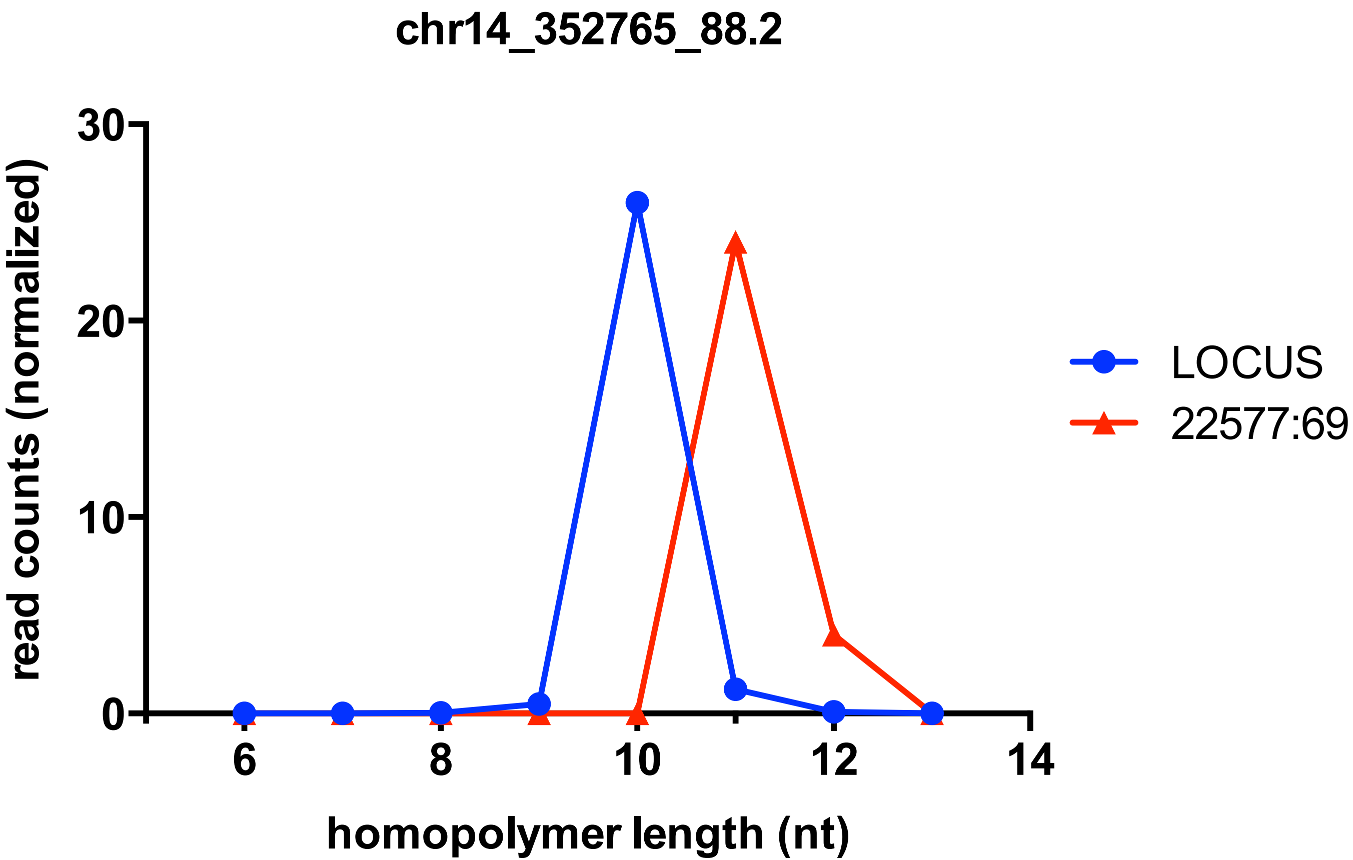

chr14_352765_88.2
read counts (normalized)
30
20
10
0
6
8
10
12
14
homopolymer length (nt)
LOCUS
22577:69

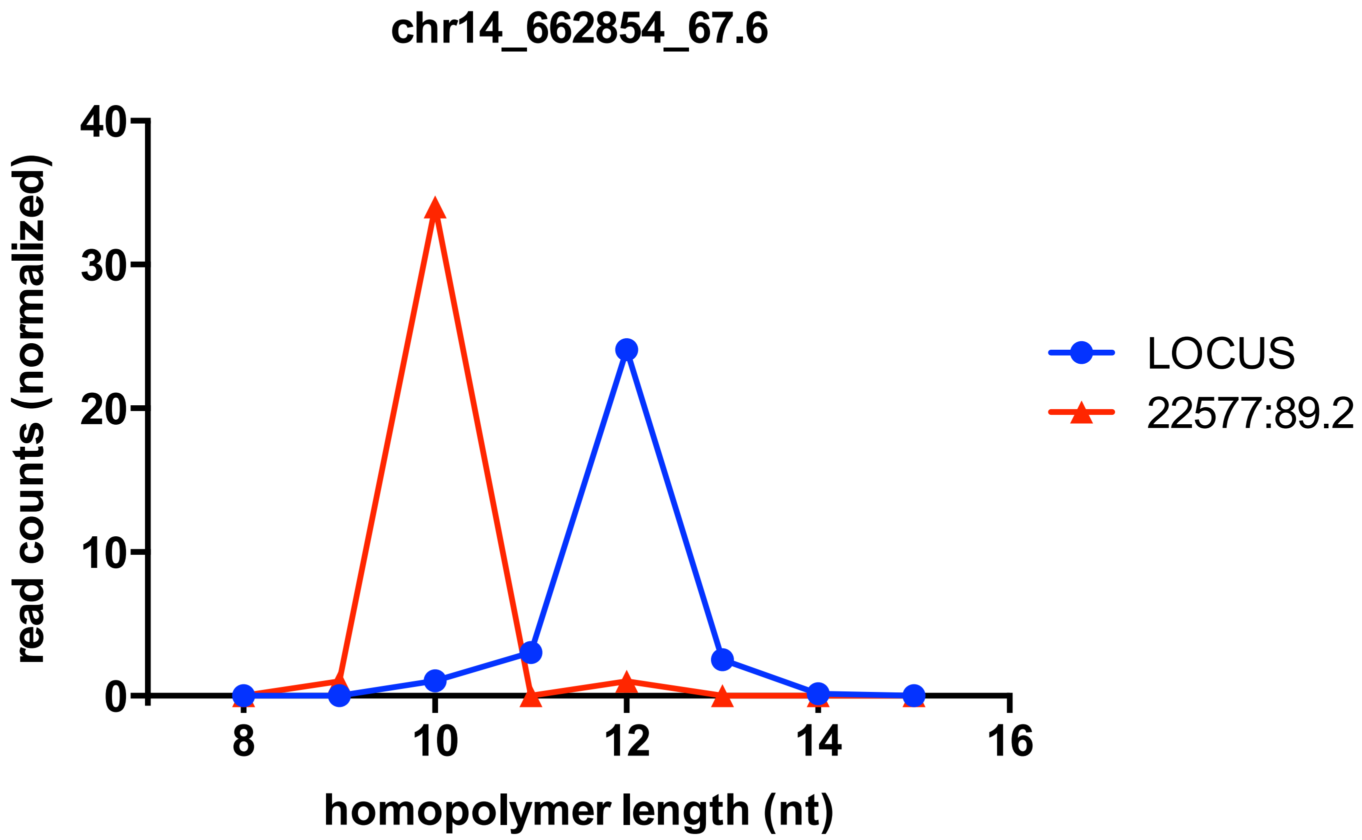
chr14_662854_67.6
read counts (normalized)
0
10
20
30
40
8
10
12
14
16
homopolymer length (nt)
LOCUS
22577:89.2

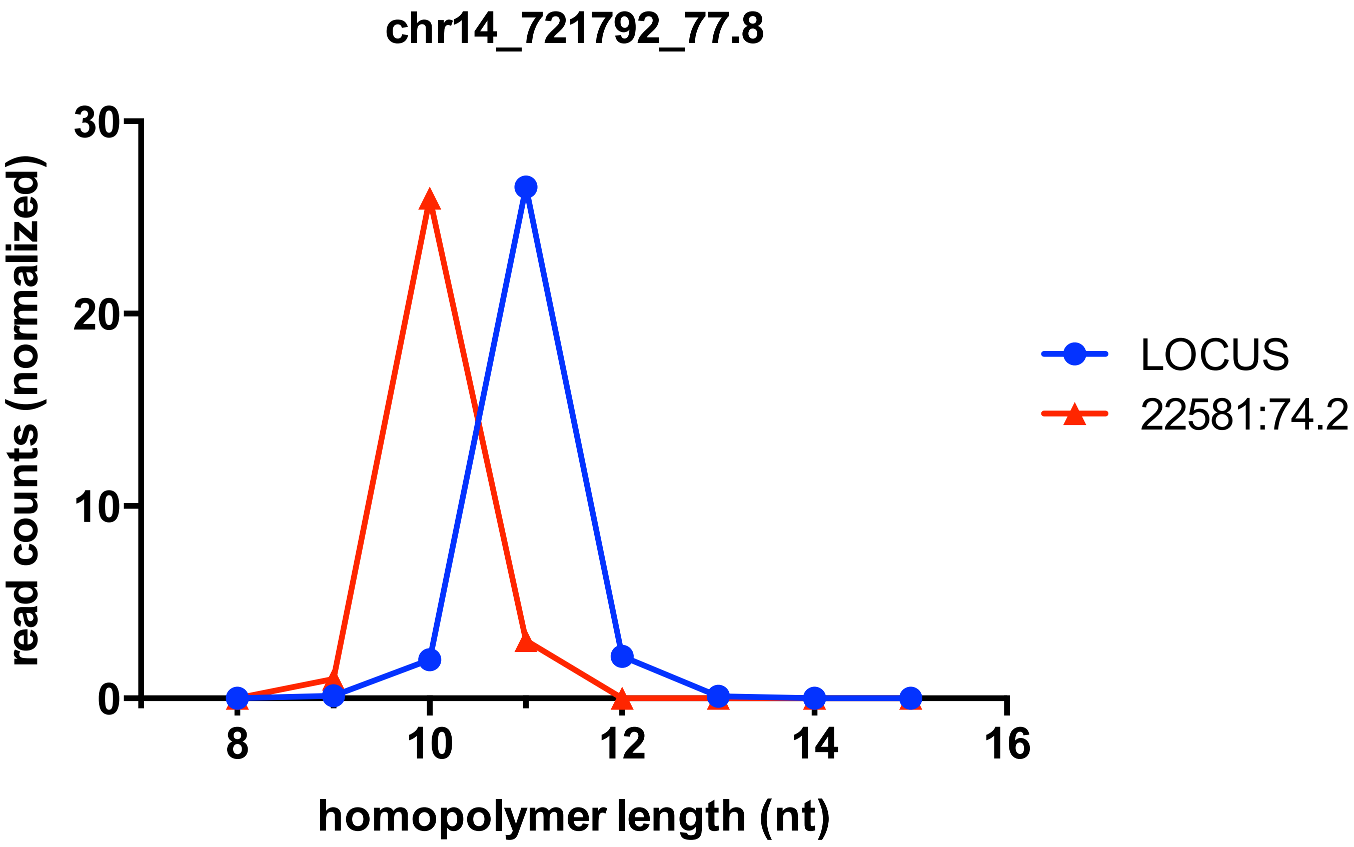
chr14_721792_77.8
read counts (normalized)
30
20
10
0
8
10
12
14
16
homopolymer length (nt)
LOCUS
22581:74.2

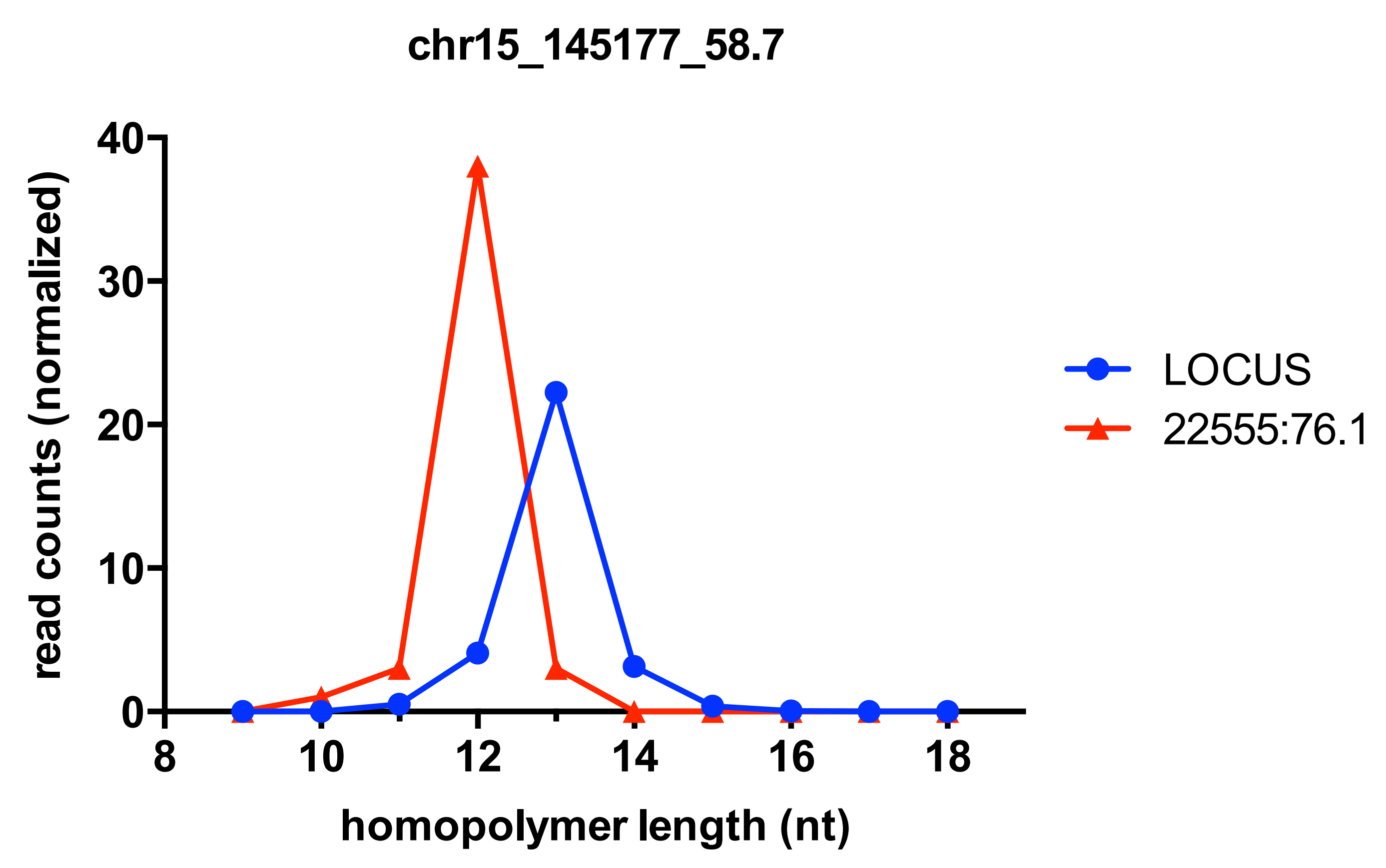

chr15_145177_58.7
read counts (normalized)
40
30
20
10
0
8
10
12
14
16
18
homopolymer length (nt)
LOCUS
22555:76.1

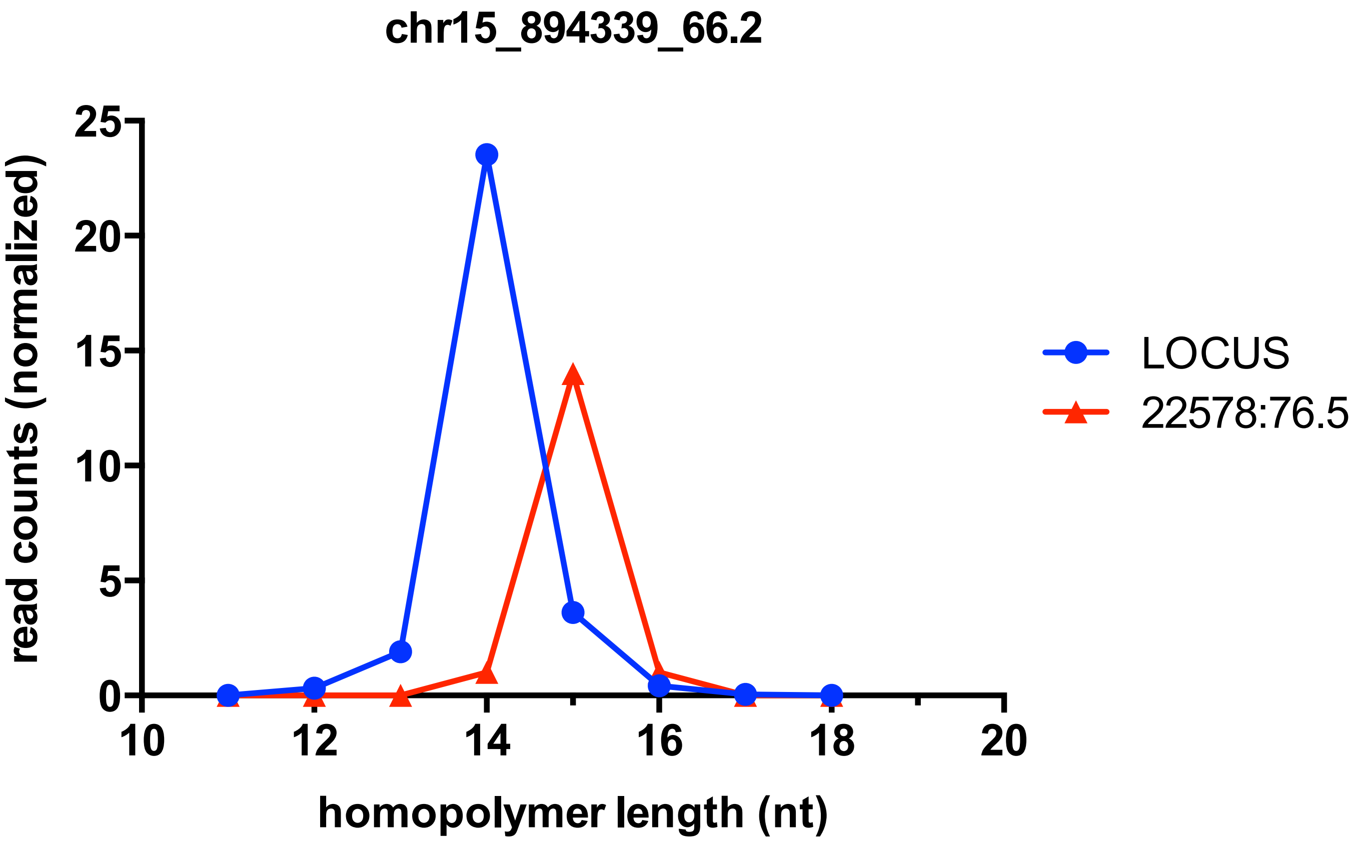
chr15_894339_66.2
read counts (normalized)
25
20
15
10
5
0
10
12
14
16
18
20
homopolymer length (nt)
LOCUS
22578:76.5

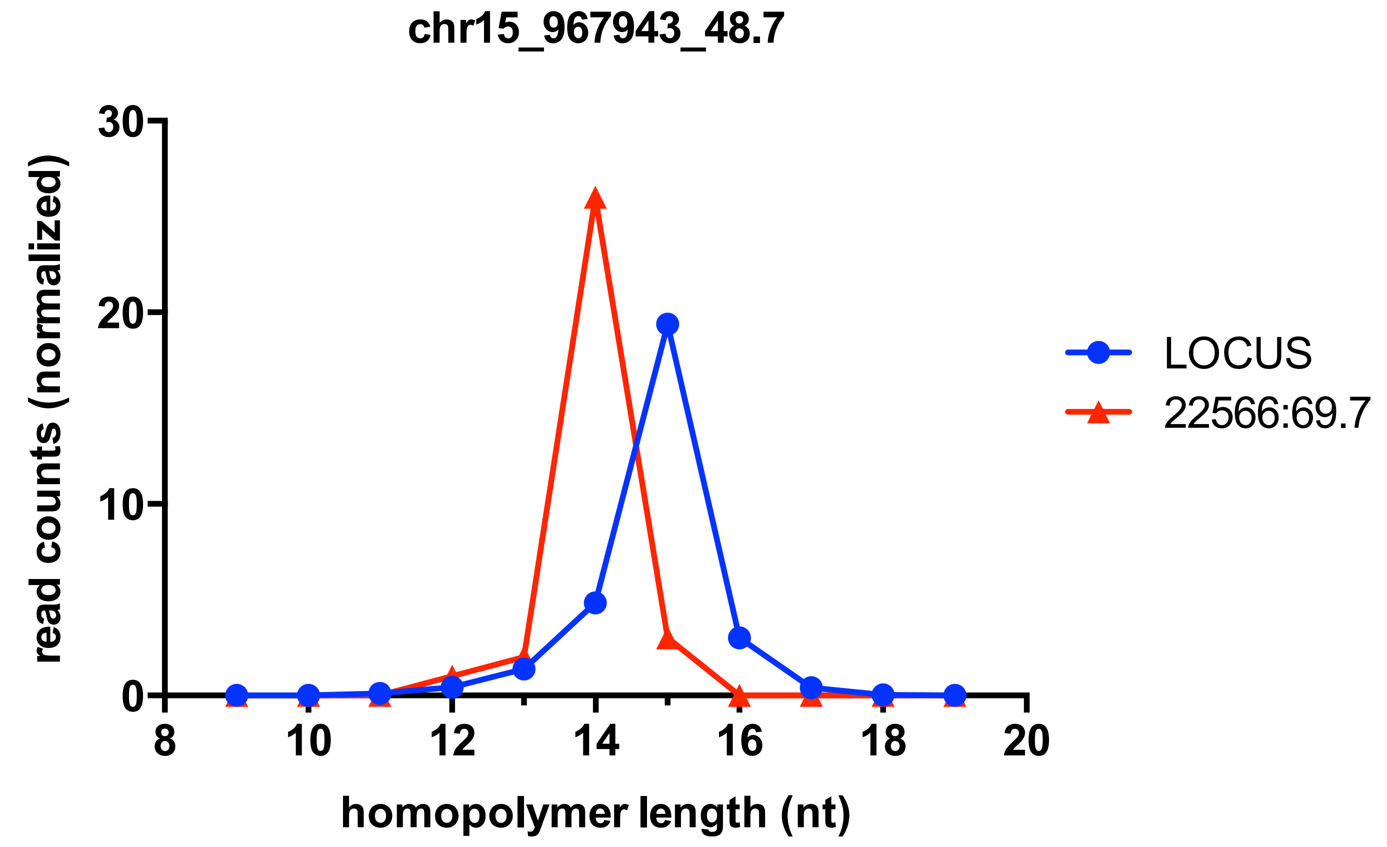

chr15_967943_48.7
read counts (normalized)
30
20
10
0
8
10
12
14
16
18
20
homopolymer length (nt)
LOCUS
22566:69.7

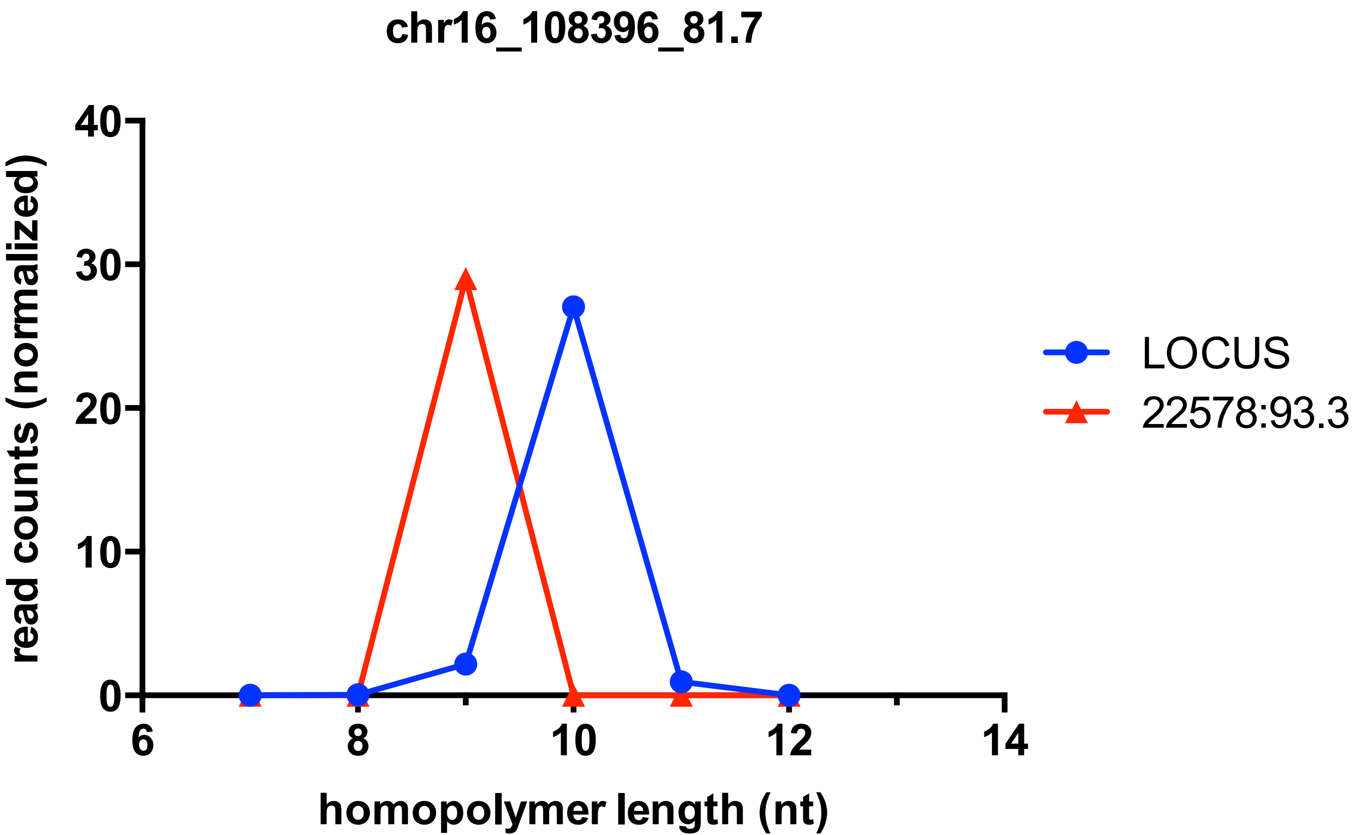
chr16_108396_81.7
read counts (normalized)
40
30
20
10
0
6
8
10
12
14
homopolymer length (nt)
LOCUS
22578:93.3

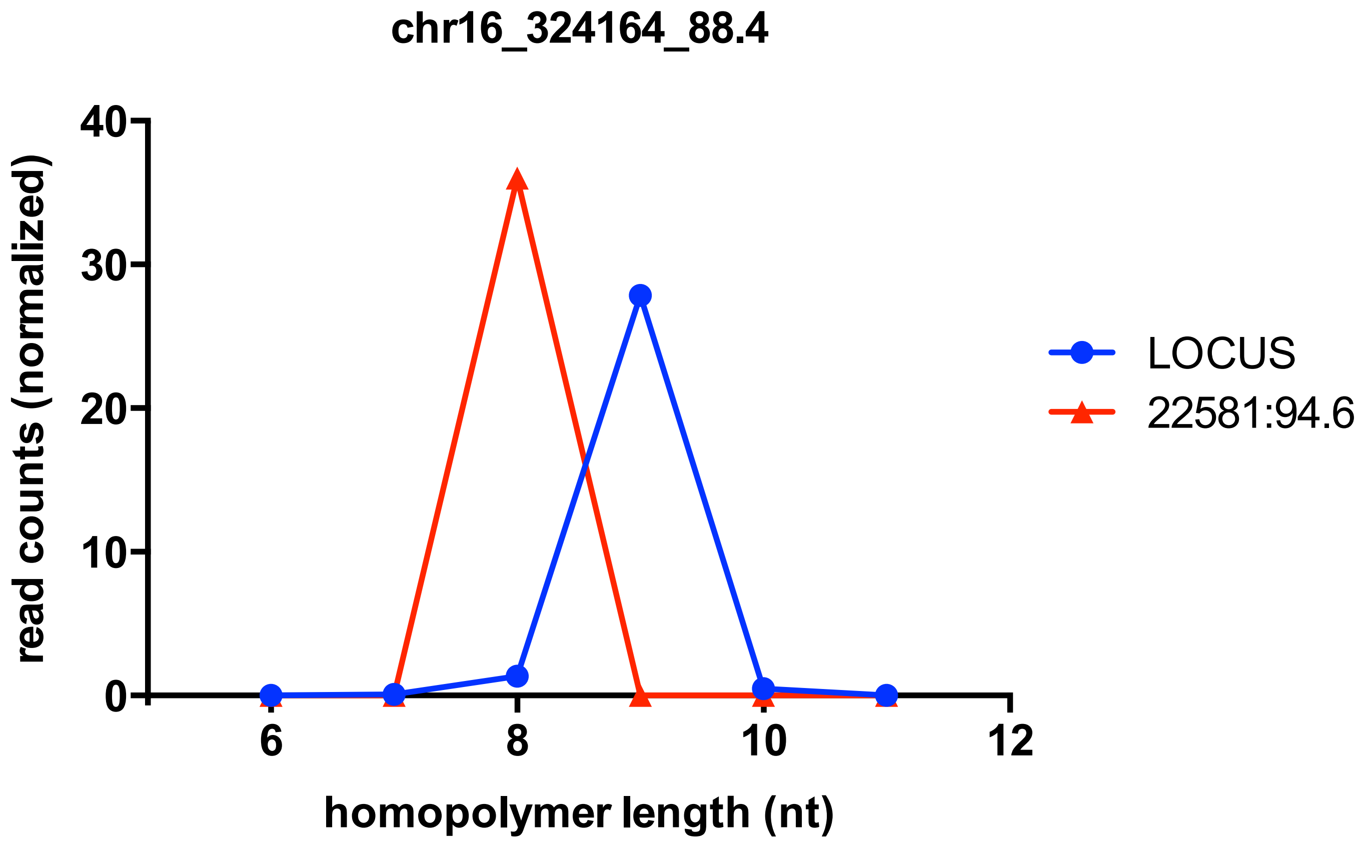
chr16_324164_88.4
read counts (normalized)
40
30
20
10
0
6
8
10
12
homopolymer length (nt)
LOCUS
22581:94.6

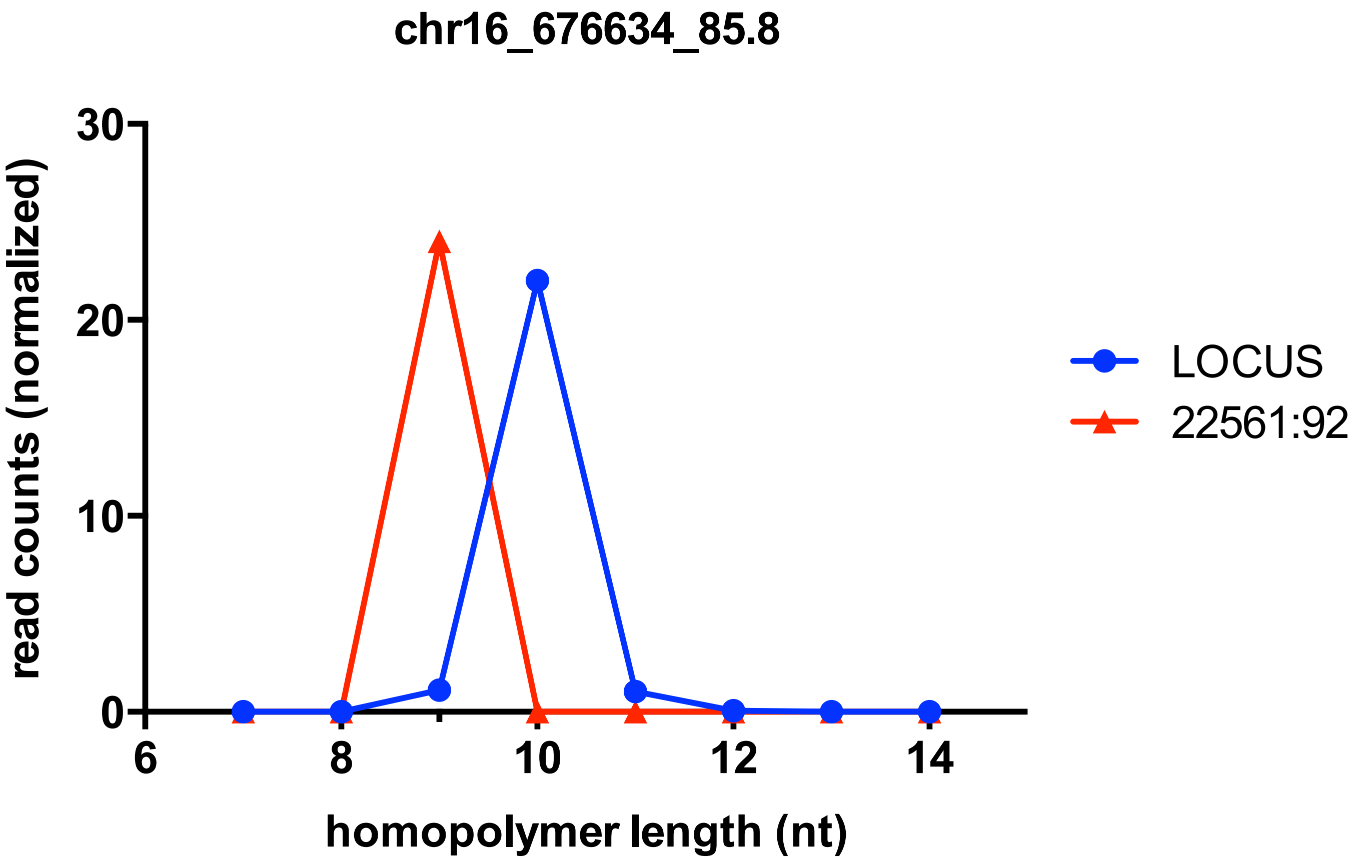

chr16_676634_85.8
read counts (normalized)
30
20
10
0
6
8
10
12
14
homopolymer length (nt)
LOCUS
22561:92

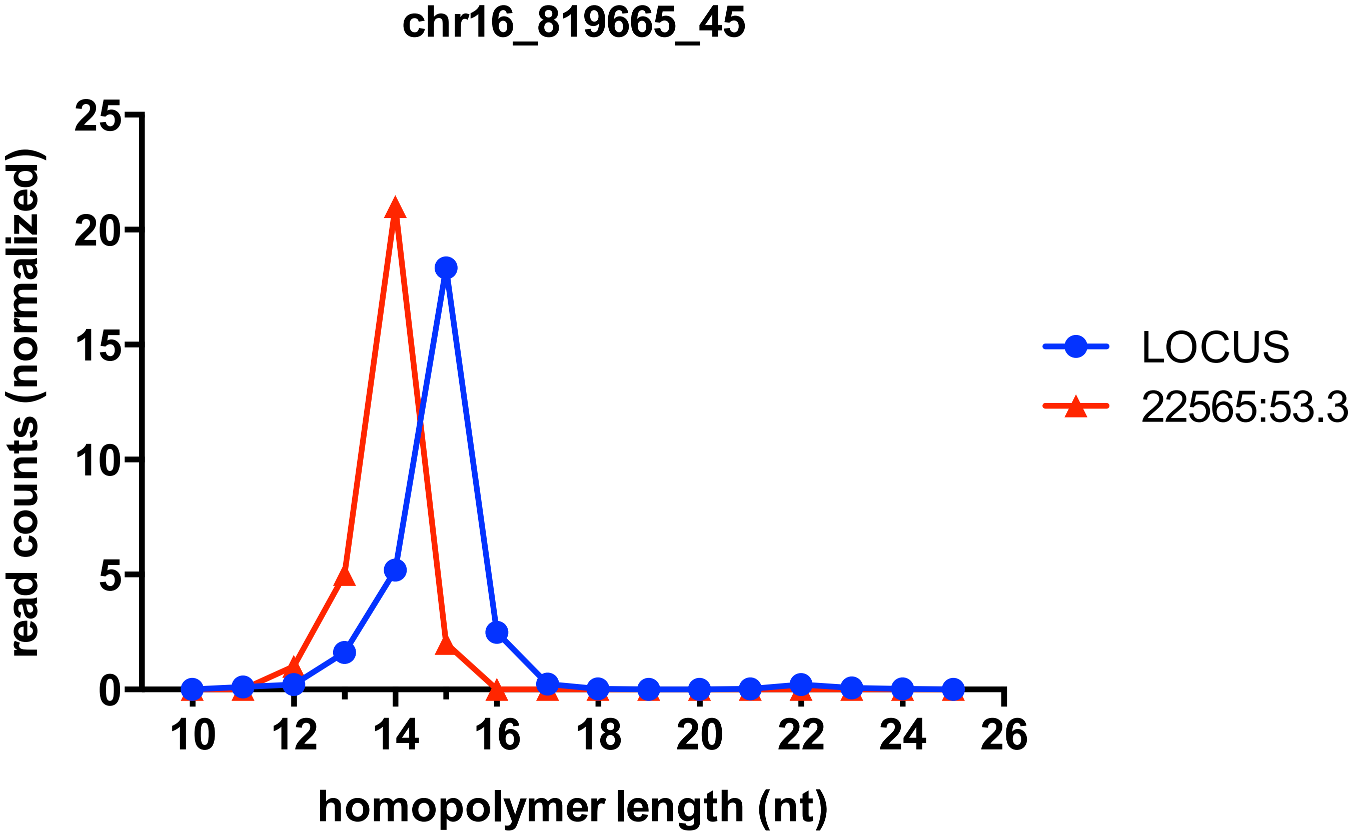
chr16_819665_45
read counts (normalized)
25
20
15
10
5
0
10
12
14
16
18
20
22
24
26
homopolymer length (nt)
LOCUS
22565:53.3

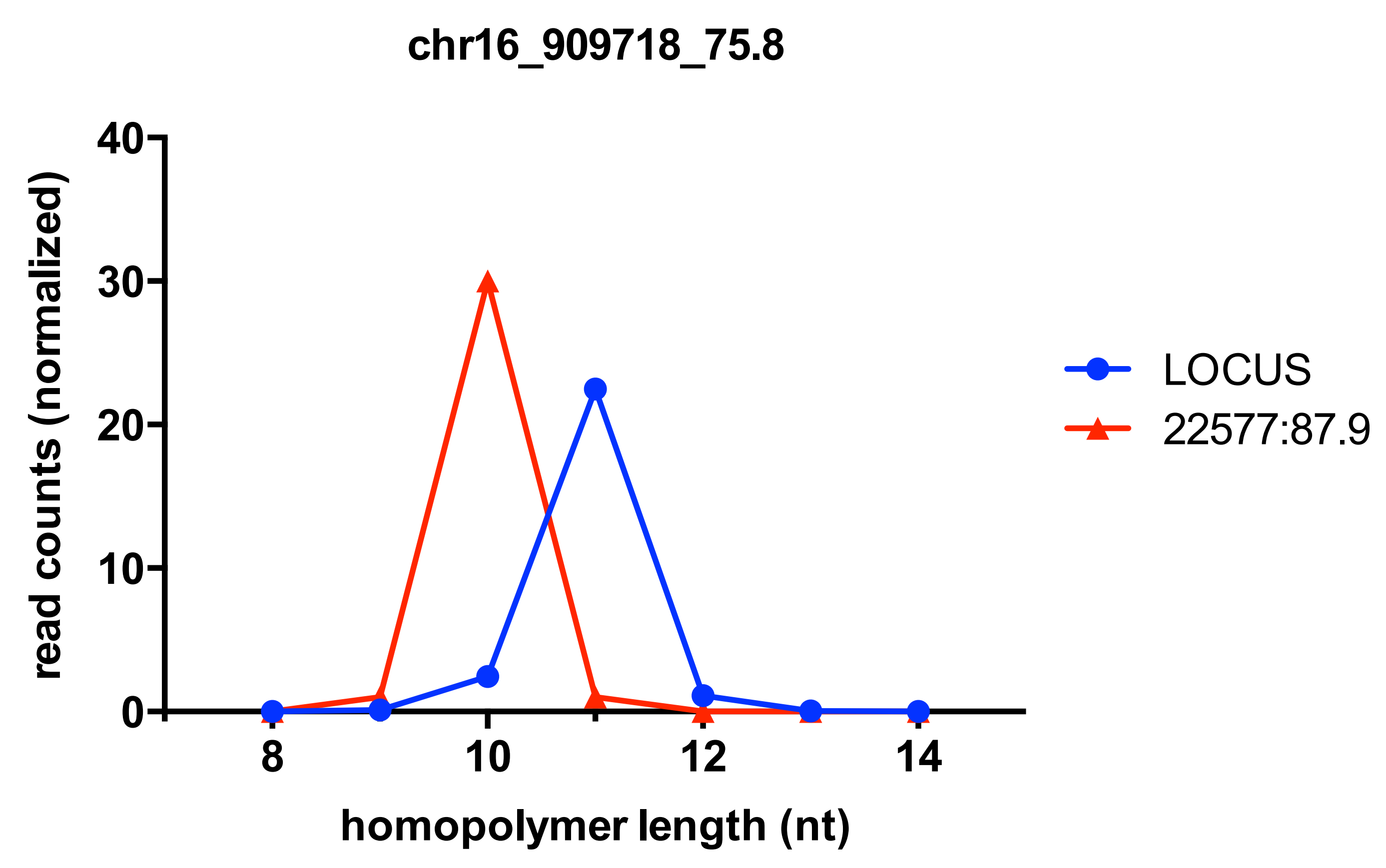
chr16_909718_75.8
read counts (normalized)
0
10
20
30
40
8
10
12
14
homopolymer length (nt)
LOCUS
22577:87.9
